# Supplementary material for: Dysfunctional one-carbon metabolism identifies vitamins B6, B9, B12, and choline as neuroprotective in glaucoma
Source: Cell Rep Med. 2025 May 8;6(5):102127. doi: 10.1016/j.xcrm.2025.102127 (PMC12147907; doi:10.1016/j.xcrm.2025.102127)
Supplement: Document S1. Figures S1–S3 and Tables S1–S4, S6, and S7 [file mmc1.pdf]

Cell Reports Medicine, Volume *6*

Supplemental information

Dysfunctional one-carbon metabolism identifies vitamins B_6, B_9, B_{12}, and choline as neuroprotective in glaucoma

James R. Tribble, Vickie H.Y. Wong, Kelsey V. Stuart, Glyn Chidlow, Alan Nicol, Anne Rombaut, Alessandro Rabiolo, Anh Hoang, Pei Ying Lee, Carola Rutigliani, Tim J. Enz, Alessio Canovai, Emma Lardner, Gustav Stålhammar, Christine T.O. Nguyen, David F. Garway-Heath, Robert J. Casson, Anthony P. Khawaja, Bang V. Bui, and Pete A. Williams

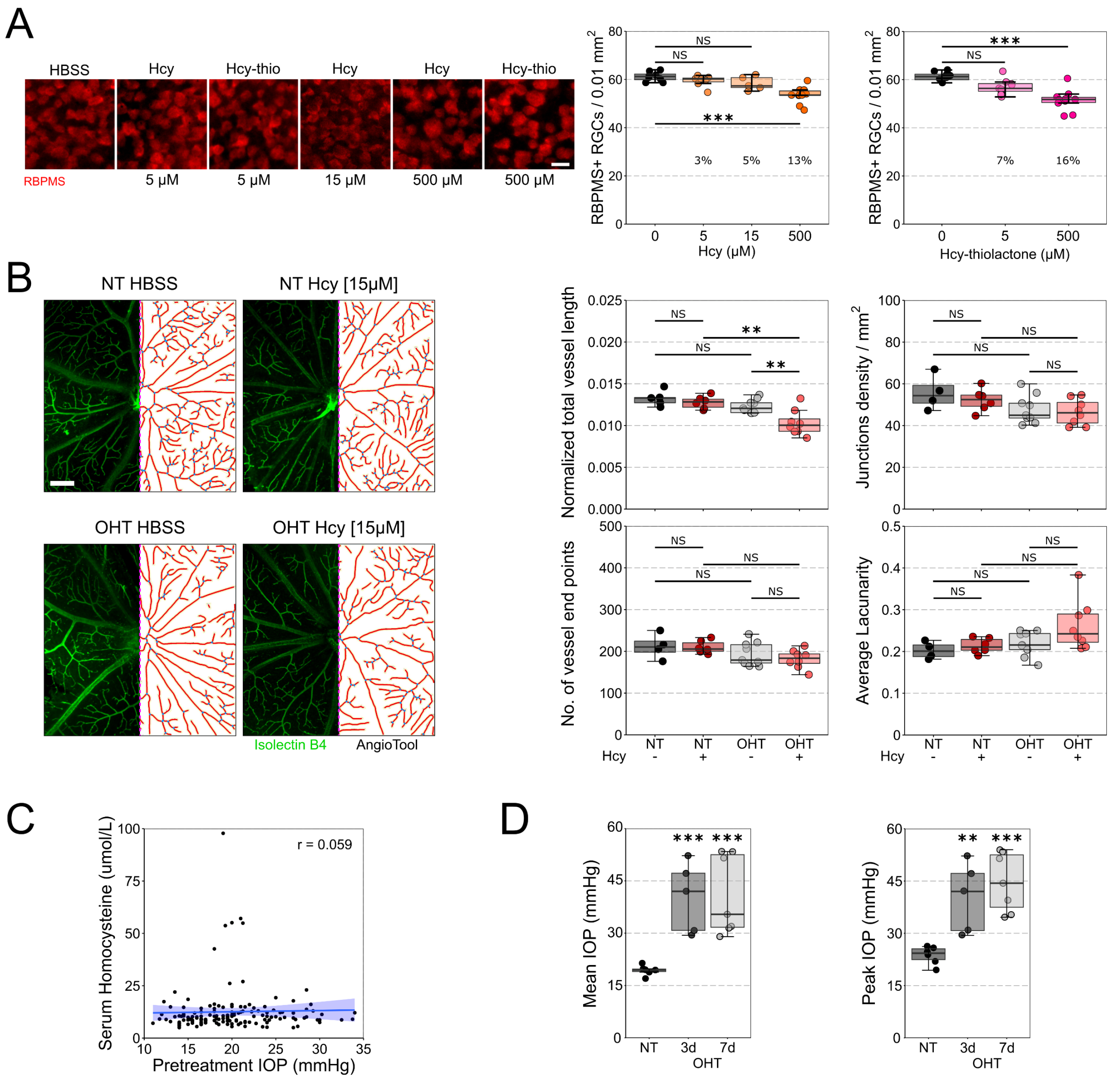

Figure S1: Additional results for the effects of homocysteine on retinal health, related to Figure 1 and 2. A) Mice received an intravitreal injection of homocysteine (Hcy) or homocysteine-thiolactone (Hcy-thiol) to yield a concentration in the vitreous of 5 µM, 15 µM or 500 µM. At 7 days post injection, RGC density was not significantly changed by Hcy at 5 µM or 15 µM, but was significantly reduced by Hcy at 500 µM compared to HBSS only (vehicle) controls. This was repeated for Hcy-thiol (HBSS, *n* = 8 retina; Hcy-5µM, *n* = 7 retina; Hcy-15µM, *n* = 5 retina; Hcy-500µM, *n* = 10 retina; Hcy-thiol-5µM, *n* = 9 retina; Hcy-thiol-500µM, *n* = 10 retina). **B**) Rats received an intravitreal injection of HBSS or homocysteine (Hcy; to a final concentration of 15 µM) 3 days prior to OHT induction. Blood vessel morphology (Isolectin B4+) in the central retina was reconstructed and analyzed (AngioTool). Normalized vessel length was significantly reduced in OHT-Hcy eyes relative to NT-HCY and OHT-HBSS eyes, while junction density (*blue spots*), the number of vessel end points, and lacunarity were not significantly changed. Together, this suggests a shrinkage of small capillaries without vessel drop-out (NT-HBSS, *n* = 4 retina; NT-Hcy, *n* = 6 retina; OHT-HBSS, *n* = 9 retina; OHT-Hcy, *n* = 8 retina). **C**) To determine whether serum homocysteine was associated with higher IOP we performed a linear regression of baseline IOP and baseline serum homocysteine for all patients in the UKGTS cohort and identified no significant association (r = 0.059, *P* = 0.753). Shaded area = confidence interval (*n* = 147 patients). Related to Figure 2, **D**) Rats were induced with OHT for 3 days (3d) or 7 days (7d) or remained NT. Mean and peak IOP were significantly greater in OHT eyes than in NT eyes, and there was no significant difference between 3 and 7 days (NT, *n* = 6 eyes; OHT-d3, *n* = 6 eyes; OHT-d7, *n* = 7 eyes).Eyes were paraffin embedded and sectioned for immunofluorescent labelling. Scale bar = 20

μm in A and 250 μm in B**.** * = $P < 0.05$, ** = $P < 0.01$, *** = $P < 0.001$, NS = $P > 0.05$. For box plots, the center hinge represents the median with upper and lower hinges representing the first and third quartiles; whiskers represent 1.5 times the interquartile range.

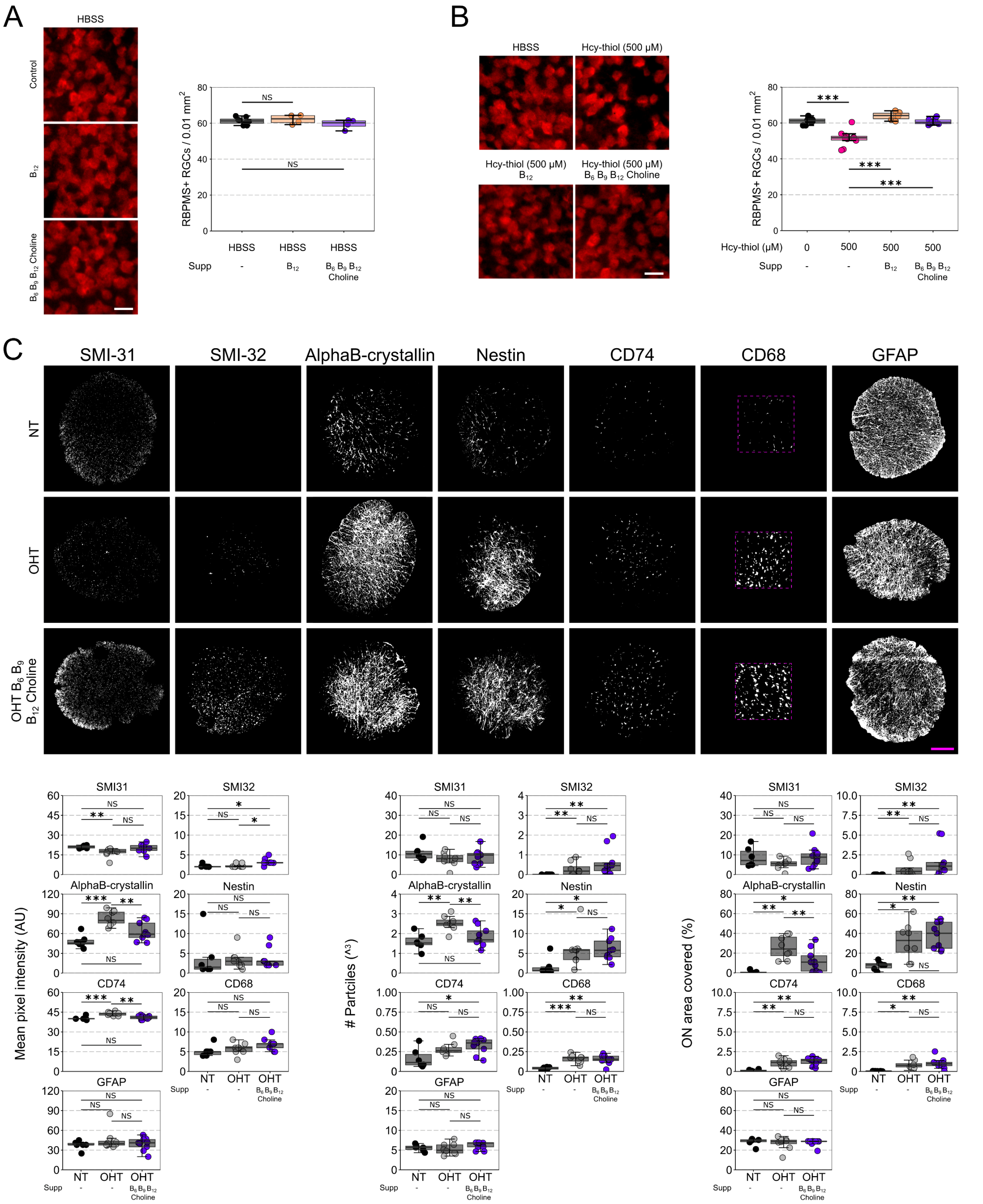

Figure S2: Additional results for the effects of B_6, B_9, B_{12}, and Choline on neurodegeneration in the mouse model of elevated ocular homocysteine and rat bead model, related to Figure 4. A-B) Mice received either 20 µg/kg/day vitamin B_{12} only or B_{12} with 4.5 mg/kg/day vitamin B_6, 1.5 mg/kg/day vitamin B_9, and 750 mg/kg/d Choline in their drinking water 7 days prior to intravitreal injection with either HBSS () or Homocysteine-thiolactone (Hcy-thiol; to a final concetration of 500 µM in the vitreous). **A**) Supplementation had no effect on RGC density after 14 days (HBSS-vehicle, *n* = 8 retina; HBSS-B_{12}, *n* = 4 retina; HBSS-B_6/B_9/B_{12}/Choline, *n* = 4 retina). **B**) Hcy-thio resulted in a significant decrease in RGC density 7 days after injection, which was completely prevented by vitamin B_{12} only or B_6, B_9, B_{12}, and Choline (7 days pre-treatment, continuing for the 7 days post injection; HBSS-vehicle, *n* = 8 retina; Hcy-thiol, *n* = 10 retina; Hcy-thiol-B_{12}, *n* = 6 retina; Hcy-thiol-B_6/B_9/B_{12}/Choline, *n* = 8 retina). **C**) Rats received the same doses 7 days prior to OHT induction and

optic nerves were collected following 14 days. IF/IHC labeling of SMI31 (healthy axons), SMI32 (degenerating axons), Alphaβ-crystallin and Hsp27 (stress), and Cd74, Cd68, and GFAP (inflammation) was performed and quantified by mean pixel intensity, the number of disconnected particles, and the percentage of the ON cross sectional area covered by positive labelling. We identified significant protection of SMI31 intensity by B_6, B_9, B_{12}, and Choline, but no prevention of SMI32 accumulation supporting a potential delay to axon degeneration. Alphaβ-crystallin was significantly reduced in B_6, B_9, B_{12}, and Choline relative to untreated OHT, supporting reduced stress. Markers of inflammation were generally not improved by B_6, B_9, B_{12}, and Choline relative to untreated OHT except for the intensity of Cd74 labelling, which was significantly reduced by B_6, B_9, B_{12}, and Choline (HT-vehicle n = 6 ONs; OHT-vehicle n = 8 ONs; OHT-B_6/B_9/B_{12}/choline n = 9 ONs) Scale bar = 20 µm in A and B, 100 µm in C**.** * = $P < 0.05$, ** = $P < 0.01$, *** = $P < 0.001$, NS = $P > 0.05$. For box plots, the center hinge represents the median with upper and lower hinges representing the first and third quartiles; whiskers represent 1.5 times the interquartile range.

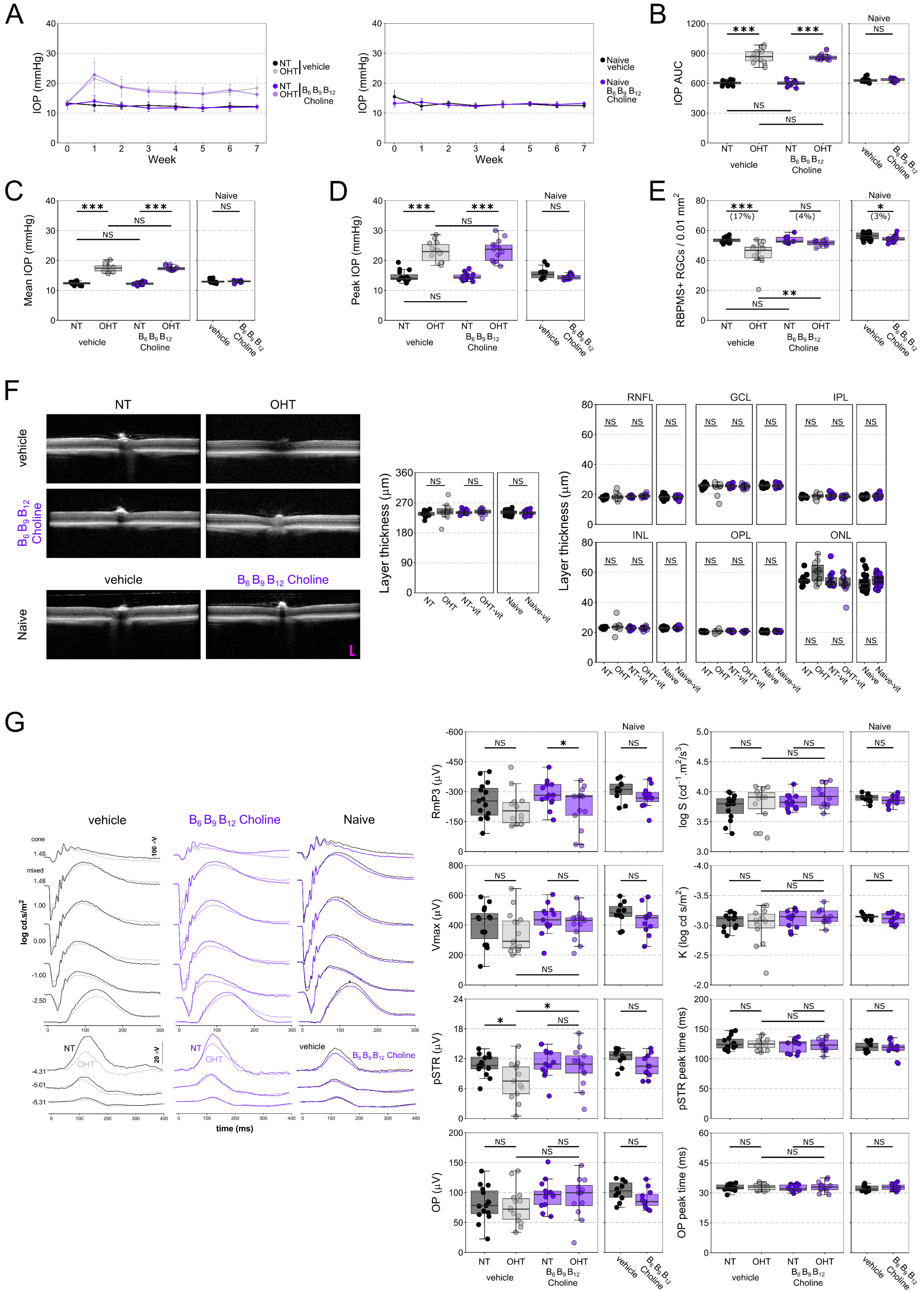

Figure S3: Additional results for the effects of B6, B9, B12, and Choline on neurodegeneration in the mouse circumlimbal suture model, related to Figure 4. A) Mice received 4.5 mg/kg/day vitamin B_6, 1.5 mg/kg/day vitamin B_9, 20 µg/kg/day vitamin B_{12}, and 750 mg/kg/d Choline (or vehicle only) in drinking water 1 week prior to OHT induction unilateral OHT (unilateral circumlimbal suture),

and continuing for the 7 week duration of OHT. Two naïve cohorts of mice were either vehicle treated or received B_6, B_9, B_{12}, and Choline. **A-D**) OHT was maintained for 7 weeks and the area under curve of the the IOP profile (**B**), the mean IOP (**C**), and the peak IOP (**D**) were not significantly altered by supplementation in OHT or NT eyes (*n* = 20 eyes from 10 mice, Naïve-vehicle; *n* = 22 eyes from 11 mice, Naïve-B_6/B_9/B_{12}/Choline; for *n* of other conditions see Figure 4). **E**) RGC density was significantly reduced in untreated OHT eyes, and this was completely protected against by B_6, B_9, B_{12}, and Choline. In naïve B_6, B_9, B_{12}, and Choline treated animals, RGC density was significantly reduced but the biological relevance of this is likely limited given the 3% difference to untreated naïve controls (*n* = 20 retina from 10 mice, Naïve-vehicle; *n* = 22 retina from 11 mice, Naïve-B_6/B_9/B_{12}/Choline; for *n* of other conditions see Figure 4). **F**) *In vivo* OCT imaging was performed to assess changes to retinal morphology. There were no significant changes in total retinal thickness or individual layer thicknesses across all conditions, consistent with mild OHT induced degeneration and lack of obvious toxic effects from supplementation (*n* = 24 eyes from 12 mice, Naïve-vehicle; *n* = 22 eyes from 11 mice, Naïve-B_6/B_9/B_{12}/Choline; *n* = 12 eyes NT-vehicle paired with *n* = 10 eyes OHT-vehicle from 12 mice ; *n* = 13 eyes NT-B_6/B_9/B_{12}/Choline paired with *n* = 11 eyes OHT-B_6/B_9/B_{12}/Choline from 13 mice). **G**) *In vivo* electrophysiological function was assessed by ERG. In naïve animals, B_6, B_9, B_{12}, and Choline had no effect on function. In untreated OHT eyes, pSTR amplitude was significantly decreased, and this was prevented in B_6, B_9, B_{12}, and Choline treated animals, supporting a protection to RGC specific visual function. Supplementation did cause a reduction in RmP3 amplitude, in treated eyes suggesting a decrease in photoreceptor scotopic responses (*n* = 10 eyes from 10 mice, Naïve-vehicle; *n* = 11 eyes from 11 mice, Naïve-B_6/B_9/B_{12}/Choline; for *n* of other conditions see Figure 4). Scale bar = 200 µM in F. * = $P < 0.05$, ** = $P < 0.01$, *** = $P < 0.001$, NS = $P > 0.05$. For box plots, the center hinge represents the median with upper and lower hinges representing the first and third quartiles; whiskers represent 1.5 times the interquartile range.

Trait	Source	Number of participants	Participant ethnicity	SNPs included in the instrumental variable
Exposure				
Homocysteine	van Meurs (2013) [S1]	44,147	European	14
Outcome				
Macular retinal nerve fiber layer thickness	Currant (2021) [S2]	31,434	European	-
Macular ganglion cell-inner plexiform layer thickness	Currant (2021) [S2]	31,434	European	-
Intraocular pressure	Khawaja (2018) [S3]	139,555	European	-
Vertical cup-disc ratio (adjusted for vertical disc diameter)	Han (2021) [S4]	111,724	European	-
Primary open-angle glaucoma	Gharahkhani (2021) [S5]	216,257	European	-

Table S1: Details of summary-level data used for Mendelian randomization analyses, related to Figure 1. SNP, single nucleotide polymorphism.

**MR method**	**mRNFL (μm)**		**mGCIPL (μm)**		**IOP (mmHg)**		**vCDR**		**POAG**	
	Beta (95% CI)	*P*	Beta (95% CI)	*P*	Beta (95% CI)	*P*	Beta (95% CI)	*P*	Odds ratio (95% CI)	*P*
**Homocysteine**										
IVW	-0.21 (-0.49, 0.08)	0.16	-0.30 (-0.81, 0.21)	0.25	0.03 (-0.16, 0.22)	0.77	0.00 (-0.01, 0.00)	0.39	1.12 (0.93, 1.27)	0.06
Weighted median	-0.18 (-0.57, 0.21)	0.37	-0.46 (-0.98, 0.07)	0.09	-0.06 (-0.21, 0.08)	0.40	0.00 (-0.01, 0.01)	0.71	1.13 (0.98, 1.30)	0.11
MR-Egger	-0.15 (-0.81, 0.51)	0.65	-0.55 (-1.73, 0.63)	0.36	0.09 (-0.35, 0.53)	0.70	0.00 (-0.02, 0.01)	0.66	1.02 (0.78, 1.35)	0.86
MR-PRESSO	-	-	-	-	-0.09 (-0.18, 0.00)	0.07	0.00 (-0.01, 0.00)	0.16	-	-

Table S2: Results of Mendelian randomization analyses, related to Figure 1. MR-PRESSO produces estimates after removal of significant outlying variants; if there are no significant outliers, the estimate is the same as that from the IVW method. IOP, intraocular pressure; IVW, inverse-variance weighted; MR, mendelian randomization; mRNFL, macular retinal nerve fiber layer; MR-PRESSO, Mendelian Randomization Pleiotropy RESidual Sum and Outlier; mGCIPL, macular ganglion cell-inner plexiform layer; POAG, primary open-angle glaucoma; vCDR, vertical cup-disc ratio.

SNP	Chr	BP	RA	EA	EAF	Nearest gene	Homocysteine		mRNFL		mGCIPL		IOP		vCDR		POAG	
							Beta	SE	Beta	SE	Beta	SE	Beta	SE	Beta	SE	Beta	SE
rs1801133	1	11856378	G	A	0.34	*MTHFR*	0.1583	0.0070	-0.0161	0.0382	-0.0771	0.0502	-0.0100	0.0136	-0.0003	0.0014	0.0190	0.0145
rs4660306	1	45978675	C	T	0.33	*MMACHC*	0.0435	0.0070	-0.0169	0.0381	-0.0545	0.0501	-0.0006	0.0135	0.0005	0.0014	0.0347	0.0135
rs2275565	1	237048676	T	G	0.79	MTR	0.0542	0.0090	0.0131	0.0441	-0.0084	0.0579	0.0158	0.0159	-0.0006	0.0016	-0.0049	0.0155
rs1047891	2	211540507	C	A	0.33	*CPS1*	0.0860	0.0080	0.0397	0.0388	0.1024	0.0510	-0.0255	0.0141	-0.0019	0.0015	0.0310	0.0139
rs548987	6	25869371	G	C	0.13	*SLC17A3*	0.0597	0.0100	0.0003	0.0517	0.1015	0.0679	-	-	-	-	-	-
rs9369898	6	49382193	G	A	0.62	*MUT*	0.0449	0.0070	-0.0188	0.0376	-0.0424	0.0494	-0.0050	0.0133	-0.0015	0.0014	0.0318	0.0133
rs42648	7	89977760	A	G	0.60	*GTPB10*	0.0395	0.0070	-0.0565	0.0371	-0.0434	0.0488	0.0042	0.0132	0.0008	0.0014	-0.0021	0.0131
rs1801222	10	17156151	G	A	0.34	*CUBN*	0.0453	0.0070	-0.0654	0.0374	-0.0324	0.0492	0.0150	0.0133	0.0028	0.0014	-0.0019	0.0135
rs12780845	10	17223244	G	A	0.65	*CUBN*	0.0529	0.0090	-0.0017	0.0392	-0.0406	0.0515	-0.0185	0.0138	-0.0012	0.0014	0.0063	0.0139
rs7130284	11	89148372	T	C	0.93	*NOX4*	0.1242	0.0130	0.1057	0.0689	0.1190	0.0905	-0.0091	0.0242	0.0008	0.0023	-0.0060	0.0235
rs2251468	12	121405126	A	C	0.35	*HNF1A*	0.0510	0.0070	0.0689	0.0383	0.0172	0.0503	0.0025	0.0136	0.0002	0.0014	0.0049	0.0134
rs154657	16	89708096	G	A	0.47	*DPEP1*	0.0963	0.0070	-0.0466	0.0367	-0.1124	0.0482	0.0709	0.0133	-0.0014	0.0014	0.0016	0.0133
rs838133	19	49259529	G	A	0.45	*FUT2*	0.0422	0.0070	0.0270	0.0375	0.1379	0.0493	-	-	0.0004	0.0017	0.0061	0.0137
rs234709	21	44486964	T	C	0.55	*CBS*	0.0718	0.0070	0.0259	0.0365	0.0044	0.0479	-0.0101	0.0130	-0.0021	0.0012	-0.0158	0.0142

Table S3: Details of SNPs included in the instrumental variables and their associations with glaucoma and related traits, related to Figure 1. rs1801133 (highlighted in green) is located in the methylenetetrahydrofolate reductase (MTHFR) gene region, a key enzyme in homocysteine metabolism. This SNP has the single strongest association with plasma homocysteine levels and is often used as an instrumental variable in MR analyses. BP, base position (build 37); Chr, chromosome; EA, effect allele; EAF, effect allele frequency; IOP, intraocular pressure; mGCIPL, macular ganglion cell-inner plexiform layer; mRNFL, macular retinal nerve fiber layer; POAG, primary open-angle glaucoma; RA, reference allele; SE, standard error; SNP, single nucleotide polymorphism; vCDR, vertical cup-disc ratio.

MR method	mRNFL		mGCIPL		IOP		vCDR		POAG	
	Statistic	*P*	Statistic	*P*	Statistic	*P*	Statistic	*P*	Statistic	*P*
**Homocysteine**										
**IVW**										
Cochran's *Q* statistic	13.3 (13)	0.43	25.0 (13)	0.02	37.0 (11)	<0.001	23.3 (12)	0.026	16.3 (12)	0.18
I^2 statistic	2.2%	-	48.0%	-	70.3%	-	48.4%	-	26.6%	-
**MR-Egger**										
Rucker's *Q'* statistic	13.3 (12)	0.35	24.5 (12)	0.02	36.7 (10)	<0.001	23.2 (11)	0.016	15.6 (11)	0.16
I^2_{GX} statistic	95.3%	-	95.3%	-	95.6%	-	93.2%	-	95.1%	-
Intercept	0.00	0.86	0.02	0.64	-0.01	0.77	0.00	0.92	0.01	0.47
**MR-PRESSO**										
Global test	-	0.48	-	0.03	-	<0.001	-	0.038	-	0.23
Number of outliers	0	-	0	-	1	-	1	-	0	-
Distortion test	-	-	-	-	131.1%	0.27	32.8%	0.70	-	-

Table S4: Tests of heterogeneity, directional pleiotropy and regression dilution statistics, related to Figure 1. IOP, intraocular pressure; IVW, inverse-variance weighted; MR, mendelian randomization; mRNFL, macular retinal nerve fiber layer; MR-PRESSO, Mendelian Randomization Pleiotropy RESidual Sum and Outlier; mGCIPL, macular ganglion cell-inner plexiform layer; POAG, primary open-angle glaucoma; vCDR, vertical cup-disc ratio.

Intake	mRNFL (µm)			mGCIPL (µm)			Glaucoma		
	Beta	(95% CI)	*P*	Beta	(95% CI)	*P*	OR	(95% CI)	*P*
**Vitamin B6 (mg)**	(*n* = 11,771)			(*n* = 11,751)			(*n* = 27,191)		
Per SD increase	-0.05	(-0.15, 0.06)	0.39	-0.10	(-0.21, 0.02)	0.10	1.01	(0.89, 1.15)	0.86
Q1 (<1.64)	Reference			Reference			Reference		
Q2 (1.64–1.99)	**0.20**	**(0.01, 0.40)**	**0.042**	0.14	(-0.12, 0.39)	0.30	0.93	(0.70, 1.24)	0.61
Q3 (1.99–2.40)	0.11	(-0.10, 0.31)	0.32	0.05	(-0.22, 0.32)	0.73	1.12	(0.83, 1.49)	0.46
Q4 (>2.40)	-0.04	(-0.27, 0.20)	0.76	-0.12	(-0.43, 0.18)	0.43	1.00	(0.72, 1.39)	0.99
P (trend)			0.63			0.38			0.71
**Folate (µg)**	(*n* = 11,771)			(*n* = 11,751)			(*n* = 27,191)		
Per SD increase	-0.07	(-0.15, 0.01)	0.10	-0.09	(-0.20, 0.02)	0.10	1.04	(0.93, 1.17)	0.49
Q1 (<247.2)	Reference			Reference			Reference		
Q2 (247.2–302.5)	**0.21**	**(0.01, 0.40)**	**0.035**	0.05	(-0.20, 0.31)	0.68	0.91	(0.68, 1.21)	0.51
Q3 (302.5–366.3)	**0.22**	**(0.02, 0.42)**	**0.034**	0.19	(-0.08, 0.45)	0.16	0.92	(0.68, 1.23)	0.57
Q4 (>366.3)	-0.07	(-0.29, 0.16)	0.56	-0.13	(-0.43, 0.16)	0.37	1.06	(0.77, 1.45)	0.72
P (trend)			0.66			0.63			0.68
**Vitamin B12 (µg)**	(*n* = 11,771)			(*n* = 11,751)			(*n* = 27,191)		
Per SD increase	-0.02	(-0.09, 0.06)	0.63	-0.01	(-0.11, 0.08)	0.78	0.91	(0.81, 1.01)	0.08
Q1 (<4.35)	Reference			Reference			Reference		
Q2 (4.35–5.74)	-0.07	(-0.27, 0.12)	0.46	0.00	(-0.25, 0.26)	0.98	0.85	(0.64, 1.12)	0.24
Q3 (5.74–7.46)	-0.02	(-0.22, 0.18)	0.86	0.20	(-0.06, 0.46)	0.13	0.83	(0.62, 1.09)	0.18
Q4 (>7.46)	-0.07	(-0.28, 0.13)	0.49	0.07	(-0.20, 0.33)	0.63	0.81	(0.61, 1.08)	0.16
P (trend)			0.64			0.37			0.18

Table S6: Dietary intake of B_6, B_9, B_{12} with glaucoma related-traits, related to Figure 4. Dietary intake of B_6, folate (B_9) and B_{12} multivariable linear (for mRNFL and GCIPL thickness) and logistic (for glaucoma status) regression analyses. CI, confidence interval; mGCIPL, macular ganglion cell-inner plexiform layer; mRNFL, macular retinal nerve fiber layer; OR, odds ratio; Q, quartile; SD, standard deviation.

REAGENT	SOURCE	IDENTIFIER
Ahcy PrimePCR SYBR Green Assay	Bio-rad	10025636
Ahcyl1 PrimePCR SYBR Green Assay	Bio-rad	10025636
Dnmt1 PrimePCR SYBR Green Assay	Bio-rad	10025636
Dnmt3a PrimePCR SYBR Green Assay	Bio-rad	10025636
Dnmt3b PrimePCR SYBR Green Assay	Bio-rad	10025636
Mtr PrimePCR SYBR Green Assay	Bio-rad	10025636
Mtrr PrimePCR SYBR Green Assay	Bio-rad	10025636
Mat1a PrimePCR SYBR Green Assay	Bio-rad	10025636
Mat2b PrimePCR SYBR Green Assay	Bio-rad	10025636
Cbs PrimePCR SYBR Green Assay	Bio-rad	10025636
Cth PrimePCR SYBR Green Assay	Bio-rad	10025636
Dhfr PrimePCR SYBR Green Assay	Bio-rad	10025636
Shmt1 PrimePCR SYBR Green Assay	Bio-rad	10025636
Shmt2 PrimePCR SYBR Green Assay	Bio-rad	10025636
Mthfr PrimePCR SYBR Green Assay	Bio-rad	10025636
Gapdh PrimePCR SYBR Green Assay	Bio-rad	10025636

Table S7: Continuation of Key resources table – Oligonucleotides, related to Key resources table. qPCR primer details.

Supplemental reference list

S1. van Meurs JB, Pare G, Schwartz SM, Hazra A, Tanaka T, Vermeulen SH, et al. Common genetic loci influencing plasma homocysteine concentrations and their effect on risk of coronary artery disease. Am J Clin Nutr. 2013;98(3):668-76.

S2. Currant H, Hysi P, Fitzgerald TW, Gharahkhani P, Bonnemaijer PWM, Senabouth A, et al. Genetic variation affects morphological retinal phenotypes extracted from UK Biobank optical coherence tomography images. PLoS Genet. 2021;17(5):e1009497.

S3. Khawaja AP, Cooke Bailey JN, Wareham NJ, Scott RA, Simcoe M, Igo RP, et al. Genome-wide analyses identify 68 new loci associated with intraocular pressure and improve risk prediction for primary open-angle glaucoma. Nat Genet. 2018;50(6):778-82.

S4. Han X, Steven K, Qassim A, Marshall HN, Bean C, Tremeer M, et al. Automated AI labeling of optic nerve head enables insights into cross-ancestry glaucoma risk and genetic discovery in >280,000 images from UKB and CLSA. Am J Hum Genet. 2021;108(7):1204-16.

S5. Gharahkhani P, Jorgenson E, Hysi P, Khawaja AP, Pendergrass S, Han X, et al. Genome-wide meta-analysis identifies 127 open-angle glaucoma loci with consistent effect across ancestries. Nat Commun. 2021;12(1):1258.
