# Supplementary material for: Dysfunctional one-carbon metabolism identifies vitamins B6, B9, B12, and choline as neuroprotective in glaucoma
Source: Cell Rep Med. 2025 May 8;6(5):102127. doi: 10.1016/j.xcrm.2025.102127 (PMC12147907; doi:10.1016/j.xcrm.2025.102127)
Supplement: Document S2. Article plus supplemental information [file mmc3.pdf]

Cell Reports
Medicine

Article

Dysfunctional one-carbon metabolism identifies vitamins B_6, B_9, B_{12}, and choline as neuroprotective in glaucoma

Graphical abstract

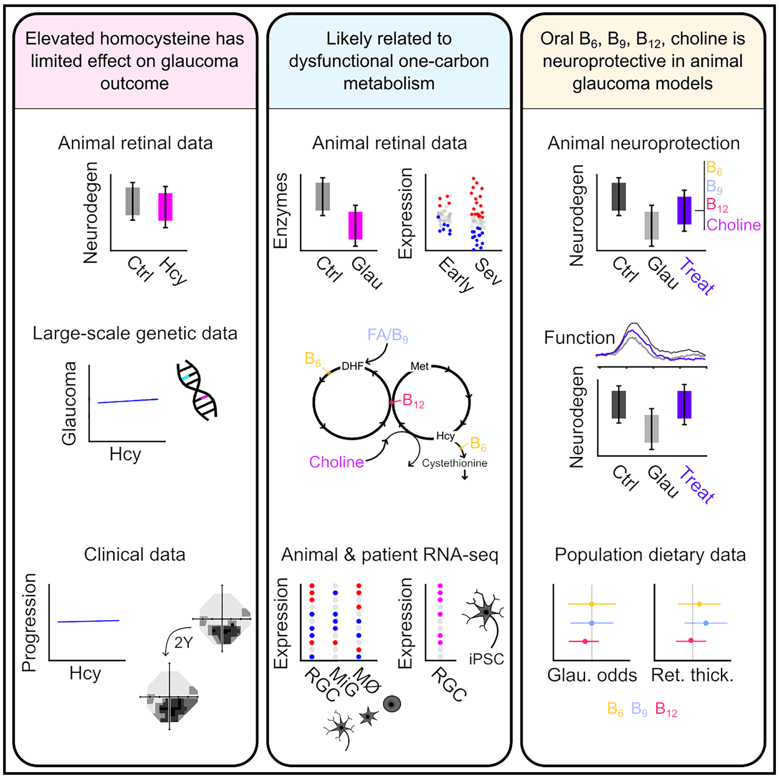

Highlights

- Elevated retinal homocysteine is a pathogenic feature of glaucoma
- One-carbon metabolism is dysregulated in glaucoma
- Oral B_6, B_9, B_{12}, and choline is neuroprotective in animal models

Authors

James R. Tribble, Vickie H.Y. Wong, Kelsey V. Stuart, ..., Anthony P. Khawaja, Bang V. Bui, Pete A. Williams

Correspondence

james.tribble@ki.se (J.R.T.),
pete.williams@ki.se (P.A.W.)

In brief

Tribble et al. identify elevated retinal homocysteine as a pathogenic, rather than causative, feature of glaucoma, likely lined to the dysfunction of one-carbon metabolism. Supplementing a cocktail of B_6, B_9, B_{12}, and choline (cofactors and precursors in this metabolic pathway) provides neuroprotection and visual protection in rodent models of glaucoma.

Tribble et al., 2025, Cell Reports Medicine *6*, 102127
May 20, 2025 © 2025 The Author(s). Published by Elsevier Inc.
https://doi.org/10.1016/j.xcrm.2025.102127

CellPress

Cell Reports Medicine

CellPress OPEN ACCESS

Article

Dysfunctional one-carbon metabolism identifies vitamins B_6, B_9, B_{12}, and choline as neuroprotective in glaucoma

James R. Tribble,[1,8,*] Vickie H.Y. Wong,[2] Kelsey V. Stuart,[3] Glyn Chidlow,[4] Alan Nicol,[1] Anne Rombaut,[1] Alessandro Rabiolo,[5,6] Anh Hoang,[2] Pei Ying Lee,[2] Carola Rutigliani,[1] Tim J. Enz,[1,7] Alessio Canovai,[1] Emma Lardner,[1] Gustav Stålhammar,[1] Christine T.O. Nguyen,[2] David F. Garway-Heath,[3] Robert J. Casson,[4] Anthony P. Khawaja,[3] Bang V. Bui,[2] and Pete A. Williams[1,8,9,*]

[1]Department of Clinical Neuroscience, Division of Eye and Vision, St. Erik Eye Hospital, Karolinska Institutet, Stockholm, Sweden
[2]Department of Optometry and Vision Sciences, University of Melbourne, Parkville, VIC, Australia
[3]NIHR Biomedical Research Centre, Moorfields Eye Hospital NHS Foundation Trust and UCL Institute of Ophthalmology, London, UK
[4]Discipline of Ophthalmology & Visual Sciences, Level 7 Adelaide Health and Medical Sciences Building, University of Adelaide, North Terrace, Adelaide, SA 5000, Australia
[5]Department of Ophthalmology, University Hospital Maggiore della Carita', Novara, Italy
[6]Department of Health Sciences, Università del Piemonte Orientale "A.Avogadro", Novara, Italy
[7]Department of Ophthalmology, University of Basel, Basel, Switzerland
[8]Senior author
[9]Lead contact
*Correspondence: james.tribble@ki.se (J.R.T.), pete.williams@ki.se (P.A.W.)

https://doi.org/10.1016/j.xcrm.2025.102127

SUMMARY

Glaucoma, characterized by the loss of retinal ganglion cells (RGCs), is a leading cause of blindness for which there are no neuroprotective therapies. To explore observations of elevated homocysteine in glaucoma, we elevate vitreous homocysteine, which increases RGC death by 6% following ocular hypertension. Genetic association with higher homocysteine does not affect glaucoma-associated outcomes from the UK Biobank and serum homocysteine levels have no effect on glaucomatous visual field progression. This supports a hypothesis in which elevated homocysteine is a pathogenic, rather than causative, feature of glaucoma. Further exploration of homocysteine metabolism in glaucoma animal models demonstrates early and sustained dysregulation of genes involved in one-carbon metabolism and the interaction of essential cofactors and precursors (B_6, B_9, B_{12}, and choline) in whole retina and optic nerve head and RGCs. Supplementing these provides neuroprotection in an acute model and prevents neurodegeneration and protects visual function in a chronic model of glaucoma.

INTRODUCTION

Glaucoma is characterized by the dysfunction and death of retinal ganglion cells (RGCs), the output neurons of the retina. It is a common neurodegenerative disease and is the leading cause of irreversible blindness worldwide. Over 80 million people are estimated to have been diagnosed with glaucoma,[1] making it a health and economic priority. There are currently no neuroprotective therapies, and reduction of the intraocular pressure (IOP, a major, but not fully causative, risk factor) is the only proven strategy. However, some patients develop glaucoma with IOP in the normal range and continue to lose vision despite IOP-lowering, while the pursuit of "target" IOP (the IOP at which progression is deemed unlikely) can carry significant risks and negatively impact quality of life. Numerous pathogenic mechanisms have been identified in glaucoma, highlighting the complexity of the disease and the difficulty in identifying viable neuroprotective therapies.[2] Recent work has identified metabolic dysfunction in the retina and optic nerve occurring prior to detectable neurodegeneration of RGCs in glaucoma (via transcriptomic and metabolomic analyses), presenting the potential for neuroprotection prior to the initiation of neurodegenerative cascades. Loss of mitochondrial function and transport in RGCs,[3,4] reduced energy capacity,[5] loss of the ability to maintain nicotinamide adenine dinucleotide,[6,7] and the depletion of alternative energy sources[8,9] have all been identified early in glaucoma in human patients and animal models. While metabolic dysfunction has emerged as a clear pathophysiological mechanism in glaucoma, little is known about the ability to maintain anabolic function in glaucoma (e.g., one-carbon metabolism) and the potential dysregulation of metabolites that are not directly involved in energy metabolism (i.e., ATP generation).

We previously identified dysregulation of numerous small-molecular-weight metabolites and amino acids occurring with high IOP prior to detectable neurodegeneration in a rat model of glaucoma.[7] We identified elevated retinal homocysteine as the

© 2025 The Author(s). Published by Elsevier Inc.
This is an open access article under the CC BY license (http://creativecommons.org/licenses/by/4.0/).

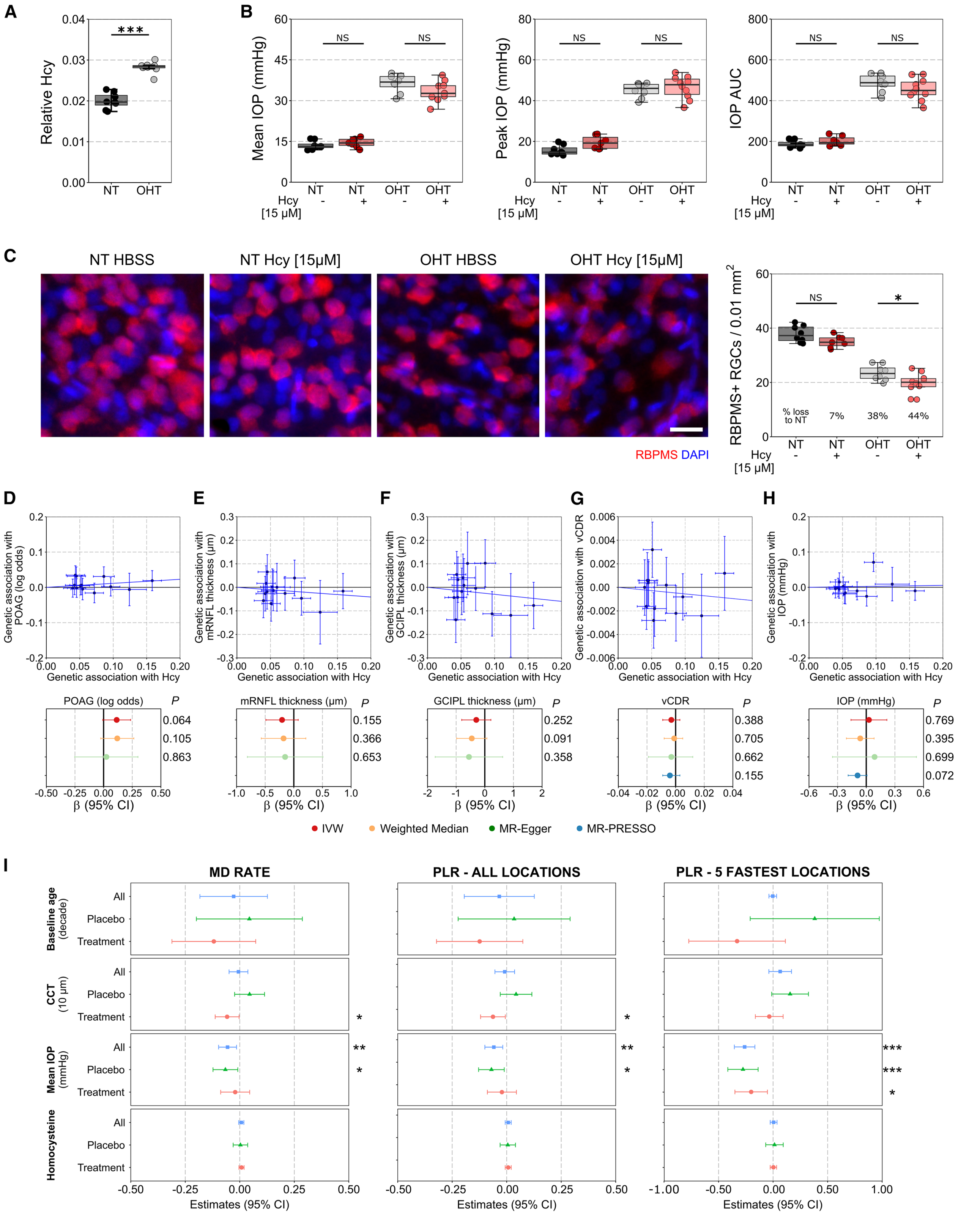

(legend on next page)

strongest early metabolomic signature.[7] Homocysteine is a non-coding amino acid with a central role in one-carbon metabolism, which maintains a number of homeostatic cellular functions. Elevated homocysteine is associated with cardiovascular disease, diabetes, and Alzheimer's disease, although this is typically in the form of hyperhomocysteinemia, where homocysteine is elevated in the blood.[10] A number of small-scale human studies suggest that homocysteine may be elevated in the blood and aqueous humor (AqH) of certain glaucoma subtypes.[11] Mouse models of hyperhomocysteinemia present significant retinal degenerative phenotypes, including vascular compromise and RGC degeneration.[12–14] Supporting this, intravitreal injection of homocysteine in high concentrations can induce RGC death.[15,16] We hypothesize that elevated homocysteine directly compounds RGC death in glaucoma and is related to dysfunction in one-carbon metabolism. To determine this, we investigated the role of homocysteine and one-carbon metabolism and their contribution to neurodegeneration across mouse and rat models of ocular hypertension (OHT) and human transcriptomic, clinical, and large-scale epidemiological data. We identify that one-carbon metabolism is disrupted in glaucoma and that treatment with one-carbon metabolism cofactors and precursors (vitamins B_6, B_9, B_{12}, and choline) is neuroprotective (in an IOP-independent manner) in two models of glaucoma.

RESULTS

Elevated homocysteine has a mild effect on susceptibility to loss of RGCs in experimental glaucoma

Hyperhomocysteinemia results in a severe retinal degenerative phenotype.[12–14] Supporting that elevated homocysteine might have a role in glaucoma, we have previously identified (through metabolomics) a significant increase in homocysteine in the retina occurring early in a rat model of OHT[7] (replotted in Figure 1A). Mild elevation of homocysteine in mice does not induce detectable RGC degeneration in normal eyes up to 90 days after injection.[16] Intravitreal injection of homocysteine to a final concentration of ~5 or 15 μM, comparable to the concentration identified in the AqH and serum of glaucoma patients, respectively,[11,17,18] did not induce detectable RGC degeneration (Figure S1A; to date, there are no studies that have measured homocysteine in the vitreous of glaucoma patients). Degeneration instead requires supraphysiological concentrations of ~500 μM and is worsened by more reactive homocysteine forms (e.g., homocysteine-thiolactone; Figure S1A). We raised intravitreal homocysteine to 15 μM in rats prior to induction of OHT (comparable to serum homocysteine levels in glaucoma patients[11]), representing a physiologically relevant high homocysteine concentration. Intravitreal injection of homocysteine or vehicle (Hank's balanced salt solution [HBSS]) had no effect on IOP (Figure 1B). Homocysteine alone (in the absence of OHT; NT-Hcy) also had no significant effect on RGC density compared to NT-HBSS eyes (7% loss, p = 0.060, Figure 1C, which was replicated in mice, Figure S1A). OHT-HBSS resulted in a significant loss of RGC density compared to NT-HBSS eyes (38% loss, $p < 0.001$), and this was significantly worsened in OHT-Hcy eyes relative to OHT-HBSS (additional 6% loss, p = 0.025), demonstrating that, at a high physiological level, homocysteine is sufficient to drive only a very mild worsening of RGC death in glaucoma (Figure 1C).

Given that hyperhomocysteinemia results in drastic vascular dropout in animal models, we also investigated the potential of homocysteine to compound vascular dysfunction in glaucoma. There were no significant changes in the morphology of the superficial vascular plexus in NT-Hcy eyes compared to NT-HBSS (normalized total vessel length, p = 0.879; junction density, p = 0.842; vessel endpoints, p = 0.100; average lacunarity, p = 0.970; Figure S1B), further supporting that a physiologically relevant elevation of homocysteine does not induce retinal degeneration. However, elevation of homocysteine with OHT resulted in subtle vascular dropout in OHT-Hcy eyes compared to OHT-HBSS eyes (total vessel length, p = 0.005) (Figure S1B). Elevated homocysteine during early glaucoma is, therefore, unlikely a driver of RGC degeneration itself but may subtly enhance both RGC loss and vascular compromise.

Elevated homocysteine has no effect on glaucoma-related traits in the general population or glaucoma progression in glaucoma patients

A number of studies suggest that glaucoma patients have elevated blood homocysteine[11]; however, these are limited by small sample sizes and the lack of strict/consistent inclusion

Figure 1. Elevated homocysteine does not significantly worsen glaucoma

(A) Homocysteine is ~50% higher in the retina of OHT rats at a time point where IOP is high, but there is no detectable neurodegeneration (3 days OHT; data extracted from Tribble et al.[7]; false discovery rate (FDR) < 0.001; n = 8 retina for both conditions).

(B) Rats received an intravitreal injection of HBSS or homocysteine (Hcy) 3 days prior to OHT induction. Homocysteine had no effect on IOP in NT or OHT rats over 14 days (NT-HBSS, n = 8 retina; NT-Hcy, n = 7 retina; OHT-HBSS, n = 8 retina; OHT-Hcy, n = 10 retina).

(C) Homocysteine had no effect on retinal ganglion cell (RGC) density (RBPMS+) at day 14 in NT animals. RGC density was significantly reduced in OHT-Hcy eyes relative to OHT-HBSS controls. Homocysteine induced a 6% further loss of RGCs relative to HBSS (n as for B). Also see Figures S1A and S1B.

(D–H) Fourteen SNPs associated with serum homocysteine (x axis; n = 44,147 people) were compared against the association of the same variants with (D) POAG risk (y axis, n = 216,257 people), (E) macular RNFL, (F) GCIPL thickness (both n = 31,434 people), (G) vertical cup-disc-ratio (vCDR; n = 111,724 people), and (H) IOP (n = 139,555 people) by Mendelian randomization (MR). Across all 4 MR analysis methods (inverse-variance weighted [IVW], weighted median, MR-Egger, and MR-PRESSO), increased homocysteine was not associated with a significant change in any glaucoma and retinal outcome measures ($p > 0.05$). Also see Tables S1–S4.

(I) The correlation between the rate of visual field progression and serum homocysteine levels was calculated in a secondary analysis of the UKGTS using linear mixed models. There were no significant associations of serum homocysteine levels to mean deviation (MD) rate, pointwise sensitivity for all locations (pointwise linear regression, PLR), and the PLR for the fastest 5 locations over time in the whole cohort or when split into placebo and treated groups. Other parameters are relevant to visual field effects as expected (all, n = 147 patients; placebo, n = 73; treatment, n = 74). Also see Figure S1C.

Scale bar, 20 μm in (C). *$p < 0.05$, **$p < 0.01$, ***$p < 0.001$; NS, $p > 0.05$. For (A)–(C), the center hinge represents the median with upper and lower hinges representing the first and third quartiles; whiskers represent 1.5 times the interquartile range. For (D)–(I), data are represented as mean and 95% confidence interval (CI).

criteria and outcome data. To address this, we first utilized large-scale, publicly available genome-wide association study (GWAS) data to perform a Mendelian randomization experiment to determine whether genetic variants associated with serum homocysteine affect liability to glaucoma or quantitative glaucoma-related traits (Table S1). Genetic propensity to higher serum homocysteine levels (n = 44,147 people) was not associated with primary open-angle glaucoma (POAG; n = 216,257 people; Figure 1D), macular retinal nerve fiber layer (mRNFL; RGC axons) thickness or combined ganglion cell layer and inner plexiform layer (GCIPL; RGC soma and dendrites) thickness (both n = 31,434 people; Figure 1E and 1F), vertical cup-to-disk ratio (vCDR; representing optic nerve head [ONH] integrity, n = 23,899 people; Figure 1G), or IOP (n = 139,555 people; Figure 1H). Full results are summarized in Tables S2–S4. These results suggest that variation in systemic homocysteine levels likely has little effect on retinal health or glaucoma outcome in the general population (but does not preclude local retinal changes to homocysteine as part of disease cascades). Next, to determine whether serum homocysteine levels were associated with glaucoma progression, we investigated the relationship between serum homocysteine levels and progressive visual field loss in a strictly controlled patient population. We performed a secondary analysis (linear mixed models) of the United Kingdom Glaucoma Treatment Study (UKGTS), a multicenter, randomized, triple-masked, placebo-controlled trial, which demonstrated the efficacy of latanoprost for IOP-lowering in patients newly diagnosed with open-angle glaucoma over 24 months.[19] There were no significant associations of serum homocysteine levels with visual field mean deviation (MD) rate, pointwise sensitivity for all locations (pointwise linear regression, PLR), and the PLR for the fastest 5 locations over time in the whole cohort (n = 147 patients) or when split into placebo (n = 73 patients) and treated (n = 74 patients) groups (Figure 1I). Other parameters relevant to visual field progression (IOP and central corneal thickness) did demonstrate significant effects as expected (Figure 1I). This demonstrates that, in a strictly controlled glaucoma patient cohort, the serum levels of homocysteine have no effect on the rate of glaucoma progression over 24 months. Similarly, at baseline, serum homocysteine levels demonstrated no correlation with IOP across all patients (r = 0.059, p = 0.753, Figure S1C). Taken together, these animal, population, and patient analyses support that homocysteine elevation has a limited effect on RGC degeneration relative to OHT.

Elevated homocysteine marks dysfunctional one-carbon metabolism

Homocysteine intersects the methionine cycle (*orange*, Figure 2A) and transsulfuration pathway (*green*, Figure 2A) as the precursor for regeneration of L-methionine (catalyzed by methionine synthetase, Mtr) and L-cystathionine (catalyzed by cystathionine β-synthase, Cbs), respectively, as part of one-carbon metabolism.[20] Conversion of homocysteine to L-methionine requires the addition of a methyl group, which is provided by 5-methyl-tetrahydrofolate through the folate cycle (*blue*, Figure 2A).[20] To determine whether changes in these pathways may explain elevated homocysteine, we quantified expression of 15 genes encoding methionine cycle, transsulfuration pathway, and folate cycle enzymes in the optic nerve. Of the 8 genes with adequate expression, none were significantly altered following 7 days of OHT relative to NT controls, but *Dnmt1* and *Cbs* were both significantly downregulated following 14 days of OHT (p = 0.002 and p = 0.023; Figure 2B). Immunofluorescent labeling of Cbs in the optic nerve confirmed this, with a significantly reduced mean pixel intensity following 14 days of OHT compared to NT controls (p = 0.002; Figure 2C). To determine if changes to homocysteine metabolism may occur earlier in the disease cascade, we repeated immunofluorescent labeling of Cbs and Mtr following 3 and 7 days of OHT (Figure S1D; time points that we have previously identified as pre-degeneration and pre/early-degeneration, respectively[21,22]). Cbs labeling was not significantly different at either time point across the inner retina (Figure 2D). Mtr labeling in the GCL was not significantly different at 3 days of OHT but was significantly decreased following 7 days of OHT relative to NT controls (68% loss; p = 0.048; Figure 2D) and was unchanged in other inner retinal layers. This supports that homocysteine elevation may occur in the retina and optic nerve through loss of Mtr and Cbs and that other components on these pathways may also be altered.

To determine whether elevated homocysteine in glaucoma may be an indicator of wider retinal metabolic dysfunction, we explored gene expression over the course of glaucoma progression in the DBA2/J mouse, a chronic and age-related model of OHT with well-characterized genetic and morphological stages of degeneration.[23] In the ONH, we identified that genes encoding for methionine and folate cycle enzymes are significantly increased, while transsulfuration pathway enzymes are downregulated. This occurs early in disease, at pre-degenerative time points, and proceeds through to severe disease (Figure 2E and Table S5). In the retina, we identified early downregulation of genes in the methionine cycle and dysregulation of the folate cycle (Figure 2E and Table S5). Early sustained downregulation of *Mtr* and *Mtrr* (methionine synthase reductase, reactivates Mtr-bound nonfunctional cob(II)alamin allowing its release) in the DBA/2J retina and loss of *Cbs* in the DBA/2J ONH match the loss of Mtr in the rat retina and Cbs in the rat optic nerve and may underlie early increased homocysteine as observed in our previous rat metabolomics. We identified significant dysregulation of genes (∼25%–30% of total genes) involved in one-carbon metabolism and related pathways, transsulfuration, sulfur amino acid metabolism, and L-methionine salvage cycle in the ONH (Figure 2F and Table S5). This dysregulation occurs at an early disease time point, prior to detectable neurodegeneration, and continues through moderate and severe disease (Figure 2F and Table S5). In the retina, there were many significant changes to genes in these pathways at early and severe disease time points (Figure 2F and Table S5). These whole-tissue changes were also present at the level of individual cell types at the earliest time point, prior to detectable degeneration, with dysregulation in RGCs in the retina and microglia and monocytes in the ONH (Figure 2G and Table S5). These support dysfunctional one-carbon metabolism across multiple retinal cell types as an early and sustained feature of glaucoma. To determine whether this may extend to humans, we queried single-cell RNA sequencing (RNA-seq) from POAG (and healthy control) patient-derived induced pluripotent stem cells (iPSCs). In the

CellPress
OPEN ACCESS

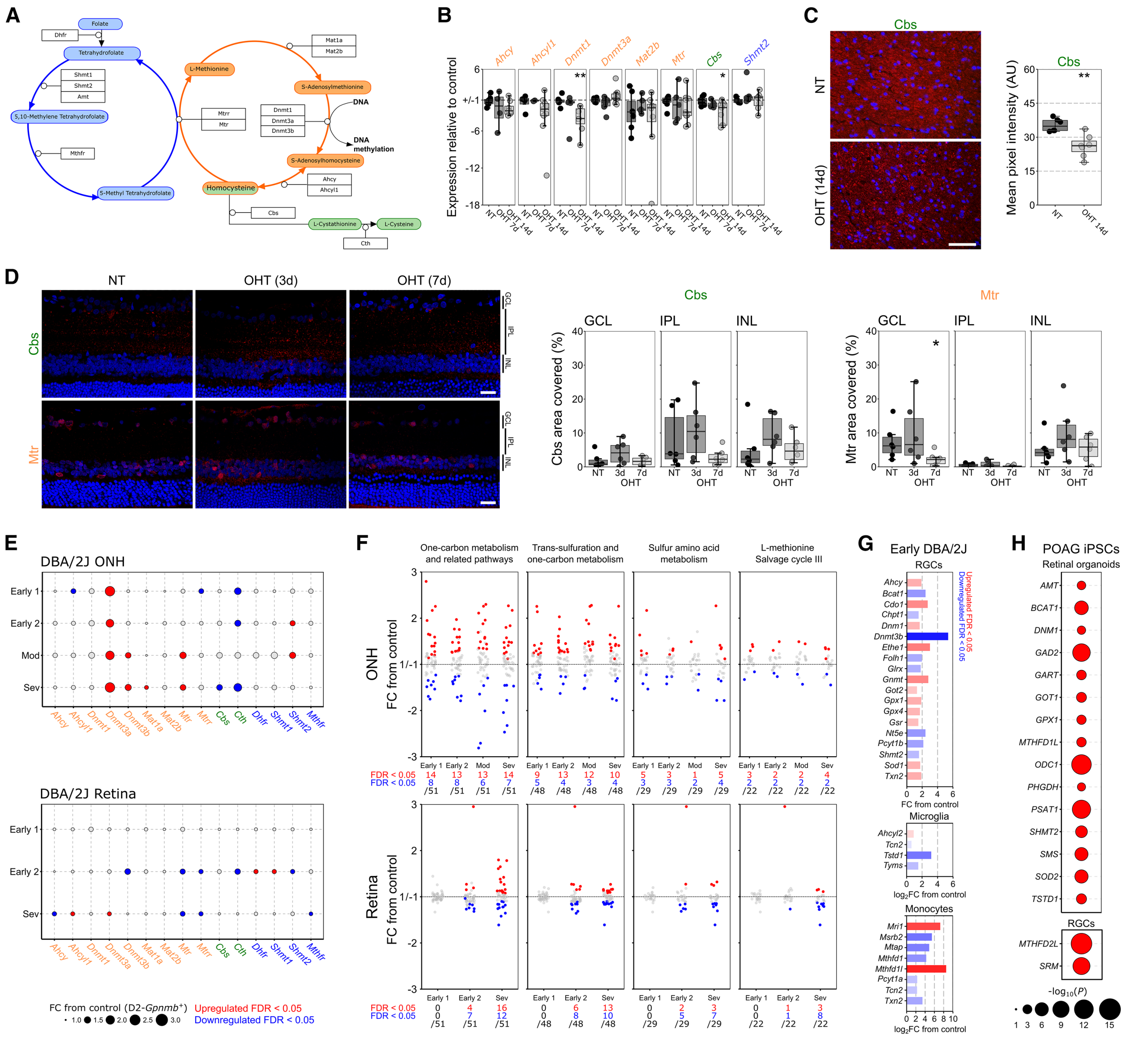

Figure 2. Dysregulation of one-carbon metabolism is an early and sustained feature of glaucoma

(A) Homocysteine is converted to methionine in the methionine cycle (*orange*) in concert with the folate cycle (*blue*) or to L-cystathionine in the transsulfuration pathway (*green*).

(B) In the rat optic nerve, ∼50% of the genes encoding the enzymes in these pathways (A) were reliably quantifiable by qPCR, of which expression of *Dnmt1* and *Cbs* was significantly reduced at 14 days post-OHT induction relative to NT control (NT, n = 8 ONs; OHT-d7, n = 6 ONs; OHT-d14, n = 9 ONs).

(C) Loss of Cbs in the optic nerve was confirmed by immunofluorescent labeling, with a reduced fluorescent intensity in the optic nerve at 14 days post-OHT induction relative to NT control (NT, n = 6 ONs; OHT-d14, n = 7 ONs).

(D) In the retina, Cbs labeling was predominantly located in the inner retina but was unchanged at 3 or 7 days post-OHT induction. Mtr labeling was significantly reduced in the GCL at 7 days post-OHT induction relative to NT control (n = 6 retina for all conditions). Also see Figure S1D.

(E) Genes in these pathways are also differentially expressed (*red*, *upregulated*; *blue*, *downregulated*) in the DBA2/J mouse model of glaucoma (relative to control, D2-*Gpnmb*+), with an overall upregulation of the methionine cycle in the ONH and downregulation in the retina from early to late disease (for ONH, Early 1 [n = 8], Early 2 [n = 6], Mod [n = 4], and Sev [n = 4] where expression is compared to n = 5 D2-Gpnmb+; in the retina, Early 1 [n = 8], Early 2 [n = 9], and Sev [n = 10]; expression is compared to n = 8 D2-Gpnmb+).

(F) Across one-carbon metabolism and related pathways as a whole, significant dysregulation of genes occurs early and is sustained to late disease (n as in E).

(G) A number of these genes are differentially expressed in RGCs (from n = 4 DBA/2J; n = 9 D2-Gpnmb+), microglia (from n = 4 DBA/2J; n = 5 D2-Gpnmb+), and infiltrating monocytes (infiltrating ONH monocytes from n = 12 DBA/2J, compared to peripheral blood monocytes from n = 8 D2-Gpnmb+) at the earliest time point.

(legend continued on next page)

RGC lineage in retinal organoids, 15 one-carbon metabolism genes were significantly altered between conditions, across pseudotime (Figure 2H and Table S5). In the final RGC clusters, expression of *SRM* and *MTHFD2L* was significantly altered (from 144 total DE genes; Figure 2H, Table S5) supporting that POAG patients may harbor intrinsic vulnerability or disposition to dysfunctional one-carbon metabolism.

The methionine cycle, folate cycle, and transsulfuration pathway require the co-enzymes methyl-cobalamin (an active form of vitamin B_{12}, for homocysteine conversion to L-methionine as a cofactor to Mtr) and pyridoxal 5′-phosphate (an active form of vitamin B_6, as a cofactor to Cbs for homocysteine conversion to L-cystathionine) (Figure 3A).[20] Conversion of homocysteine to L-methionine requires methyl donation from either 5-methyl tetrahydrofolate, generated from the precursor folate (vitamin B_9), or betaine, generated from the precursor choline.[20] Pyridoxal 5′-phosphate may also increase the activity and stability of Shmt enzymes in the folate cycle (Figure 3A; Perry et al.[24]). In the DBA2/J mouse, we identified dysregulation of genes (that encode the proteins) which interact with these cofactors and precursors at the intersection of the methionine cycle, folate cycle, and transsulfuration pathway (Figure 3B and Table S5). We hypothesized that the dysregulation of these pathways may be related to changes in the utilization, transport, and metabolism of these co-enzymes/precursors in the ONH and retina. We identified early and sustained dysregulation of genes involved in cobalamin transport and metabolism, vitamin B_6 activation to pyroxidal phosphate, metabolism of folate and pterines, and choline metabolism occurring in the ONH and retina (Figure 3C and Table S5). Supporting this, dysregulation of a number of these genes was identified in RGCs, microglia, and monocytes (Figure 3D and Table S5) and in POAG iPSC-derived retinal organoids (in the RGC lineage) and RGCs (3 and 1 DE genes, respectively; Figure 3E and Table S5). Dysfunctional one-carbon metabolism in the retina and optic nerve may therefore, in part, be explained by a local dysregulation of vitamin metabolism.

Supplementation of vitamin B_6, B_9, B_{12}, and choline provides structural and functional neuroprotection

We aimed to test whether supplementing these one-carbon metabolism cofactors and precursors (vitamin B_6, B_9, B_{12}, and choline) could provide neuroprotection to the retina and optic nerve in glaucoma. To test this, we first determined whether oral supplementation of these could reach the retina to affect changes to homocysteine and one-carbon metabolism. Mice were given B_{12} only or B_6, B_9, B_{12}, and choline in drinking water one week prior to intravitreal injection of a supraphysiological concentration of homocysteine (500 μM). Supplementation for 2 weeks had no effects on RGC density in naive mice (Figure S2A). Homocysteine injection resulted in ~10% RGC loss within 7 days compared to HBSS-injected controls ($p < 0.001$; Figure 4A). B_{12} alone ($p = 0.865$ relative to HBSS controls) and B_6, B_9, B_{12}, and choline ($p = 0.496$ relative to HBSS controls) completely preserved RGC density (Figure 4A). This was replicated with an alternate form of homocysteine, homocysteine-thiolactone (a reactive by-product of hyperhomocysteinemia), which drives enhanced RGC degeneration (Figure S2B). We next tested whether prophylactic supplementation of these vitamins could provide neuroprotection in glaucoma. There were no significant changes to IOP in OHT-treated animals compared to OHT controls (Figure 4B), supporting that any changes in outcome were IOP independent. B_{12} alone had no effect on RGC survival compared to untreated OHT controls ($p = 0.667$; Figure 4C), but the combination of B_6, B_9, B_{12}, and choline significantly increased RGC survival ($p = 0.008$ relative to OHT control; Figure 4C), although it did not completely prevent RGC death ($p < 0.001$ relative to NT control; Figure 4C). To determine whether the protection of RGC somas was accompanied by a protection of RGC axons, we quantified the abundance of markers related to optic nerve axonal integrity, stress, and inflammation (Figure 4D). OHT resulted in a loss of SMI31 (non-phosphorylated heavy and medium neurofilaments) and increase in SMI32 (hyper-phosphorylated heavy and medium neurofilaments) (Figure S2C) consistent with a decrease in healthy axons and an increase in damaged axons. Treatment with B_6, B_9, B_{12}, and choline limited SMI31 loss but did not prevent increased SMI32 (Figure S2C), suggesting that axon damage was delayed or reduced but not protected against fully. Supporting moderate but incomplete protection, markers of stress (alpha (β)-crystallin and heat shock protein 27; chaperone proteins involved in various stress responses) were increased in OHT relative to NT. Alpha(β)-crystallin, but not heat shock protein 27, increase was significantly protected against by supplementation (Figure S2C). Markers of inflammation (Cd74, major histocompatibility complex class II invariant chain and a marker of pro-inflammatory microglia, and Cd68/ED-1, a transmembrane protein detectable in pro-inflammatory macrophages/microglia) were increased in OHT relative to NT and remained largely unchanged by supplementation (Figure S2C). Unsupervised hierarchical clustering of all optic nerves based on these 6 variables demonstrated that a third of treated optic nerves clustered with NT controls (while no untreated optic nerves clustered) supporting that at a population level the neuroprotective effect was mixed, including generally unaltered neurodegenerative profiles and strongly neuroprotected profiles (Figure 4D). These results together suggest that B_6, B_9, B_{12}, and choline can provide moderate neuroprotection in an acute OHT model of glaucoma.

To determine if neuroprotection would extend to a more chronic model, OHT was induced unilaterally in mice using a circumlimbal suture model, which generates a mild IOP increase over a 7-week duration (Figure 4E). We identified no significant differences in IOP profile between untreated and B_6-, B_9-, B_{12}-, and choline-treated eyes (Figure 4E; Figures S3A–S3D), further

(H) Similarly, in iPSCs from POAG patients, a number one-carbon metabolism-related genes are differentially expressed across pseudotime in the RGC lineage from whole retinal organoids and in final RGC clusters relative to controls ($n = 54$ POAG; $n = 56$ control cell lines). Also see Table S5.
Scale bar, 50 μm in (C) and 20 μm in (D). For (B)–(D), the center hinge represents the median with upper and lower hinges representing the first and third quartiles; whiskers represent 1.5 times the interquartile range.

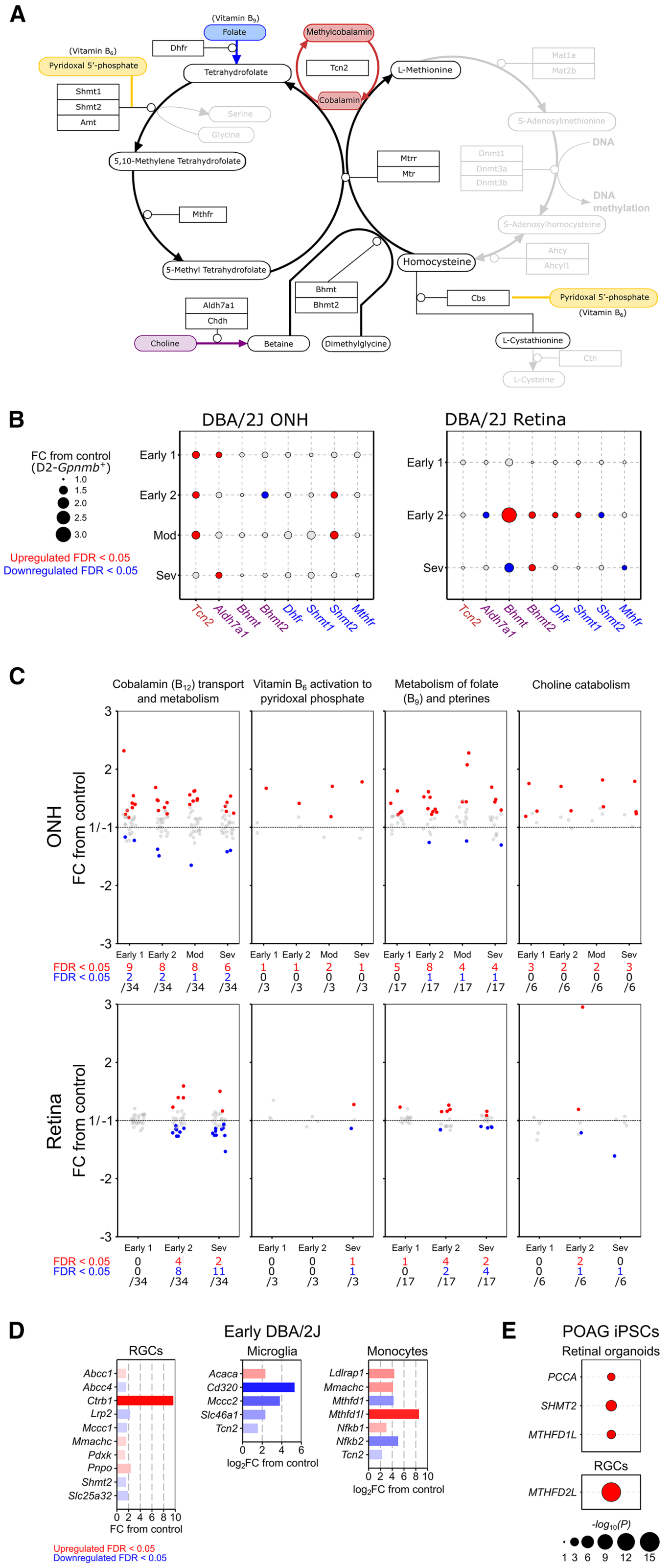

Figure 3. Dysregulation of genes related to one-carbon metabolism cofactors occurs in glaucoma

(A) The methionine cycle, folate cycle, and transsulfuration pathway require B_6 (*yellow*) and B_{12} (*red*) as cofactors, and B_9 (*blue*) and choline (*purple*) as precursors.

(B) Genes in these pathways (encoding proteins which interact with these cofactors and precursors) are differentially expressed (*red*, *upregulated*; *blue*, *downregulated*) in the DBA2/J mouse model of glaucoma (relative to control, D2-*Gpnmb*+), and (C) genes involved in the activation, transport, and metabolism of these cofactors and precursors are significantly dysregulated in the ONH and retina across disease stages (for ONH, Early 1 [n = 8], Early 2 [n = 6], Mod [n = 4], and Sev [n = 4] where expression is compared to n = 5 D2-Gpnmb+; in the retina, Early 1 [n = 8], Early 2 [n = 9], and Sev [n = 10]; expression is compared to n = 8 D2-Gpnmb+).

(D) This was also evident in RGCs, microglia, and monocytes at the earliest disease point (RGCs from n = 4 DBA/2J and n = 9 D2-Gpnmb+; microglia from n = 4 DBA/2J and n = 5 D2-Gpnmb+; infiltrating ONH monocytes from n = 12 DBA/2J; and peripheral blood monocytes from n = 8 D2-Gpnmb+).

(E) Some of these genes are also differentially expressed in iPSC-derived retinal organoids (in the RGC lineage across pseudotime) and RGCs from POAG patients relative to controls (n = 54 POAG; n = 56 control cell lines). Also see Table S5.

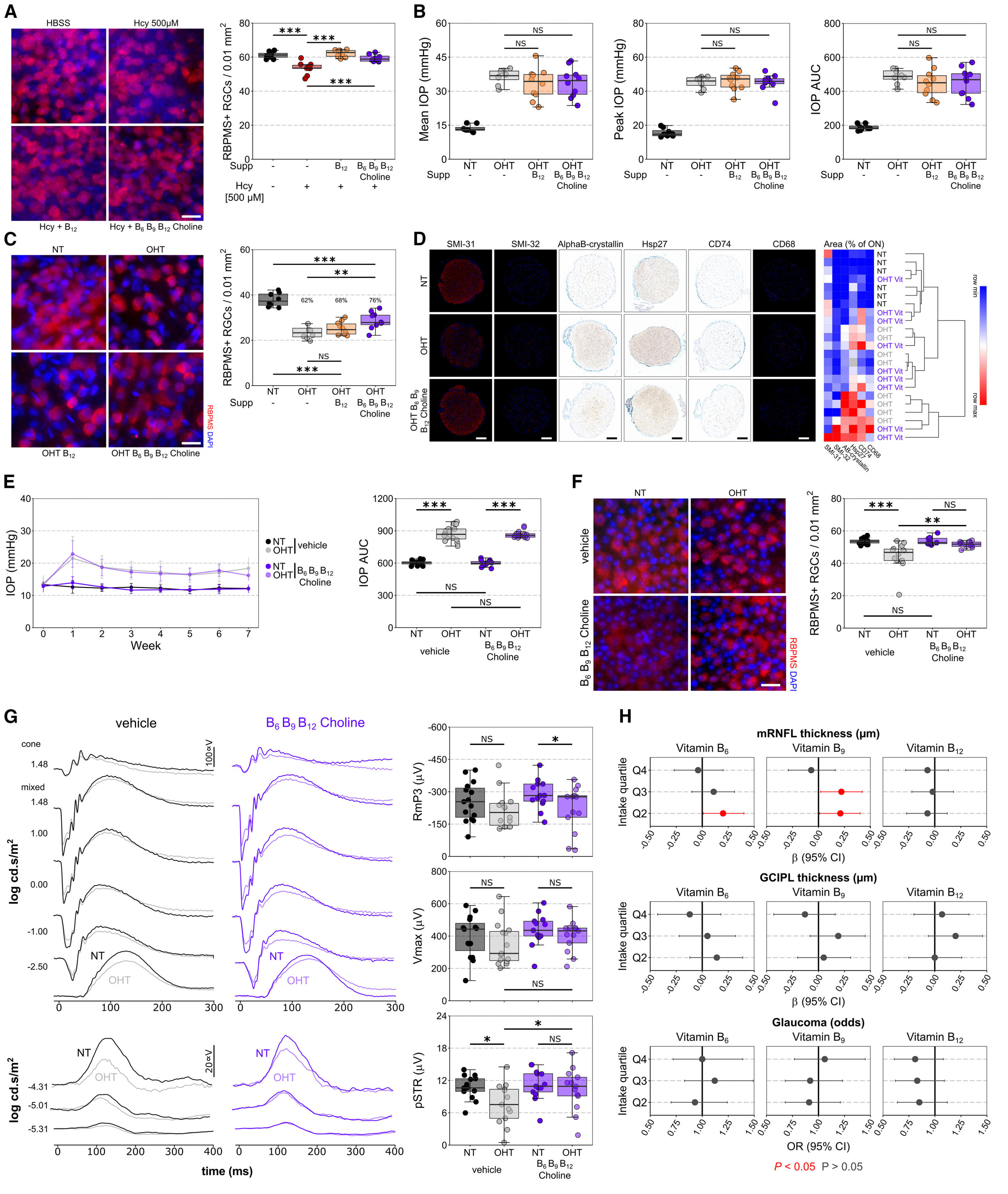

Figure 4. Supplementation of vitamin B_6, B_9, B_{12}, and choline provides structural and functional neuroprotection of retinal ganglion cells
(A) Intravitreal injection of a supraphysiological concentration of homocysteine (500 μM) causes significant RGC degeneration (loss of RBPMS+ cells) by 7 days post-injection, which is prevented by 1 week of pre-treatment with either 20 μg/kg/day vitamin B_{12} only or B_{12} with 4.5 mg/kg/day vitamin B_6, 1.5 mg/kg/day vitamin B_9, and 750 mg/kg/d choline (HBSS-vehicle *n* = 10 retina; Hcy-vehicle *n* = 10 retina; Hcy-B_{12} *n* = 8 retina; Hcy-B_6/B_9/B_{12}/choline *n* = 8 retina). Also see Figures S2A and S2B.

(legend continued on next page)

supporting an IOP-independent mechanism of action. At 7 weeks post-OHT induction, untreated mice demonstrated a significant loss of RGC density ($p < 0.001$ relative to contralateral NT control; Figures 4F and S3E), which was completely prevented by B_6, B_9, B_{12}, and choline treatment ($p = 0.695$ relative to NT-vehicle; $p = 0.004$ relative to OHT-vehicle; Figures 4F and S3E). Mice underwent clinical examination in the form of optical coherence tomography (OCT) imaging and electrophysiological recordings (electroretinogram, ERG; Figures 4G, S3F, and S3G). OCT analysis demonstrated no detectable thinning in retinal layers in untreated OHT or treated OHT eyes relative to NT controls (Figure S3F), supporting the mild neurodegenerative phenotype of the model. At week 7, there was a significant reduction in the RGC-specific positive scotopic threshold response (pSTR) amplitude in untreated OHT eyes relative to NT controls ($p = 0.008$ relative to contralateral NT control; Figures 4G and S3G) corresponding to a loss of RGC function. This occurred in the absence of changes to photoreceptor, bipolar cell, or amacrine responses (Figure S3G). B_6, B_9, B_{12}, and choline treatment prevented a significant decrease in pSTR amplitude in OHT eyes ($p = 0.043$ relative to OHT-vehicle; $p = 0.426$ relative to contralateral NT-treated; Figures 4G and S3G), thus demonstrating a functional protection of RGCs in addition to protecting against cell death.

To assess the potential human relevance of these findings we used dietary data from the UK Biobank to determine whether increased intake of vitamins B_6, B_9, and B_{12} was associated with improved glaucoma-relevant outcomes (choline intake could not be assessed from the available data). Dietary intake was quantified from participants that completed at least 2 questionnaires (24-h dietary recall, Oxford WebQ questionnaire). Multivariable linear (for mRNFL and GCIPL thickness) and logistic (for glaucoma status) regression, with adjustment for covariables (see assessment of covariables), demonstrated that intake of 1.64–1.99 mg of B_6 (Q2) or 247.2–302.5 μg (Q2) and 302.5–366.3 μg (Q3) of B_9 was associated with significantly thicker mRNFL (Figure 4H and Table S6). However, there was no significant relationship between mRNFL thickness, GCIPL thickness, or glaucoma odds with either B_6, B_9, or B_{12} per SD increase or as a p value trend across quartiles of intake (Table S6), suggesting the lack of a clear association in this dataset of 6,904 individuals.

DISCUSSION

Our study demonstrates that elevated homocysteine worsens glaucoma outcome in animal models but that this effect is mild. In humans, elevation of serum homocysteine has no detectable effect on glaucoma-related outcomes or disease progression. Early elevation of homocysteine in the retina is a marker of dysfunctional one-carbon metabolism, which we identify as an early and sustained feature of glaucoma. This is associated with dysfunctional regulation of genes and proteins that interact with key one-carbon metabolism cofactors and precursors: vitamin B_6, B_9, B_{12}, and choline. We demonstrate that supplementation of these provides neuroprotection against RGC loss in acute and mild/chronic animal models of glaucoma, including protection of visual function.

We hypothesized that early increased retinal homocysteine would contribute to RGC death, given previous findings on the toxicity of homocysteine in the retina. Intravitreal injection of homocysteine to yield acute, extreme elevation (>200 μM in the vitreous) is sufficient to induce RGC death in the absence of disease (i.e., in normal retina),[15,25] potentially through an NMDA-toxicity-like mechanism.[26] However, modest elevation (<15 μM in the vitreous) alone has no effect on RGC survival, with no changes to retinal morphology as far as 90 days post-injection. We replicated these findings in rats and mice up to our endpoint 14 days post-injection. Chronic elevation of homocysteine through knockout of *Cbs* in mice (*B6.129P2-Cbs*^{tm1Unc}*/J*) drives severe elevation of plasma (~30-fold) and retinal homocysteine (~7-fold), drastic retinal vasculopathy (vascular leakage and loss), ERG dysfunction, thinning of the inner and outer nuclear layers, and RGC loss and causes premature death (at ~3–5 weeks[12–14]). In contrast, *Cbs*$^{+/-}$ heterozygotes have milder elevation of plasma (~7-fold) and retinal homocysteine (~2-fold; comparable to the increase in the retina that we observed by metabolomics) but demonstrate no loss of RGCs or changes to retinal thickness and no changes to visual acuity, contrast sensitivity, or ERG waveforms compared to WT controls as far as

(B and C) (B) The same supplement dose had no effect on IOP over 14 days of OHT in pre-treated rats and (C) provided significant but limited neuroprotection to RGCs (B_{12} alone had no effect on RGC survival; HT-vehicle $n = 8$ retina; OHT-vehicle $n = 8$ retina; OHT-B_{12} $n = 10$ retina; OHT-B_6/B_9/B_{12}/choline $n = 10$ retina).
(D) Analysis of optic nerves with markers of RGC axon integrity, stress, and inflammation demonstrated some significant but incomplete protection, as supported by unsupervised hierarchical clustering where 33% of treated nerves clustered with normal controls rather than untreated OHT demonstrating an overall protected profile (no untreated OHT nerves have a profile like NT nerves; HT-vehicle $n = 6$ ONs; OHT-vehicle $n = 8$ ONs; OHT-B_6/B_9/B_{12}/choline $n = 9$ ONs). Also see Figure S2C.
(E and F) (E) In a milder, more chronic mouse model of unilateral OHT (circumlimbal suture), supplementation at the same dose also did not affect IOP and (F) provided complete neuroprotection of RGCs (vehicle $n = 15$ mice, paired NT and OHT retina; B_6/B_9/B_{12}/choline-treated $n = 15$ mice, paired NT and OHT retina).
(G) Electrophysiological analysis demonstrated a reduction in RGC-specific function (reduced scotopic threshold response [pSTR] amplitude; $n = 15$ mice, paired NT-vehicle [*black*] and OHT-vehicle [*gray*] retina), which was protected against in supplement-treated mice ($n = 13$ mice, paired NT-B_6/B_9/B_{12}/choline [*dark purple*] and OHT-B_6/B_9/B_{12}/choline [*light purple*] retina). Also see Figure S3.
(H) Intake of B_6, B_9, and B_{12} was quantified from 2 or more dietary recall questionnaires from the UK Biobank and compared to glaucoma-relevant outcomes. Intake of 1.64–1.99 mg of B_6 (Q2) or 247.2–302.5 μg (Q2) and 302.5–366.3 μg (Q3) of B_9 was associated with significantly thicker mRNFL ($p < 0.05$, *red*; $p > 0.05$, *dark gray*; for mRNFL, $n = 2{,}907$ people for Q1, $n = 2{,}995$ for Q2, $n = 2{,}879$ for Q3, $n = 2{,}990$ for Q4; for GCIPL, $n = 2{,}897$ people for Q1, $n = 2{,}995$ for Q2, $n = 2{,}880$ for Q3, $n = 2{,}979$ for Q4; for glaucoma odds, $n = 6{,}677$ people for Q1, $n = 6{,}803$ for Q2, $n = 6{,}697$ for Q3, $n = 7{,}014$ for Q4). Also see Table S6.
Scale bar, 20 μm in (A), (C), and (F) and 100 μm in (D). For (A)–(G), in the boxplots, the center hinge represents the median with upper and lower hinges representing the first and third quartiles; whiskers represent 1.5 times the interquartile range. For (E), error bars show standard deviation. For (H), data are represented as mean and 95% CI.

20 months of age.[27] Generating a neurodegenerative phenotype in *Cbs*$^{+/-}$ requires additional stressors such as compromising antioxidant responses through knockout of nuclear factor erythroid 2-related factor 2 (*Nrf2*). This causes NFL and IPL thinning, RGC loss, and reactive gliosis compared to *Cbs*$^{+/-}$ only.[25] Similarly, the combination of elevated homocysteine with OHT stress leads to a worsening of RGC survival, although this effect is mild (6% increase in death, within the 7% change from Hcy alone) even in a chronic model. This supports that, although homocysteine elevation may confer an increased susceptibility to RGC death, it is unlikely to be a major driving mechanism of disease. We elevated homocysteine only in the vitreous in order to exaggerate the elevation that we identified in the retina in OHT. *Cbs*$^{+/-}$ mice have elevated homocysteine in the blood and retina, and it is unclear whether pathology in the retina is driven internally or systemically through vasculopathy. We did identify minor vascular dropout in OHT combined with elevated homocysteine (Figure S1B), consistent with a sensitivity for homocysteine-induced vascular compromise. An important consideration is that OHT in the rat model is higher than that typically observed in humans, which may affect the magnitude of increased RGC death and vasculopathy.

A previous meta-analysis has demonstrated that plasma homocysteine is ~20% higher in POAG patients (n = 546) compared to controls (n = 535; incorporating 12 studies), although there was significant between-study heterogeneity, likely owing to the small sample size of most studies (ranging from 18 to 173 patients per arm[11]). Plasma homocysteine is higher in pseudoexfoliation glaucoma,[28] but not normal tension glaucoma.[29] MTHFR C677T (rs1801133) is the SNP with the single strongest association with plasma homocysteine levels and is associated with Alzheimer's disease.[30] For this reason, it has been consistently screened in glaucoma patients across many studies. Meta-analyses have demonstrated no association of this SNP (highlighted *green* in Table S3) with POAG in Caucasian populations,[11,31] but there may be a stronger association in Asian populations.[31] MTHFR C677T has not emerged as an associated SNP in glaucoma GWAS.[32] When considering SNPs known to be associated with serum homocysteine, we identified no significant association with glaucoma-relevant outcomes in the general population in sample sizes exceeding 200,000 individuals. Similarly, a retrospective cross-sectional analysis of serum homocysteine levels and IOP in glaucoma patients identified no relationship,[33] which we were able to replicate within the UKGTS cohort (Figure S1C). Further confirming that elevation of homocysteine does not lead to worsening of glaucoma, we demonstrated that serum homocysteine had no association with visual field outcomes over 24 months in the UKGTS, one of the most well-controlled and strictly phenotyped glaucoma populations to date. Our human data therefore support that, while homocysteine may be elevated in glaucoma patients, it has no significant or clinically relevant impact on glaucoma progression in a predominantly POAG population.

Homocysteine intersects the methionine cycle and transsulfuration pathway as the precursor for regeneration of L-methionine and generation of L-cysteine. These pathways are important components of one-carbon metabolism, which generates one-carbon units (typically methyl groups) for a variety of metabolic reactions.[20] We identified significant dysregulation of genes encoding for proteins in one-carbon metabolism and related pathways, occurring early in glaucomatous retina and ONH, prior to detectable degeneration, and continuing through to severe disease. Antibody labeling in the rat retina and optic nerve supports a reduction of notable one-carbon metabolism proteins in glaucoma. These changes were consistent with an elevation of homocysteine in the retina (i.e., downregulation and reduced labeling of *Mtr*, limiting homocysteine conversion to methionine). The methionine and folate cycle were downregulated in the retina and upregulated in the ONH. These regional differences may reflect the predominating transcriptional responses to OHT of inflammation at the ONH and RGC dysfunction and degeneration in the retina. The ONH is a site of concentrated pro-inflammation, exacerbated by monocyte infiltration.[34] Increased generation of S-adenosylmethionine (SAM) through the methionine cycle is a key driver of interleukin-1β expression and pro-inflammatory phenotypes in monocytes,[35] and increased methionine cycle activity enhances neuroinflammation.[36] One-carbon metabolism is an important provider of nucleotides, lipids, and proteins, which are required by immune cells for the rapid production of a new proteome when transitioning to pro-inflammatory states. SAM is the universal methyl donor, which is consumed to form S-adenosylhomocysteine, before being recycled to SAM through homocysteine and methionine. SAM is required for the epigenetic regulation of DNA through DNA methylation as a substrate to DNA methyltransferases (DNMT1, DNMT3A, and DNMT3B). In the retina, downregulation of the methionine cycle and increase in SAM consumers[37] (e.g., GNMT, which is also increased in our data [Figure 2G]) could have important implications for gene regulation and support a potential mechanism for a loss of transcriptional control in glaucoma. DNA hypomethylation affects neuronal survival[38,39] and synaptic function.[40] We identify the early loss of *Dnmt3b* in whole retina and in RGCs; while this is lowly abundant and typically associated with *de novo* regulation of DNA methylation, its conditional knockout in hippocampal neurons of adult mice causes impaired memory.[41] One-carbon metabolism also provides substrates for DNA damage repair, as well as ROS defense. Homocysteine can exit the methionine cycle through the transsulfuration pathway, an important up-stream source of cysteine as a component of glutathione.[42] The downregulation of the transsulfuration pathway in the ONH and optic nerve (e.g., the consistent loss of Cbs we observed in both rat and mouse models) could lead to a loss of glutathione and ROS defense against the oxidative stress that occurs in glaucoma,[43–45] and contribute to varying glutathione responses in human astrocytes from glaucoma patients.[46] In Alzheimer's disease, the TOMM40′650 SNP is associated with increased homocysteine generation, reduced glutathione, and reduced oxidative DNA damage repair.[47]

We identified that dysfunction in one-carbon metabolism occurred in concert with the dysregulation of genes controlling the import, utilization, and metabolism of key vitamin cofactors and precursors of one-carbon metabolism. Cobalamin (the active form of B_{12}) is bound to transcobalamin 2 (*Tcn2*) and complexes with low-density lipoprotein receptor-related protein 2 (*Lrp2*), cubilin (*Cubn*), or CD320 to enter cells.[48] We identified a downregulation of these in RGCs, microglia, and monocytes.

Downregulation of CD320 and B_{12} deficiency worsen anti-inflammatory responses in experimental autoimmune encephalomyelitis,[48] and B_{12} deficiency is strongly linked to various peripheral and optic neuropathies and neurodegenerative diseases.[49–52] Folate deficiency is also associated with neurodegenerative diseases.[53] We also identified a loss of folate (B_9) transport, with downregulation of the proton-coupled folate transporter (*Slc46a1*) in microglia, and the mitochondrial folate transporter (*Slc25a32*) in RGCs. In rodent and POAG iPSC RGCs, this is accompanied by a loss of transcripts encoding folate metabolism enzymes in mitochondria (e.g., downregulation of *SHMT2* and *MTHFD2L*) supporting a reduced capacity for mitochondrial folate-mediated one-carbon metabolism. This has an important role in mitochondrial purine biosynthesis, the prevention of mtDNA mutations, and methyl donor provision for mitochondrial transcription,[54] which may be linked to the observation of these features in RGC mitochondria in glaucoma[55] and contribute to the general vulnerability of mitochondria to degeneration. Increasing available B_6, B_9, B_{12}, and choline provided a significant but limited neuroprotection in an acute model of glaucoma and complete protection of RGC somas and electrophysiological function in a mild, more chronic model. Through a simple drug-gene-interaction screen, we previously identified B_9 as a potential neuroprotective candidate and demonstrated that it reduced RGC death from *ex vivo* axotomy.[56] B_6 has been shown to reduce RGC death from ischemia[57]; B_{12} and B_6 reduced hippocampal apoptosis following experimental pneumococcal meningitis[58,59]; and B_6, B_9, B_{12}, and choline in combination reduce Tau hyperphosphorylation and improve memory following hypoxic injury in mice.[60] While we demonstrate that this combination of B_6, B_9, B_{12}, and choline is neuroprotective, we have yet to experimentally determine whether this neuroprotection occurs through preventing one-carbon metabolism dysregulation.

Given the wealth of human safety data available for these vitamins, they are prime candidates to translate to the clinic. The doses used in our animal experiments have a human equivalent dose of 0.72 mg/kg B_6, 0.24 mg/kg B_9, 3.2 μg/kg B_{12}, and 80 mg/kg choline (considering body weight and surface area; see Nair et al.[61]). This is below the tolerable upper intake level (UL) for B_6 and B_{12} but exceeds this level for choline (1.37x UL) and B_9 (14.4x UL). However, the UL for B_9 is set against the risk of high folate masking B_{12} deficiency and the subsequent risk of pernicious anemia and neuropathy. In individuals where B_{12} is also supplemented or where B_{12} levels can be checked routinely, exceeding the UL of B_9 could be safe but must be balanced against the risk of excess unmetabolized folic acid in the blood, which has been associated with increased cognitive decline.[62] To explore the translational potential of B_6, B_9, B_{12}, and choline, we assessed the relationship between intake of these vitamins and glaucoma-related outcomes, but we identified no clear associations within a population of ~7,000 individuals. Similarly, a screen assessing intake of various vitamins with self-reported glaucoma in a population of ~5,000 individuals reported similar weak trends associating vitamin B_6 and B_{12} with reduced glaucoma incidence.[63] Larger sample sizes will be needed to identify meaningful population-level effects with more thorough dietary data. Testing of B_6, B_9, B_{12}, and choline within the UL limits, in a clinical trial setting for glaucoma, will be necessary to fully determine their utility for glaucoma patients. Encouragingly, in animal models, this was achieved independently of IOP, supporting the potential for additive protection when combined with existing IOP-lowering strategies. Various combinations of B_6, B_9, B_{12}, and choline have been demonstrated to improve cognitive function in Alzheimer's disease and elderly populations[64–67] and reduce the risk of age-related macular degeneration.[68]

In conclusion, we demonstrate that homocysteine does not contribute to the progression of glaucoma. Rather, elevation of homocysteine in the retina marks dysfunction of one-carbon metabolism and the interaction of its vitamin cofactors and precursors B_6, B_9, B_{12}, and choline. Supplementation of these provides robust neuroprotection with strong potential for translation to glaucoma patients.

Limitations of the study

While we identified no significant impact of high homocysteine on glaucoma progression, further studies in large cohorts of patients may be needed. These will require well-defined inclusion criteria, with models that compensate for other factors that may drive homocysteine dysregulation (e.g., cardiovascular disease and diabetes). We primarily focused on POAG, and as such we cannot conclude that homocysteine has similar effects in other glaucoma subtypes. While we demonstrate that this combination of B_6, B_9, B_{12}, and choline is neuroprotective, we have yet to experimentally determine whether this neuroprotection occurs through preventing one-carbon metabolism dysregulation. To definitively test this, RGC-specific knockouts of these key transporters will be required, likely multiple in unison, with metabolomic assessment of treated and untreated retina and optic nerves. Similarly, larger sample sizes with more comprehensive dietary data are needed to determine the relationship between intake of these vitamins and retinal health. Testing of B_6, B_9, B_{12}, and choline in a clinical trial setting for glaucoma will be necessary to fully determine their utility for preventing neurodegeneration and maintaining visual function in glaucoma patients.

RESOURCE AVAILABILITY

Lead contact

Requests for further information and resources and reagents should be directed to and will be fulfilled by the lead contact, Pete A. Williams (pete.williams@ki.se).

Materials availability

This study did not generate new unique reagents.

Data and code availability

(1) This paper analyzes existing, publicly available data, accessible at https://doi.org/10.1016/j.redox.2021.101988 (Homocysteine metabolomics in rat retina), GEO: GSE26299 (RNA microarray data from DBA/2J mice), GEO: GSE90654 (RNA-seq of RGCs from DBA/2J mice), https://doi.org/10.1186/s13041-020-00603-7 (RNA-seq of microglia from DBA/2J mice), https://doi.org/10.1186/s13024-018-0303-3 (RNA-seq of monocytes from DBA/2J mice), and https://doi.org/10.1016/j.xgen.2022.100142 (RNA-seq of iPSC RGCs from POAG patients). The UK Biobank data reported in this study cannot be deposited in a public repository because they are accessible only through the UK Biobank Research Analysis Platform. To request access, see the UK Biobank

online Access Management System. Summary statistics describing these data/processed datasets derived from these data are available in the supplemental information. All other data reported in this paper will be shared by the lead contact upon request.
(2) This paper does not report original code.
(3) Any additional information required to reanalyze the data reported in this paper is available from the lead contact upon request.

ACKNOWLEDGMENTS

The authors would like to thank St. Erik Eye Hospital for financial support for research space and facilities. We also thank the full list of UKGTS investigators for their role in the trial and associated data.

J.R.T. is supported by Jeanssons Stiftelser (J2021-0041), Petrus & Augusta Hedlunds Stiftelse, Ögonfonden, Loo and Hans Osterman Foundation for Medical Research, Stiftelsen Kronprinsessan Margaretas Arbetsnämnd för synskadade, Åke Wibergs Stiftelse, KI Eye Disease Research Foundation, KID-funding, and St. Erik Eye Hospital philanthropic donations.

K.V.S. is supported by Fight for Sight (London) and the Desmond Foundation.

The principal funding for the UKGTS was through an unrestricted investigator-initiated research grant from Pfizer, with supplementary funding from the UK's NIHR Biomedical Research Centre at Moorfields Eye Hospital NHS Foundation Trust and UCL Institute of Ophthalmology, London, UK. D.F. G.-H.'s chair at UCL is supported by funding from Glaucoma UK.

A.P.K. is supported by a UK Research and Innovation Future Leaders Fellowship, an Alcon Research Institute Young Investigator Award, and a Lister Institute of Preventive Medicine Award. This research was supported by the NIHR Biomedical Research Centre at Moorfields Eye Hospital and the UCL Institute of Ophthalmology.

P.A.W. is supported by Karolinska Institutet in the form of a Board of Research Faculty Funded Career Position, St. Erik Eye Hospital philanthropic donations, and Vetenskapsrådet (2018-02124 and 2022-00799).

AUTHOR CONTRIBUTIONS

J.R.T. performed experiments, performed analysis, created data visualization, wrote the manuscript, provided resources, provided supervision, and conceptualized ideas/experiments/methodologies; V.H.Y.W. and K.V.S. and G.C. performed experiments, performed analysis, and wrote the manuscript; A.N. performed analysis; A. Rombaut and A. Rabiolo and C.R. performed experiments and performed analysis; T.J.E. performed analysis; A.H. and P.Y.L. performed experiments and performed analysis; A.C. and E.L. performed experiments; G.S. provided resources; C.T.O.N. performed experiments and performed analysis; D.F.G.-H. provided data from the UKGTS and contributed to data interpretation and draft manuscript revision; R.J.C. provided resources and contributed to draft manuscript revision; A.P.K. conceptualized ideas/experiments/methodologies; B.V.B. performed experiments, performed analysis, and conceptualized ideas/experiments/methodologies; P.A.W. wrote the manuscript, provided resources, provided supervision, and conceptualized ideas/experiments/methodologies.

DECLARATION OF INTERESTS

A.P.K. has acted as a paid consultant or lecturer to AbbVie, Aerie, Allergan, Google Health, Heidelberg Engineering, Novartis, Reichert, Santen, Thea, and Topcon.

STAR★METHODS

Detailed methods are provided in the online version of this paper and include the following:

- KEY RESOURCES TABLE
- EXPERIMENTAL MODEL AND STUDY PARTICIPANT DETAILS
  - Experimental animals
  - UK biobank
  - Secondary analysis of homocysteine in UKGTS
- METHOD DETAILS
  - Electroretinography (ERG)
  - Optical Coherence Tomography (OCT)
  - Histology
  - Gene expression analysis
  - Mendelian randomization analyses
  - UK biobank dietary B-vitamin analysis
- QUANTIFICATION AND STATISTICAL ANALYSIS
  - Histology analysis
  - Gene expression analysis
  - Publicly available genetic data
  - ERG analysis
  - Mendelian randomization statistical analyses
  - Dietary B-vitamin intake and glaucoma-related parameters
  - Association of homocysteine to visual field progression
- ADDITIONAL RESOURCES

SUPPLEMENTAL INFORMATION

Supplemental information can be found online at https://doi.org/10.1016/j.xcrm.2025.102127.

Received: July 9, 2024
Revised: February 3, 2025
Accepted: April 15, 2025
Published: May 8, 2025

REFERENCES

1. Tham, Y.C., Li, X., Wong, T.Y., Quigley, H.A., Aung, T., and Cheng, C.Y. (2014). Global prevalence of glaucoma and projections of glaucoma burden through 2040: a systematic review and meta-analysis. Ophthalmology *121*, 2081–2090.
2. Tribble, J.R., Hui, F., Quintero, H., El Hajji, S., Bell, K., Di Polo, A., and Williams, P.A. (2023). Neuroprotection in glaucoma: Mechanisms beyond intraocular pressure lowering. Mol. Aspects Med. *92*, 101193.
3. Tribble, J.R., Vasalauskaite, A., Redmond, T., Young, R.D., Hassan, S., Fautsch, M.P., Sengpiel, F., Williams, P.A., and Morgan, J.E. (2019). Midget retinal ganglion cell dendritic and mitochondrial degeneration is an early feature of human glaucoma. Brain Commun. *1*, fcz035.
4. Quintero, H., Shiga, Y., Belforte, N., Alarcon-Martinez, L., El Hajji, S., Villafranca-Baughman, D., Dotigny, F., and Di Polo, A. (2022). Restoration of mitochondria axonal transport by adaptor Disc1 supplementation prevents neurodegeneration and rescues visual function. Cell Rep. *40*, 111324.
5. Belforte, N., Agostinone, J., Alarcon-Martinez, L., Villafranca-Baughman, D., Dotigny, F., Cueva Vargas, J.L., and Di Polo, A. (2021). AMPK hyperactivation promotes dendrite retraction, synaptic loss, and neuronal dysfunction in glaucoma. Mol. Neurodegener. *16*, 43.
6. Williams, P.A., Harder, J.M., Foxworth, N.E., Cochran, K.E., Philip, V.M., Porciatti, V., Smithies, O., and John, S.W.M. (2017). Vitamin B-3 modulates mitochondrial vulnerability and prevents glaucoma in aged mice. Science *355*, 756–760.
7. Tribble, J.R., Otmani, A., Sun, S., Ellis, S.A., Cimaglia, G., Vohra, R., Jöe, M., Lardner, E., Venkataraman, A.P., Domínguez-Vicent, A., et al. (2021). Nicotinamide provides neuroprotection in glaucoma by protecting against mitochondrial and metabolic dysfunction. Redox Biol. *43*, 101988.
8. Harder, J.M., Guymer, C., Wood, J.P.M., Daskalaki, E., Chidlow, G., Zhang, C., Balasubramanian, R., Cardozo, B.H., Foxworth, N.E., Deering, K.E., et al. (2020). Disturbed glucose and pyruvate metabolism in glaucoma with neuroprotection by pyruvate or rapamycin. Proc. Natl. Acad. Sci. USA *117*, 33619–33627.

CellPress
OPEN ACCESS

9. Harun-Or-Rashid, M., Pappenhagen, N., Zubricky, R., Coughlin, L., Jassim, A.H., and Inman, D.M. (2020). MCT2 overexpression rescues metabolic vulnerability and protects retinal ganglion cells in two models of glaucoma. Neurobiol. Dis. *141*, 104944.
10. McCaddon, A., and Miller, J.W. (2023). Homocysteine-a retrospective and prospective appraisal. Front. Nutr. *10*, 1179807.
11. Xu, F., Zhao, X., Zeng, S.M., Li, L., Zhong, H.B., and Li, M. (2012). Homocysteine, B vitamins, methylenetetrahydrofolate reductase gene, and risk of primary open-angle glaucoma: a meta-analysis. Ophthalmology *119*, 2493–2499.
12. Ganapathy, P.S., Moister, B., Roon, P., Mysona, B.A., Duplantier, J., Dun, Y., Moister, T.K.V.E., Farley, M.J., Prasad, P.D., Liu, K., and Smith, S.B. (2009). Endogenous elevation of homocysteine induces retinal neuron death in the cystathionine-beta-synthase mutant mouse. Invest. Ophthalmol. Vis. Sci. *50*, 4460–4470.
13. Tawfik, A., Al-Shabrawey, M., Roon, P., Sonne, S., Covar, J.A., Matragoon, S., Ganapathy, P.S., Atherton, S.S., El-Remessy, A., Ganapathy, V., and Smith, S.B. (2013). Alterations of retinal vasculature in cystathionine-Beta-synthase mutant mice, a model of hyperhomocysteinemia. Invest. Ophthalmol. Vis. Sci. *54*, 939–949.
14. Yu, M., Sturgill-Short, G., Ganapathy, P., Tawfik, A., Peachey, N.S., and Smith, S.B. (2012). Age-related changes in visual function in cystathionine-beta-synthase mutant mice, a model of hyperhomocysteinemia. Exp. Eye Res. *96*, 124–131.
15. Moore, P., El-sherbeny, A., Roon, P., Schoenlein, P.V., Ganapathy, V., and Smith, S.B. (2001). Apoptotic cell death in the mouse retinal ganglion cell layer is induced in vivo by the excitatory amino acid homocysteine. Exp. Eye Res. *73*, 45–57.
16. Chang, H.H., Lin, D.P.C., Chen, Y.S., Liu, H.J., Lin, W., Tsao, Z.J., Teng, M.C., and Chen, B.Y. (2011). Intravitreal homocysteine-thiolactone injection leads to the degeneration of multiple retinal cells, including photoreceptors. Mol. Vis. *17*, 1946–1956.
17. Ghanem, A.A., Mady, S.M., El awady, H.E., and Arafa, L.F. (2012). Homocysteine and hydroxyproline levels in patients with primary open-angle glaucoma. Curr. Eye Res. *37*, 712–718.
18. Roedl, J.B., Bleich, S., Reulbach, U., von Ahsen, N., Schlötzer-Schrehardt, U., Rejdak, R., Naumann, G.O.H., Kruse, F.E., Kornhuber, J., and Jünemann, A.G.M. (2007). Homocysteine levels in aqueous humor and plasma of patients with primary open-angle glaucoma. J. Neural Transm. *114*, 445–450.
19. Garway-Heath, D.F., Crabb, D.P., Bunce, C., Lascaratos, G., Amalfitano, F., Anand, N., Azuara-Blanco, A., Bourne, R.R., Broadway, D.C., Cunliffe, I.A., et al. (2015). Latanoprost for open-angle glaucoma (UKGTS): a randomised, multicentre, placebo-controlled trial. Lancet *385*, 1295–1304.
20. Ducker, G.S., and Rabinowitz, J.D. (2017). One-Carbon Metabolism in Health and Disease. Cell Metab. *25*, 27–42.
21. Tribble, J.R., Otmani, A., Kokkali, E., Lardner, E., Morgan, J.E., and Williams, P.A. (2021). Retinal Ganglion Cell Degeneration in a Rat Magnetic Bead Model of Ocular Hypertensive Glaucoma. Transl. Vis. Sci. Technol. *10*, 21.
22. Tribble, J.R., Jöe, M., Varricchio, C., Otmani, A., Canovai, A., Habchi, B., Daskalakis, E., Chaleckis, R., Loreto, A., Gilley, J., et al. (2024). NMNAT2 is a druggable target to drive neuronal NAD production. Nat. Commun. *15*, 6256.
23. Howell, G.R., Macalinao, D.G., Sousa, G.L., Walden, M., Soto, I., Kneeland, S.C., Barbay, J.M., King, B.L., Marchant, J.K., Hibbs, M., et al. (2011). Molecular clustering identifies complement and endothelin induction as early events in a mouse model of glaucoma. J. Clin. Investig. *121*, 1429–1444.
24. Perry, C., Yu, S., Chen, J., Matharu, K.S., and Stover, P.J. (2007). Effect of vitamin B6 availability on serine hydroxymethyltransferase in MCF-7 cells. Arch. Biochem. Biophys. *462*, 21–27.
25. Navneet, S., Zhao, J., Wang, J., Mysona, B., Barwick, S., Ammal Kaidery, N., Saul, A., Kaddour-Djebbar, I., Bollag, W.B., Thomas, B., et al. (2019). Hyperhomocysteinemia-induced death of retinal ganglion cells: The role of Müller glial cells and NRF2. Redox Biol. *24*, 101199.
26. Dun, Y., Thangaraju, M., Prasad, P., Ganapathy, V., and Smith, S.B. (2007). Prevention of excitotoxicity in primary retinal ganglion cells by (+)-pentazocine, a sigma receptor-1 specific ligand. Investig. Ophthalmol. Vis. Sci. *48*, 4785–4794.
27. Xiao, H., Wang, J., Barwick, S.R., Yoon, Y., and Smith, S.B. (2022). Effect of long-term chronic hyperhomocysteinemia on retinal structure and function in the cystathionine-β-synthase mutant mouse. Exp. Eye Res. *214*, 108894.
28. Xu, F., Zhang, L., and Li, M. (2012). Plasma homocysteine, serum folic acid, serum vitamin B12, serum vitamin B6, MTHFR and risk of pseudoexfoliation glaucoma: a meta-analysis. Graefes Arch. Clin. Exp. Ophthalmol. *250*, 1067–1074.
29. Li, J., Xu, F., Zeng, R., Gong, H., and Lan, Y. (2016). Plasma Homocysteine, Serum Folic Acid, Serum Vitamin B12, Serum Vitamin B6, MTHFR, and Risk of Normal-Tension Glaucoma. J. Glaucoma *25*, e94–e98.
30. de Lau, L.M.L., van Meurs, J.B.J., Uitterlinden, A.G., Smith, A.D., Refsum, H., Johnston, C., and Breteler, M.M.B. (2010). Genetic variation in homocysteine metabolism, cognition, and white matter lesions. Neurobiol. Aging *31*, 2020–2022.
31. Yang, Y.M., Liu, Y.P., Li, D.Y., Yu, M., Gong, B., Wang, L., and Shuai, P. (2021). Association of MTHFR C677T polymorphism with primary open angle glaucoma: a Meta-analysis based on 18 case-control studies. Int. J. Ophthalmol. *14*, 896–902.
32. Gharahkhani, P., Jorgenson, E., Hysi, P., Khawaja, A.P., Pendergrass, S., Han, X., Ong, J.S., Hewitt, A.W., Segrè, A.V., Rouhana, J.M., et al. (2021). Genome-wide meta-analysis identifies 127 open-angle glaucoma loci with consistent effect across ancestries. Nat. Commun. *12*, 1258.
33. Leibovitzh, H., Cohen, E., Levi, A., Kramer, M., Shochat, T., Goldberg, E., and Krause, I. (2016). Relationship between homocysteine and intraocular pressure in men and women: A population-based study. Medicine (Baltim.) *95*, e4858.
34. Williams, P.A., Braine, C.E., Kizhatil, K., Foxworth, N.E., Tolman, N.G., Harder, J.M., Scott, R.A., Sousa, G.L., Panitch, A., Howell, G.R., and John, S.W.M. (2019). Inhibition of monocyte-like cell extravasation protects from neurodegeneration in DBA/2J glaucoma. Mol. Neurodegener. *14*, 6.
35. Yu, W., Wang, Z., Zhang, K., Chi, Z., Xu, T., Jiang, D., Chen, S., Li, W., Yang, X., Zhang, X., et al. (2019). One-Carbon Metabolism Supports S-Adenosylmethionine and Histone Methylation to Drive Inflammatory Macrophages. Mol. Cell *75*, 1147–1160.e5.
36. Alachkar, A., Agrawal, S., Baboldashtian, M., Nuseir, K., Salazar, J., and Agrawal, A. (2022). L-methionine enhances neuroinflammation and impairs neurogenesis: Implication for Alzheimer's disease. J. Neuroimmunol. *366*, 577843.
37. Obata, F., and Miura, M. (2015). Enhancing S-adenosyl-methionine catabolism extends Drosophila lifespan. Nat. Commun. *6*, 8332.
38. Hutnick, L.K., Golshani, P., Namihira, M., Xue, Z., Matynia, A., Yang, X. W., Silva, A.J., Schweizer, F.E., and Fan, G. (2009). DNA hypomethylation restricted to the murine forebrain induces cortical degeneration and impairs postnatal neuronal maturation. Hum. Mol. Genet. *18*, 2875–2888.
39. Fan, G., Beard, C., Chen, R.Z., Csankovszki, G., Sun, Y., Siniaia, M., Biniszkiewicz, D., Bates, B., Lee, P.P., Kuhn, R., et al. (2001). DNA hypomethylation perturbs the function and survival of CNS neurons in postnatal animals. J. Neurosci. *21*, 788–797.
40. Feng, J., Zhou, Y., Campbell, S.L., Le, T., Li, E., Sweatt, J.D., Silva, A.J., and Fan, G. (2010). Dnmt1 and Dnmt3a maintain DNA methylation and regulate synaptic function in adult forebrain neurons. Nat. Neurosci. *13*, 423–430.

41. Kong, Q., Yu, M., Zhang, M., Wei, C., Gu, H., Yu, S., Sun, W., Li, N., and Zhou, Y. (2020). Conditional Dnmt3b deletion in hippocampal dCA1 impairs recognition memory. Mol. Brain *13*, 42.

42. McBean, G.J. (2012). The transsulfuration pathway: a source of cysteine for glutathione in astrocytes. Amino Acids *42*, 199–205.

43. Chidlow, G., Wood, J.P.M., and Casson, R.J. (2017). Investigations into Hypoxia and Oxidative Stress at the Optic Nerve Head in a Rat Model of Glaucoma. Front. Neurosci. *11*, 478.

44. Jassim, A.H., Nsiah, N.Y., and Inman, D.M. (2022). Ocular Hypertension Results in Hypoxia within Glia and Neurons throughout the Visual Projection. Antioxidants *11*, 888.

45. Inman, D.M., Lambert, W.S., Calkins, D.J., and Horner, P.J. (2013). α--Lipoic acid antioxidant treatment limits glaucoma-related retinal ganglion cell death and dysfunction. PLoS One *8*, e65389.

46. Malone, P.E., and Hernandez, M.R. (2007). 4-Hydroxynonenal, a product of oxidative stress, leads to an antioxidant response in optic nerve head astrocytes. Exp. Eye Res. *84*, 444–454.

47. Prendecki, M., Florczak-Wyspianska, J., Kowalska, M., Ilkowski, J., Grzelak, T., Bialas, K., Wiszniewska, M., Kozubski, W., and Dorszewska, J. (2018). Biothiols and oxidative stress markers and polymorphisms of. Oncotarget *9*, 35207–35225.

48. Jonnalagadda, D., Kihara, Y., Groves, A., Ray, M., Saha, A., Ellington, C., Lee-Okada, H.C., Furihata, T., Yokomizo, T., Quadros, E.V., et al. (2023). FTY720 requires vitamin B. Cell Rep. *42*, 113545.

49. Mathew, A.R., Di Matteo, G., La Rosa, P., Barbati, S.A., Mannina, L., Moreno, S., Tata, A.M., Cavallucci, V., and Fidaleo, M. (2024). Vitamin B12 Deficiency and the Nervous System: Beyond Metabolic Decompensation-Comparing Biological Models and Gaining New Insights into Molecular and Cellular Mechanisms. Int. J. Mol. Sci. *25*, 590.

50. Roda, M., di Geronimo, N., Pellegrini, M., and Schiavi, C. (2020). Nutritional Optic Neuropathies: State of the Art and Emerging Evidences. Nutrients *12*, 2653.

51. Ata, F., Bint I Bilal, A., Javed, S., Shabir Chaudhry, H., Sharma, R., Fatima Malik, R., Choudry, H., and Bhaskaran Kartha, A. (2020). Optic neuropathy as a presenting feature of vitamin B-12 deficiency: A systematic review of literature and a case report. Ann. Med. Surg. *60*, 316–322.

52. Luthra, N.S., Marcus, A.H., Hills, N.K., and Christine, C.W. (2020). Vitamin B12 measurements across neurodegenerative disorders. J. Clin. Mov. Disord. *7*, 3.

53. Reynolds, E.H. (2014). The neurology of folic acid deficiency. Handb. Clin. Neurol. *120*, 927–943.

54. Cuthbertson, C.R., Arabzada, Z., Bankhead, A., Kyani, A., and Neamati, N. (2021). A Review of Small-Molecule Inhibitors of One-Carbon Enzymes: SHMT2 and MTHFD2 in the Spotlight. ACS Pharmacol. Transl. Sci. *4*, 624–646.

55. Gu, L., Kwong, J.M., Caprioli, J., and Piri, N. (2022). DNA and RNA oxidative damage in the retina is associated with ganglion cell mitochondria. Sci. Rep. *12*, 8705.

56. Enz, T.J., Tribble, J.R., and Williams, P.A. (2021). Comparison of Glaucoma-Relevant Transcriptomic Datasets Identifies Novel Drug Targets for Retinal Ganglion Cell Neuroprotection. J. Clin. Med. *10*, 3938.

57. Wang, X.D., Kashii, S., Zhao, L., Tonchev, A.B., Katsuki, H., Akaike, A., Honda, Y., Yamashita, J., and Yamashima, T. (2002). Vitamin B6 protects primate retinal neurons from ischemic injury. Brain Res. *940*, 36–43.

58. de Queiroz, K.B., Cavalcante-Silva, V., Lopes, F.L., Rocha, G.A., D'Almeida, V., and Coimbra, R.S. (2020). Vitamin B. J. Neuroinflammation *17*, 96.

59. Zysset-Burri, D.C., Bellac, C.L., Leib, S.L., and Wittwer, M. (2013). Vitamin B6 reduces hippocampal apoptosis in experimental pneumococcal meningitis. BMC Infect. Dis. *13*, 393.

60. Yu, L., Chen, Y., Wang, W., Xiao, Z., and Hong, Y. (2016). Multi-Vitamin B Supplementation Reverses Hypoxia-Induced Tau Hyperphosphorylation and Improves Memory Function in Adult Mice. J. Alzheimers Dis. *54*, 297–306.

61. Nair, A.B., and Jacob, S. (2016). A simple practice guide for dose conversion between animals and human. J. Basic Clin. Pharm. *7*, 27–31.

62. (1998). Institute of Medicine (US) Standing Committee on the Scientific Evaluation of Dietary Reference Intakes and its Panel on Folate OBV, and Choline. Dietary Reference Intakes for Thiamin, Riboflavin, Niacin, Vitamin B6, Folate, Vitamin B12, Pantothenic Acid, Biotin, and Choline (Washington (DC): National Academies Press (US)).

63. Hou, J., Wen, Y., Gao, S., Jiang, Z., and Tao, L. (2024). Association of dietary intake of B vitamins with glaucoma. Sci. Rep. *14*, 8539.

64. Morris, M.S., Sakakeeny, L., Jacques, P.F., Picciano, M.F., and Selhub, J. (2010). Vitamin B-6 intake is inversely related to, and the requirement is affected by, inflammation status. J. Nutr. *140*, 103–110.

65. Chen, H., Liu, S., Ge, B., Zhou, D., Li, M., Li, W., Ma, F., Liu, Z., Ji, Y., and Huang, G. (2021). Effects of Folic Acid and Vitamin B12 Supplementation on Cognitive Impairment and Inflammation in Patients with Alzheimer's Disease: A Randomized, Single-Blinded, Placebo-Controlled Trial. J. Prev. Alzheimers Dis. *8*, 249–256.

66. Poly, C., Massaro, J.M., Seshadri, S., Wolf, P.A., Cho, E., Krall, E., Jacques, P.F., and Au, R. (2011). The relation of dietary choline to cognitive performance and white-matter hyperintensity in the Framingham Offspring Cohort. Am. J. Clin. Nutr. *94*, 1584–1591.

67. Liu, L., Qiao, S., Zhuang, L., Xu, S., Chen, L., Lai, Q., and Wang, W. (2021). Choline Intake Correlates with Cognitive Performance among Elder Adults in the United States. Behav. Neurol. *2021*, 2962245.

68. Christen, W.G., Glynn, R.J., Chew, E.Y., Albert, C.M., and Manson, J.E. (2009). Folic acid, pyridoxine, and cyanocobalamin combination treatment and age-related macular degeneration in women: the Women's Antioxidant and Folic Acid Cardiovascular Study. Arch. Intern. Med. *169*, 335–341.

69. Schindelin, J., Arganda-Carreras, I., Frise, E., Kaynig, V., Longair, M., Pietzsch, T., Preibisch, S., Rueden, C., Saalfeld, S., Schmid, B., et al. (2012). Fiji: an open-source platform for biological-image analysis. Nat. Methods *9*, 676–682.

70. Kaplan, H.J., Chiang, C.-W., Chen, J., and Song, S.-K. (2010). Vitreous Volume of the Mouse Measured by Quantitative High-Resolution MRI. Investigative Ophthalmology & Visual Science *51*, 4414.

71. Zhao, D., Nguyen, C.T.O., Wong, V.H.Y., Lim, J.K.H., He, Z., Jobling, A.I., Fletcher, E.L., Chinnery, H.R., Vingrys, A.J., and Bui, B.V. (2017). Characterization of the Circumlimbal Suture Model of Chronic IOP Elevation in Mice and Assessment of Changes in Gene Expression of Stretch Sensitive Channels. Front. Neurosci. *11*, 41.

72. Lascaratos, G., Garway-Heath, D.F., Burton, R., Bunce, C., Xing, W., Crabb, D.P., Russell, R.A., and Shah, A.; United Kingdom Glaucoma Treatment Study Group (2013). The United Kingdom Glaucoma Treatment Study: a multicenter, randomized, double-masked, placebo-controlled trial: baseline characteristics. Ophthalmology *120*, 2540–2545.

73. Garway-Heath, D.F., Lascaratos, G., Bunce, C., Crabb, D.P., Russell, R. A., and Shah, A.; United Kingdom Glaucoma Treatment Study Investigators (2013). The United Kingdom Glaucoma Treatment Study: a multicenter, randomized, placebo-controlled clinical trial: design and methodology. Ophthalmology *120*, 68–76.

74. Zudaire, E., Gambardella, L., Kurcz, C., and Vermeren, S. (2011). A computational tool for quantitative analysis of vascular networks. PLoS One *6*, e27385.

75. Howell, G.R., Walton, D.O., King, B.L., Libby, R.T., and John, S.W.M. (2011). Datgan, a reusable software system for facile interrogation and visualization of complex transcription profiling data. BMC Genom. *12*, 429.

76. Stelzer, G., Rosen, N., Plaschkes, I., Zimmerman, S., Twik, M., Fishilevich, S., Stein, T.I., Nudel, R., Lieder, I., Mazor, Y., et al. (2016). The

GeneCards Suite: From Gene Data Mining to Disease Genome Sequence Analyses. Curr. Protoc. Bioinformatics *54*, 1.30.1–1.30.33.

77. Ben-Ari Fuchs, S., Lieder, I., Stelzer, G., Mazor, Y., Buzhor, E., Kaplan, S., Bogoch, Y., Plaschkes, I., Shitrit, A., Rappaport, N., et al. (2016). GeneAnalytics: An Integrative Gene Set Analysis Tool for Next Generation Sequencing, RNAseq and Microarray Data. OMICS *20*, 139–151.

78. Tribble, J.R., Harder, J.M., Williams, P.A., and John, S.W.M. (2020). Ocular hypertension suppresses homeostatic gene expression in optic nerve head microglia of DBA/2 J mice. Mol. Brain *13*, 81.

79. Daniszewski, M., Senabouth, A., Liang, H.H., Han, X., Lidgerwood, G.E., Hernández, D., Sivakumaran, P., Clarke, J.E., Lim, S.Y., Lees, J.G., et al. (2022). Retinal ganglion cell-specific genetic regulation in primary open-angle glaucoma. Cell Genom. *2*, 100142.

80. van Meurs, J.B.J., Pare, G., Schwartz, S.M., Hazra, A., Tanaka, T., Vermeulen, S.H., Cotlarciuc, I., Yuan, X., Mälarstig, A., Bandinelli, S., et al. (2013). Common genetic loci influencing plasma homocysteine concentrations and their effect on risk of coronary artery disease. Am. J. Clin. Nutr. *98*, 668–676.

81. 1000 Genomes Project Consortium; Auton, A., Brooks, L.D., Durbin, R. M., Garrison, E.P., Kang, H.M., Korbel, J.O., Marchini, J.L., McCarthy, S., McVean, G.A., and Abecasis, G.R. (2015). A global reference for human genetic variation. Nature *526*, 68–74.

82. Currant, H., Hysi, P., Fitzgerald, T.W., Gharahkhani, P., Bonnemaijer, P. W.M., Senabouth, A., Hewitt, A.W., UK Biobank Eye and Vision Consortium, International Glaucoma Genetics Consortium, Atan, D., et al. (2021). Genetic variation affects morphological retinal phenotypes extracted from UK Biobank optical coherence tomography images. PLoS Genet. *17*, e1009497.

83. Khawaja, A.P., Cooke Bailey, J.N., Wareham, N.J., Scott, R.A., Simcoe, M., Igo, R.P., Song, Y.E., Wojciechowski, R., Cheng, C.Y., Khaw, P.T., et al. (2018). Genome-wide analyses identify 68 new loci associated with intraocular pressure and improve risk prediction for primary open-angle glaucoma. Nat. Genet. *50*, 778–782.

84. Han, X., Steven, K., Qassim, A., Marshall, H.N., Bean, C., Tremeer, M., An, J., Siggs, O.M., Gharahkhani, P., Craig, J.E., et al. (2021). Automated AI labeling of optic nerve head enables insights into cross-ancestry glaucoma risk and genetic discovery in >280,000 images from UKB and CLSA. Am. J. Hum. Genet. *108*, 1204–1216.

85. Liu, B., Young, H., Crowe, F.L., Benson, V.S., Spencer, E.A., Key, T.J., Appleby, P.N., and Beral, V. (2011). Development and evaluation of the Oxford WebQ, a low-cost, web-based method for assessment of previous 24 h dietary intakes in large-scale prospective studies. Public Health Nutr. *14*, 1998–2005.

86. Perez-Cornago, A., Pollard, Z., Young, H., van Uden, M., Andrews, C., Piernas, C., Key, T.J., Mulligan, A., and Lentjes, M. (2021). Description of the updated nutrition calculation of the Oxford WebQ questionnaire and comparison with the previous version among 207,144 participants in UK Biobank. Eur. J. Nutr. *60*, 4019–4030.

87. Willett, W.C., Howe, G.R., and Kushi, L.H. (1997). Adjustment for total energy intake in epidemiologic studies. Am. J. Clin. Nutr. *65*, 1220S–1231S. discussion 9S-31S.

88. Chua, S.Y.L., Thomas, D., Allen, N., Lotery, A., Desai, P., Patel, P., Muthy, Z., Sudlow, C., Peto, T., Khaw, P.T., et al. (2019). Cohort profile: design and methods in the eye and vision consortium of UK Biobank. BMJ Open *9*, e025077.

89. Khawaja, A.P., Chua, S., Hysi, P.G., Georgoulas, S., Currant, H., Fitzgerald, T.W., Birney, E., Ko, F., Yang, Q., Reisman, C., et al. (2020). Comparison of Associations with Different Macular Inner Retinal Thickness Parameters in a Large Cohort: The UK Biobank. Ophthalmology *127*, 62–71.

90. Kim, K.E., and Park, K.H. (2018). Macular imaging by optical coherence tomography in the diagnosis and management of glaucoma. Br. J. Ophthalmol. *102*, 718–724.

91. Oddone, F., Lucenteforte, E., Michelessi, M., Rizzo, S., Donati, S., Parravano, M., and Virgili, G. (2016). Macular versus Retinal Nerve Fiber Layer Parameters for Diagnosing Manifest Glaucoma: A Systematic Review of Diagnostic Accuracy Studies. Ophthalmology *123*, 939–949.

92. Stuart, K.V., Luben, R.N., Warwick, A.N., Madjedi, K.M., Patel, P.J., Biradar, M.I., Sun, Z., Chia, M.A., Pasquale, L.R., Wiggs, J.L., et al. (2023). The Association of Alcohol Consumption with Glaucoma and Related Traits: Findings from the UK Biobank. Ophthalmol. Glaucoma *6*, 366–379.

93. Bradbury, K.E., Guo, W., Cairns, B.J., Armstrong, M.E.G., and Key, T.J. (2017). Association between physical activity and body fat percentage, with adjustment for BMI: a large cross-sectional analysis of UK Biobank. BMJ Open *7*, e011843.

94. Patel, P.J., Foster, P.J., Grossi, C.M., Keane, P.A., Ko, F., Lotery, A., Peto, T., Reisman, C.A., Strouthidis, N.G., and Yang, Q.; UK Biobank Eyes and Vision Consortium (2016). Spectral-Domain Optical Coherence Tomography Imaging in 67 321 Adults: Associations with Macular Thickness in the UK Biobank Study. Ophthalmology *123*, 829–840.

95. Bui, B.V., Edmunds, B., Cioffi, G.A., and Fortune, B. (2005). The Gradient of Retinal Functional Changes during Acute Intraocular Pressure Elevation. Investig. Ophthalmol. Vis. Sci. *46*, 202–213.

96. Lamb, T.D., and Pugh, E.N., Jr. (1992). A quantitative account of the activation steps involved in phototransduction in amphibian photoreceptors. J. Physiol. *449*, 719–758.

97. Wachtmeister, L. (1998). Oscillatory potentials in the retina: what do they reveal. Prog. Retin. Eye Res. *17*, 485–521.

98. Saszik, S.M., Robson, J.G., and Frishman, L.J. (2002). The scotopic threshold response of the dark-adapted electroretinogram of the mouse. J. Physiol. *543*, 899–916.

99. Bui, B.V., and Fortune, B. (2004). Ganglion cell contributions to the rat full-field electroretinogram. J. Physiol. *555*, 153–173.

100. Burgess, S., Butterworth, A., and Thompson, S.G. (2013). Mendelian randomization analysis with multiple genetic variants using summarized data. Genet. Epidemiol. *37*, 658–665.

101. Burgess, S., Bowden, J., Fall, T., Ingelsson, E., and Thompson, S.G. (2017). Sensitivity Analyses for Robust Causal Inference from Mendelian Randomization Analyses with Multiple Genetic Variants. Epidemiology *28*, 30–42.

102. Bowden, J., Davey Smith, G., Haycock, P.C., and Burgess, S. (2016). Consistent Estimation in Mendelian Randomization with Some Invalid Instruments Using a Weighted Median Estimator. Genet. Epidemiol. *40*, 304–314.

103. Bowden, J., Davey Smith, G., and Burgess, S. (2015). Mendelian randomization with invalid instruments: effect estimation and bias detection through Egger regression. Int. J. Epidemiol. *44*, 512–525.

104. Verbanck, M., Chen, C.Y., Neale, B., and Do, R. (2018). Detection of widespread horizontal pleiotropy in causal relationships inferred from Mendelian randomization between complex traits and diseases. Nat. Genet. *50*, 693–698.

105. Lawlor, D.A., Tilling, K., and Davey Smith, G. (2016). Triangulation in aetiological epidemiology. Int. J. Epidemiol. *45*, 1866–1886.

106. Bowden, J., Del Greco M, F., Minelli, C., Davey Smith, G., Sheehan, N.A., and Thompson, J.R. (2016). Assessing the suitability of summary data for two-sample Mendelian randomization analyses using MR-Egger regression: the role of the I2 statistic. Int. J. Epidemiol. *45*, 1961–1974.

STAR★METHODS

KEY RESOURCES TABLE

REAGENT or RESOURCE	SOURCE	IDENTIFIER
Antibodies		
Rabbit anti-RBPMS	Novusbio	Cat# NBP2-20112; RRID:AB_3075531
Isolectin GS-IB4 (from *Griffonia simplicifolia)*	Invitrogen	I21414
Mouse anti-NFL	Merck Millipore	Cat# MAB1615; RRID:AB_94285
Mouse anti-SMI31	Covance	Cat # SMI-31P-100; RRID:AB_2028812
Mouse anti-SMI32	Covance	Cat# SMI-32R-100; RRID:AB_509997
Mouse anti-ED1	AbD Serotec	Cat# MCA341GA; RRID:AB_566872
Goat anti-Cd74	Santa Cruz Biotechnology	Cat# sc-5438; RRID:AB_638241
Rabbit anti-Hsp27	Enzo Life Sciences	Cat # ADI-SPA-801; RRID:AB_10615795
Mouse anti-alphaB-crystallin	Leica Biosystems	Cat# NCL-ABCrys-512; RRID:AB_442024
Rabbit anti-CBS	Invitrogen	Cat# PA5-76031; RRID:AB_2719759
Rabbit anti-MTR	Invitrogen	Cat# PA5-114366; RRID:AB_2884823
Goat-anti Rabbit Alexa Fluor 568	Invitrogen	Cat# A11011; RRID:AB_143157
Streptavidin Alexa Fluor 488 conjugate	Invitrogen	S11223
Chemicals, peptides, and recombinant proteins		
Dynabead Epoxy M-450	Thermo Fisher Scientific	14011
L-Homocysteine	Merck	69453
L-Homocysteine thiolactone	Merck	H6503
Pyridoxine (vitamin B_6)	Merck	P5669
Folic acid (vitamin B_9)	Merck	F8758
Cyanocobalamin (vitamin B_{12})	Merck	V6629
Choline bitartrate	Merck	C1629
Deposited data		
Homocysteine metabolomics in rat retina	https://doi.org/10.1016/j.redox.2021.101988	Supplementary Dataset 3 related to manuscript
Publicly available RNA microarray data from DBA/2J mice	Datgan; http://glaucomadb.jax.org/glaucoma; also available at GSE26299	DBA/2J retina and ONH
Publicly available RNA-seq of RGCs from DBA/2J mice	https://doi.org/10.1126/science.aal0092; also available at GSE90654	GEO: GSE90654
Publicly available RNA-seq of microglia from DBA/2J mice	https://doi.org/10.1186/s13041-020-00603-7	Additional file 3 related to manuscript
Publicly available RNA-seq of monocytes from DBA/2J mice	https://doi.org/10.1186/s13024-018-0303-3	NA
Publicly available RNA-seq of iPSC RGCs from POAG patients	https://doi.org/10.1016/j.xgen.2022.100142	Tables S7 and S10 related to manuscript
Experimental models: Organisms/strains		
C57BL/6J mice	SCANBUR/Animal Resources Center (Canning Vale)	IMSR_JAX:000664
Brown Norway rats	Charles River	BN/Crl 091
Oligonucleotides		
pCR primers	Bio-rad	Table S7
Software and algorithms		
FIJI	Schindelin et al.[69]	https://imagej.nih.gov/ij/
Morpheus	https://software.broadinstitute.org/morpheus	NA
R	https://www.r-project.org/	version 4.0.2

(*Continued on next page*)

Continued

REAGENT or RESOURCE	SOURCE	IDENTIFIER
Prism	GraphPad Software	verion 9
CFX Maestro	Bio-rad	version 4.1.2433.129
HEYEX	Heidelberg Engineering	Version 2.6.3
Stata	StataCorp	version 18

EXPERIMENTAL MODEL AND STUDY PARTICIPANT DETAILS

Experimental animals

All animal breeding and experimental procedures were undertaken in accordance with the Association for Research for Vision and Ophthalmology Statement for the Use of Animals in Ophthalmic and Research. Individual study protocols for the rat bead model of ocular hypertension and mouse model of elevated ocular homocysteine were approved by Stockholm's Committee for Ethical Animal Research (10389-2018). All procedures performed on mice used for the mouse circumlimbal suture model of ocular hypertension adhered to the National Health and Medical Research Council of Australia guidelines for the use of animals in research and were approved by the Florey Animal Ethics Committee (23-004-UM). Animals were housed and fed in a 12 h light/12 h dark cycle with food and water available *ad libitum*. Male Brown Norway rats (*Rattus norvegicus*) aged 16–20 weeks were purchased from SCANBUR and housed for 1 week before beginning experiments. Male C57BL/6J mice (SCANBUR AB, Sollentuna, Sweden) for the mouse model of elevated ocular homocysteine were purchased at 10–12 weeks old and housed for 1–4 weeks before beginning experiments. For the mouse circumlimbal suture model of ocular hypertension, C57BL/6 mice were purchased at 8 weeks old from the Animal Resources Center (Canning Vale, WA, Australia). Mice were housed with free access to water and normal rodent chow (Barastoc, Melbourne, VIC, Australia) at 21°C across a 12-h light/dark cycle (on at 7 a.m., <50 lux inside the cage).

Rat bead model of ocular hypertension

We used an established paramagnetic bead model of ocular hypertension in male Brown Norway rats as previously described.[21] Rats were anesthetized with an intraperitoneal injection of ketamine (37.5 mg/kg) and medetomidine hydrochloride (1.25 mg/kg). Microbeads (Dynabead Epoxy M-450, Thermo Fisher Scientific, Waltham, MA, USA) were prepared in 1x Hank's balanced salt solution (HBSS -CaCl2 -MgCl2 -phenol red, Thermo Fisher Scientific) and 6–8 μL of bead solution was injected into the anterior chamber. Beads were distributed using a magnet to block the iridocorneal angle. Rats received either bilateral injections (OHT) or remained bilateral un-operated (naive), normotensive controls (NT). Baseline IOP was recorded by rebound tonometry (Tonolab, Icare Finland OY, Vantaa, Finland) the morning before surgery (day 0) and subsequently at day 3, 7, 9, 11, and 14 post induction of OHT. IOP was recorded as the mean of 5 repeat readings. Rats were habituated to IOP measurement the week prior to glaucoma induction so that all recordings were made in awake and unrestrained rats. To investigate the effects of elevated Homocysteine in glaucoma, 1 day prior to induction of OHT, rats received a 3 μL intravitreal injection of homocysteine (Hcy; Sigma-Aldrich, St. Louis, MO, USA) in HBSS to achieve a final concentration in the vitreous of 15 μM. As a vehicle only control, rats received 3 μL of HBSS only (n = 8 NT-HBSS eyes; n = 7 NT Hcy eyes, n = 8 OHT-HBSS eyes; n = 10 OHT-Hcy eyes). To investigate the neuroprotective potential of one carbon metabolism co-factors, rats received either 20 μg/kg/day vitamin B_{12} (cyanocobalamin) only or B_{12} with 4.5 mg/kg/day vitamin B_6 (pyridoxine), 1.5 mg/kg/day vitamin B_9 (folic acid), and 750 mg/kg/d Choline (Choline bitartrate) (all from Sigma-Aldrich). Supplements were dissolved in drinking water to achieve these doses based on the average daily consumption of water available *ad libitum*. To encourage intake, water was supplemented with 1% sucrose. Rats received supplemented water 1 week prior to the induction of OHT and continued throughout the 14 days of the experiment (n = 8 NT eyes; n = 8 OHT eyes, n = 10 OHT-B_{12} eyes; n = 10 OHT-B_6,B_9,B_{12},choline eyes).

Mouse model of elevated ocular homocysteine

We used a modified protocol adapted from.[15] A single injection to achieve a concentration of 5 μM in the vitreous has previously been demonstrated to cause no loss of RGCs or retinal thinning up to 90 days post-injections, while 200 μM in the vitreous cause significant retinal ganglion cell loss within 5–7 days post-injection and severe retinal thinning with loss of neurons across all retinal layers by 90 days post injection.[16] However, previous calculations have failed to consider the volume of the virtual chamber occupied by the lens and so these doses represent concentrations of ∼500 μM rather than 200 μM. In addition, they typically use homocysteine thiolactone (Hcy-thiolactone), a more toxic form of Hcy that is commonly generated in the blood in hyperhomocysteinemia. We therefore adapted these previous protocols to deliver a final concentration of Hcy or Hcy-thiolactone of 5 μM or 500 μM in the vitreous (1μL injection of 25 μM or 250 mM Hcy based on a vitreal volume of 5 μL[70]) and compared to HBSS only injected controls. Mice were anesthetized with an intraperitoneal injection of ketamine (37.5 mg/kg) and medetomidine hydrochloride (1.25 mg/kg) prior to injection. Mice were euthanized at day 7 post injection by cervical dislocation. To investigate the ability for one carbon metabolism co-factors to reach the retina, mice received treatment with either 20 μg/kg/day vitamin B_{12} only or B_{12} with 4.5 mg/kg/day vitamin B_6, 1.5 mg/kg/day vitamin B_9, and 750 mg/kg/d choline. Doses were achieved by determined by average cage consumption of water *ad libitum*. Mice received this supplemented water 1 week prior to injection of Hcy and continued through to the experimental

endpoint. ($n = 8$ HBSS eyes; $n = 4$ HBSS-B_{12} eyes; $n = 4$ HBSS-B_6,B_9,B_{12},choline eyes; $n = 7$ Hcy 5 μM eyes, $n = 10$ Hcy 500 μM; $n = 8$ Hcy 500 μM-B_{12} eyes, $n = 8$ Hcy 500 μM-B_6,B_9,B_{12},choline eyes; $n = 9$ Hcy-thiolactone 5 μM eyes, $n = 10$ Hcy-thiolactone 500 μM; $n = 6$ Hcy-thiolactone 500 μM-B_{12} eyes, $n = 8$ Hcy-thiolactone 500 μM-B_6,B_9,B_{12},choline eyes).

Mouse circumlimbal suture model of ocular hypertension

All procedures were undertaken under general (intraperitoneal injection of ketamine: xylazine, 80:10 mg/kg, Troy Laboratory, Glendenning, NSW, Australia) and topical corneal anesthesia (1 drop of proxymetacaine 0.5%, Alcon Laboratories, Frenchs Forest, NSW, Australia). Pupil mydriasis (tropicamide 1%, Alcon Laboratories) and corneal hydration were maintained during electroretinography (ERG) (Celluvisc, Allergan, Irvine, CA) and imaging (Systane, Novartis Pharmaceuticals, Macquarie Park, NSW, Australia). Body temperature was maintained at 37°C throughout anesthesia. At the end of *in vivo* assessment at week 8, anesthetized animals were euthanized by cervical dislocation and eyes were collected for immunohistochemistry. In total 40 mice were used for this model, including 2 unilateral OHT groups ($n = 15$ untreated mice, $n = 13$ supplement treated mice), a bilateral naive NT control group ($n = 10$ mice), and a bilateral naive NT supplement treated control group ($n = 12$ mice). Supplement treatment was 20 μg/kg/day vitamin B_{12} (cyanocobalamin), 4.5 mg/kg/day vitamin B_6 (pyridoxine), 1.5 mg/kg/day vitamin B_9 (folic acid), and 750 mg/kg/day Choline (Choline bitartrate, all Sigma) delivered in drinking water. The concentration to deliver this dose was calculated based on average water intake for the cage of mice. To encourage intake, water was supplemented with 2% sucrose in week 1 and 1% sucrose in week 2. In the OHT group, the right eye underwent circumlimbal suture procedure to elevate IOP, and the contralateral eye served as an NT control. In the OHT group, mice were anesthetized and a 10/0 nylon suture was threaded under the conjunctiva at 5 anchor points 1 mm posterior to the limbus.[71] A purse string knot was tied to create tension and a second conventional knot locked the suture in place. IOP measurements were undertaken in awake animals without corneal anesthesia using a rebound tonometer (TonoLab). IOP was measured at 2 min and 1 h after suture implantation, 3 days post-operation and then measured once a week thereafter (average of 10 readings) for the next 7 weeks between 10 a.m.–12 p.m. to minimize variability due to diurnal fluctuations. Visual function was tested by electroretinography (ERG) and retinal structure examined by Optical Coherence Tomography (OCT).

UK biobank

The UK Biobank was approved by the National Health Service North West Multicenter Research Ethics Committee (06/MRE08/65) and the National Information Governance Board for Health and Social Care. This research was conducted under application number 36741.

Secondary analysis of homocysteine in UKGTS

A secondary analysis of the United Kingdom Glaucoma Treatment Study (UKGTS) was performed. The UKGTS was a multicenter, randomized, triple-masked, placebo-controlled trial which explored the efficacy of Latanoprost, an IOP lowering medication, in preserving visual function in patients newly diagnosed with open-angle glaucoma ([19]; trial registration number ISRCTN96423140). The UKGTS complied with the Declaration of Helsinki's tenets and received approval from local institutional review boards, specifically the Moorfields and Whittington Research Ethics Committee on June 1, 2006 (ethics approval ref. 09/H0721/56). All participants provided written informed consent at enrollment. The study protocol, baseline characteristics, and outcomes of the UKGTS have been previously published.[19,72,73] The trial involved participants from 10 ophthalmology institutions across the United Kingdom, randomized in a 1:1 ratio to receive either latanoprost 0.005% or placebo eye drops every evening in both eyes for 24 months or until an endpoint was reached. Inclusion criteria were patients aged ≥18 years with newly diagnosed, treatment-naïve open-angle glaucoma, including primary open-angle and pseudoexfoliation glaucoma. Exclusion criteria included advanced glaucoma (defined by specific visual field mean deviation thresholds), mean baseline IOP ≥30 mmHg, Snellen best-corrected visual acuity (BCVA) < 6/12, and poor image quality on the Heidelberg retina tomograph. Participants underwent comprehensive assessments including IOP measurements, visual field (VF) tests, and imaging across eleven post-randomization visits over 24 months. The Humphrey Visual Field Analyzer was employed with specific settings and testing schedules. The current analysis focused on participants with available serum homocysteine measurements and ≥5 reliable visual fields (VFs), defining reliability based on false positive responses less than 15%. Of the total cohort, 147 patients (74 in the treatment arm and 73 in the placebo arm) met the criteria for this secondary analysis.

METHOD DETAILS

Electroretinography (ERG)

Mice were dark-adapted overnight before being anesthetized. Pupils were dilated (1% tropicamide, minimum of 10 min) and mice were placed on a heated platform. All procedures were conducted under dim red light settings to maintain dark adaptation. Full field ERG responses to short flashes of light were recorded using the Celeris D430 rodent ERG testing system (Diagnosys LLC, MA, USA). A gel (1% hydroxypropyl methylcellulose, Celluvisc, Allergan, North Sydney, Australia) coupled the cornea to the ERG electrodes. Recording equipment was contained in a Faraday cage to further minimize electromagnetic noise. Two sets of electrodes were used to record responses to dim (−5.3, −5.0, −4.3, −4.0, −3.5, −2.5, −2.0 log cd.s/m^2, silver-silver chloride, D430-02) and bright (−1.5, −1.0, 0, 1.0 1.48 log cd.s/m^2, silver D430-01) stimuli with a band-pass filter setting of 0.125–300 Hz (and 75–300 Hz for the oscillatory potentials). At the dimmest light levels 20 signals were averaged (<-3.5 log cd.s/m^2) with an interstimulus interval of 2

s. Five traces were averaged for −3.5 log cd.s/m^2 (interstimulus interval = 5 s) and for stimuli greater than −3.5 log cd.s/m^2 single traces were recorded with progressively longer interstimulus intervals (90 s at the brightest). Following collection of scotopic responses, a sequence of 1.48 log cd.s/m^2 flashes (16 traces) were recorded with 500 ms interval, which leverages the longer refractory time of the rod driven signals to return cone only responses.

Optical Coherence Tomography (OCT)

Following ERG recordings, animals underwent *in vivo* retinal structure imaging using spectral-domain OCT (Spectralis SD-OCT2, Heidelberg Engineering, Heidelberg, Germany). A drop of ocular lubricant gel (Systane, Alcon) was applied to optimize the tear film. Images were acquired with a volumetric scan pattern (7.6 × 6.3 × 1.9 mm) centered over the optic nerve head (ONH). Each volume scan consisted of 121 vertical B-scans (five repeats), and each B-scan was made up of 768 A-scans (3.87 μm axial, 9.8 μm lateral resolution). Scans were segmented using the automated segmentation algorithm of the manufacturer's software (HEYEX, Heidelberg Engineering), which was checked for errors. Thicknesses were averaged across an annulus from 3 to 6 mm (i.e., Early Treatment Diabetic Retinopathy Study [ETDRS] outer ring) from the center of the optic nerve, for the retinal nerve fiber layer (RNFL), ganglion cell inner plexiform layer (GCIPL), inner nuclear layer (INL), outer plexiform layer (OPL), outer nuclear layer (ONL), photoreceptor (Ph) as well as total retinal thickness (TRT).

Histology

All rodent tissue was fixed in 3.7% PFA for 2 h and stored in 0.37% PFA at 4°C until further processing.

Rodent retina flat mount histology and analysis

After fixation, retinas were dissected free and transferred to slides (HistoBond, Thermo Fisher Scientific). Retinas were isolated using a hydrophobic barrier pen (Avantor, Radnor Township, PA, USA). Retinas were permeabilized with 0.5% Triton X-100 (Avantor) in 1M PBS for 1 h at room temperature, blocked in 2% bovine serum albumin (Thermo Fisher Scientific) in PBS for 1 h at room temperature, and primary antibody cocktails were applied and maintained overnight at 4°C. Primary antibodies used were anti-RBPMS (RGC marker, Rabbit, 1 μm/mL; Novusbio # NBP2-20112, Bio-Techne, Minneapolis, MN, USA) and Isolectin GS-IB4 (IsoB4, lectin from *Griffonia simplicifolia*, 0.1 mg/mL; Invitrogen #I21414, Thermo Fisher Scientific). Retinas were then washed with 5 times for 5 min in PBS, and a secondary antibody cocktail was applied for 4 h at room temperature. Secondary antibodies used were Goat-anti Rabbit Alexa Fluor 568 (4 μg/mL, Invitrogen # A11011, Thermo Fisher Scientific) and Streptavidin Alexa Fluor 488 conjugate (4 μg/mL, Invitrogen #S11223, Thermo Fisher Scientific). Retinas were washed again 5 times for 5 min and DAPI nuclear stain (1 μg/mL in PBS) was applied for 10 min. Tissue was washed once in PBS before mounting using Fluoromount-G and glass coverslips. Images were acquired using a Leica DMi8 microscope with a CoolLED pE-300 white LED-based light source and a Leica DFC7000 T fluorescence color camera (all Leica Biosystems, Wetzlar, Germany). To assess cell survival in flat mounts from mouse and rat experiments, 6 images per retina (40× magnification) were acquired equidistantly at 0, 2, 4, 6, 8, and 10 o'clock from a superior to inferior line through the ONH at an eccentricity of ∼1000 μm. RGC density was determined by cropping images to 100 × 100 μm and the cell counter plugin in FIJI[69] was used to count RBPMS+ cells. RGC density was defined as the average between the 6 images. To assess blood vessel morphology in rats, a single image centered over the ONH was captured (5× magnification) and cropped to 2000x2000 μm. Vascular morphology (IsoB4 labeled) was analyzed using AngioTool.[74] A Vessel diameter of 8 μm, pixel threshold of 6 AU, and particle size filter of 600 were used to achieve faithful delineation of blood vessels. Total Vessel length (normalized to the retinal area to account for cuts in the tissue), Junction density, number of vessel endpoints, and average lacunarity were calculated for each retina. Vessel images were of sufficient quality for analysis in n = 5 NT-HBSS eyes; n = 6 NT-Hcy eyes, n = 8 OHT-HBSS eyes; n = 8 OHT-Hcy eyes).

Rat optic nerve histology and analysis

Following removal of the eyes, the brain was removed and the optic nerves dissected free as a pair attached at the optic chiasm. Following PFA fixation and storage (as above), left and right optic nerves were separated for processing. Orientation was maintained by the angle of cuts at the proximal and distal end (relative to the eye). Optic nerves (ONs) were embedded in paraffin wax and 4 μm thick cross sections were cut using a rotary microtome. ON sections were de-waxed and rehydrated through a conventional xylene-ethanol gradient. Antigen retrieval was achieved by microwaving the sections in 10 mM citrate buffer (pH 6.0) for 10 min at 95°C–100°C. Immunofluorescent labeling was performed as above. Primary antibodies used were NFL (1:1000; MAB1615, Merck Millipore), SMI31 (1:50000; clone SMI-32, Covance), and SMI32 (1:10000; clone SMI-32, Covance) for axonal labeling, and ED1 (1:500; clone ED1; AbDSerotec), Cd74 (1:2000; sc-5438, Santa Cruz Biotechnology), Hsp27 (1:2000; SPA-801, Enzo Life Sciences), and alphaB-crystallin (1:1000; Leica) for glial labeling. Images were acquired on a Zeiss Axio Scan.Z1 Digital Slide Scanner (Carl Zeiss). For glial marker imaging, a region of interest (ROI) of 250 × 250 μm within the center of the optic nerve was cropped for analysis (covering roughly half of the nerve area). The whole area of the optic nerve section within the bounds of the dura mater was segmented using the polygon tool in FIJI and the mean pixel intensity was quantified. A lower threshold was set for all images and the signal was binarized and FIJI particle analysis was performed (no size or circularity filters) to give the number of particles and the percentage of the ON area covered by signal. To establish a profile representing neurodegeneration and neuroinflammation, the % area covered for all optic nerves were combined as input variables for unsupervised hierarchical clustering (1 – Pearson's correlation, average linkage) using Morpheus (https://software.broadinstitute.org/morpheus). Cbs labeling in the optic nerve was assessed following the same methodology, using a primary antibody targeting CBS (1:1000; PA5-76031, ThermoFisher scientific).

Rat retinal section histology and analysis

Rats were euthanized at 3 (*n* = 6 eyes) or 7 days (*n* = 6 eyes) post OHT induction (NT eyes (*n* = 6 eyes) were monitored for the same 7 day period) and eyes were fixed by immersion in 3.7% PFA. Eyes were embedded in paraffin as previously described.[21] . Sections (3 μm thickness) were cut and deparaffinized in Bond DeWax solution (Leica Biosystems) and rehydrated through an ethanol gradient prior to antigen retrieval using Rodent Decloaker (Biocare Medical)at 100°C. Immunofluorescent labeling was performed as above. Primary antibodies used were MTR (1:150; PA5-114366, ThermoFisher scientific), and CBS (1:100; PA5-76031, ThermoFisher scientific). Images of the retina 500 μm either side of the ONH were acquired on a Zeiss LSM800-Airy (219.97 × 212.97 μm, 1724x1274 pixels) with constant parameters. The signal for MTR or CBS was thresholded using negative controls, the NFL/GCL, IPL, and INL were segmented manually, and FIJI particle analysis was performed (no size or circularity filters) to give the percentage of each layer covered by signal.

Gene expression analysis

qPCR of rat optic nerve

cDNA from a previously published cohort of rats[22] was amplified using a PrimePCR PreAmp Assay (Biorad) according to the manufacturers instructions and stored at −20°C. The resulting mix was diluted 1:10 and used to perform RT-qPCR using 1 μL of mix for input cDNA, 10 μL of SsoAdvanced Universal SYBR Green Supermix and 1 μL of the following DNA templates (Prime PCR Assay, Bio-Rad): *Ahcy*, *Ahcyl1*, *Dnmt1*, *Dnmt3a*, *Dnmt3b*, *Mtr*, *Mtrr*, *Mat1a*, *Mat2b*, *Cbs*, *Cth*, *Dhfr*, *Shmt1*, *Shmt2*, *Mthfr*, and *Gapdh* (housekeeping; all templates were species specific for *rattus norvegicus*). Also see Table S7. A MyIQ thermocycler was used with a 3 min activation and denaturation step at 95°C, followed by an amplification stage comprising 50 cycles of a 15 s denaturation at 95°C and 1 min annealing and plate read at 60°C. Analysis was performed according to the ΔΔCT method.

Analysis of publicly available DBA/2J data

Publicly available RNA microarray data from 10.5 month-old DBA/2J whole ONH and retina were accessed through Datgan.[75] Howell et al.[23] previously identified 5 molecularly distinct stages of disease in whole ONH from DBA/2J mice using hierarchical clustering (based on the degree of genetic change from DBA/2J-*Gpnmb*^{R150X} (D2-*Gpnmb*$^{+}$) controls). These stages correspond to the degree of neurodegeneration in histological optic nerve analysis. Group 1 = no detectable glaucoma, limited genetic change (*n* = 8; not plotted in Figures 2; 3); Group 2 = no detectable glaucoma, significant genetic change (*n* = 8; Early 1); Group 3 = no or early glaucoma, significant genetic change, (*n* = 6; Early 2); Group 4 = moderate glaucomatous degeneration, significant genetic change, (*n* = 4; Mod); Group 5 = severe glaucomatous degeneration, significant genetic change, (*n* = 4; Sev). Similarly, Howell et al.[23] identified 4 molecular clusters in the retina. Group 1 = no detectable glaucoma, limited genetic change (*n* = 8; Early 1); Group 2 = no detectable glaucoma, moderate genetic change (*n* = 9; Early 2); Group 3 = moderate glaucomatous degeneration, significant genetic change (*n* = 3; not considered due to low *n*); Group 4 = severe glaucomatous degeneration, significant genetic change (*n* = 10; Sev). To determine whether expression of genes belonging to one-carbon metabolism and related pathways was changed in glaucoma, we queried gene expression from the following pathways: One-carbon metabolism and related pathways, Transsulfuration and one-carbon metabolism, Sulfur amino acid metabolism, L-methionine salvage cycle III, Vitamin B12 metabolism and Cobalamin (Cbl, vitamin B12) transport and metabolism, Vitamins B6 activation to pyridoxal phosphate, Metabolism of folate and pterines, Choline catabolism (SuperPath from GeneAnalytics; ga.genecards.org[76,77]). A false discovery rate (FDR; *q*) < 0.05 was considered significant. The same pathways lists were queried against publicly available RNA-sequencing from FACS sorted bulk sequenced RGCs (*n* = 4 DBA/2J (cluster 4), *n* = 9 D2-Gpnmb+;[6]), microglia (*n* = 4 DBA/2J, *n* = 5 D2-Gpnmb+;[78]), and monocytes (infiltrating ONH monocytes from *n* = 12 DBA/2J (cluster 1), peripheral blood monocytes from *n* = 8 DBA/2J;[34]) all from 9 month-old DBA/2J mice with no detectable neurodegeneration through ON histology. An FDR (*q*) < 0.05 was considered significant.

Analysis of publicly available human RNA-seq data

Publicly available *single-cell(sc)RNA-sequencing data from Daniszewski* et al.[79] were used. We queried genes from the same pathways as above in iPSC (54 POAG and 56 control cell lines) derived retinal organoids (1,471 differentially expressed genes between POAG and controls, from RGC lineage across pseudotime) and RGCs (144 differentially expressed genes between POAG and controls in RGC clusters). A false discovery rate (FDR; *q*) < 0.05 was considered significant.

Mendelian randomization analyses

Instrumental variable selection

Single nucleotide polymorphisms (SNPs) associated with serum homocysteine (*n* = 44,147 people) were identified from a genome-wide association study (GWAS) meta-analysis in individuals of European ancestry.[80] This study identified 18 independent SNPs at genome-wide significance ($P < 5x10^{-8}$) which explain 6.0% of the variance in serum homocysteine levels (Table S1). At loci with multiple genome-wide significant SNPs, we excluded those with linkage disequilibrium $R^2 > 0.001$ and within 10,000 kb, retaining only the SNP with the lowest *p*-value, using the 1000 Genomes Project European reference population.[81] Palindromic SNPs with minor allele frequency >0.42 were excluded. Effect alleles were harmonized across exposure and outcome datasets. Full details of the SNPs included as instrumental variables (IVs) for the various outcomes are available in Table S3.

Outcome data sources

We utilized publicly available summary statistics from a large GWAS for macular retinal nerve fiber layer (mRNFL) and ganglion cell-inner plexiform layer (GCIPL) thickness (*n* = 31,434 people;[82]), as well as GWAS meta-analyses for intraocular pressure

(IOP; *n* = 139,555 people;[83]), vertical cup-disc-ratio (vCDR; *n* = 111,724 people;[84]), and primary open-angle glaucoma (POAG; *n* = 216,257 people;[32]) (Table S1).

UK biobank dietary B-vitamin analysis

Assessment of dietary B-vitamin intake

Approximately 70,000 UK Biobank participants completed a 24-h dietary assessment (Oxford WebQ questionnaire) as part of their baseline assessment (2009–2010).[85] Between 2011 and 2012, the questionnaire has been repeated in four different subsets (approximately 100,000 participants per cycle) with varying degrees of participant overlap. Overall, approximately 125,000 participants have completed at least two dietary questionnaires. Estimated nutrient intake, including dietary vitamin B_6 (mg), folate/B_9 (μg) and vitamin B_{12} (μg) have been calculated for these participants using food composition data from the United Kingdom Nutrient Databank, as described previously.[86] Choline intake could not be assessed from the available data. For this analysis, we included only participants with at least two complete questionnaires and calculated mean intake across all available timepoints, with adjustment for total energy intake (kJ).[87]

Assessment of inner retinal thickness and glaucoma case ascertainment

From 2009 to 2010, approximately 65,000 UK Biobank participants underwent SD-OCT imaging of both eyes using a Topcon 3D OCT-1000 Mark II (Topcon Corp., Tokyo, Japan) system.[88] The image handling, segmentation and quality control protocols have been described previously.[89] We assessed associations with two glaucoma-related OCT biomarkers – macular retinal nerve fiber layer (mRNFL) and GCIPL thickness – using individual-level OCT values from the macula-6 grid averaged across both eyes.[90,91] We excluded scans with an image quality score (signal strength) less than 45. Additionally, several segmentation indicators were calculated that also identified poor scan quality or segmentation failures; we excluded the poorest 20% of images for each of these indicators. From 2006 to 2010, the baseline touchscreen questionnaire administered to approximately 175 000 participants included a question on physician-diagnosed eye disorders. Participants were considered cases if they reported a diagnosis of glaucoma, or previous surgical or laser treatment for glaucoma, in either eye. We also included any participant carrying an International Classification of Diseases (ICD) code for glaucoma (ICD 9th revision: 365.* (excluding 365.0); ICD 10th revision: H40.* (excluding H40.0) and H42.*) in their linked hospital records at any point prior to, and up to 1 year after, the baseline assessment. We excluded cases who were diagnosed prior to 30 years of age, and controls who reported using ocular hypotensive medication or carrying an ICD code for glaucoma suspect (ICD 9th revision: 365.0; ICD 10th revision: H40.0).

Assessment of covariables

A range of participant characteristics, including socioeconomic, anthropometric, medical, and lifestyle factors were collected as part of the initial UK Biobank assessment and on the same day as the ophthalmic and baseline dietary assessments. These included age (years), gender (women, men), self-reported ethnicity (White, Black, Other), Townsend deprivation index (a measure of material deprivation based on an individual's residential postcode; a higher index score indicates greater relative poverty), body mass index (BMI; calculated as weight (kg) divided by height (m) squared), systolic blood pressure (SBP; mmHg; calculated as the mean of two measurements), glycated hemoglobin (HbA1c; mmol/mol), total cholesterol (mmol/L), smoking status (never, current, former), alcohol intake (g/day),[92] physical activity (metabolic equivalent of task [MET]-minutes/week; a measure of energy expenditure based on an adapted version of the validated International Physical Activity Questionnaire[93]), and spherical equivalent (SE; diopters; calculated as spherical power plus half cylindrical power, averaged across both eye[94]). Full details of these variables, including protocols, equipment, procedures and descriptive statistics are available online (https://www.ukbiobank.ac.uk).

QUANTIFICATION AND STATISTICAL ANALYSIS

Histology analysis

For all data from the rat bead model of ocular hypertension and mouse model of elevated ocular homocysteine, statistical analyses were performed in R (4.0.2). Data were tested for normality with a Shapiro–Wilk test. Normally distributed data were compared by Student's t test (one sided) or ANOVA (with Tukey's HSD). Non-normally distributed data analyzed by a Kruskal Wallis test followed Dunn's tests with Benjamini and Hochberg correction. For data from the mouse circumlimbal suture model of ocular hypertension, where each OHT eye is paired to its contralateral NT control eye, comparisons between groups were undertaken using repeated measures two-way ANOVA (groups x time) or paired t-tests (OHT vs. NT control eyes). Significance was determined as * = $p < 0.05$, ** = $p < 0.01$, ***$p < 0.001$, NS = non-significant ($p > 0.05$). For boxplots, the center hinge represents the median with upper and lower hinges representing the first and third quartiles; whiskers represent 1.5 times the interquartile range.

Gene expression analysis

qPCR of rat optic nerve

Statistical analysis was performed on ΔCT values. Data were tested for normality with a Shapiro–Wilk test. Normally distributed data were compared by Student's t test (one sided). Non-normally distributed data analyzed by a Wilcoxon signed-rank test. Significance was determined as * = $p < 0.05$, ** = $p < 0.01$, ***$p < 0.001$, NS = non-significant ($p > 0.05$). For boxplots, the center hinge represents the median with upper and lower hinges representing the first and third quartiles; whiskers represent 1.5 times the interquartile range.

Publicly available genetic data

Significance was reported as for the original study, with a false discovery rate (FDR; q) < 0.05 considered significant.

ERG analysis

Photoreceptor P3 analysis

As previously described,[95] the photoreceptoral response (P3), the first corneal electronegative a-wave of the ERG was modeled as a function of time (ms) and intensity (log cd.s/m^2) using a delayed Gaussian function (Equation 1)[96] to return amplitude (number of receptors and their outer segment length, RmP3, μV) and amplification of the phototransduction cascade (S, cd^{-1}.m^2/s^3). The delay term (td, in seconds) describes both biochemical and other recording latencies. The model was fit to the two brightest intensities (1.0 and 1.48 log cd.s/m^2), which elicit photoreceptor responses of saturated amplitude free of b-wave intrusion. Model parameters were optimized to minimize the root-mean-square (RMS) error term.

$$P3(i,t) = Rm_{P3} \cdot \left[1 - e^{-i \cdot S \cdot (t - t_d)^2}\right] \text{ for } t > t_d \qquad \text{(Equation 1)}$$

Bipolar cell P2 and amacrine cell oscillatory potential (OP) analysis

The bipolar cell (or P2) component of the ERG was quantified by first subtracting the family of P3 responses from their respective raw traces. The remnant waveform was then low pass filtered (−3 dB at 50 Hz) to remove the oscillatory potentials (OPs). OPs (band-pass, −3 dB at 50 and 280 Hz), which provides information about inner retinal inhibitory pathways involving amacrine cells[97] were analyzed to return peak amplitude and implicit time at the brightest light level (1.48 log cd.s/m^2). The bipolar cell response amplitude ($P2_{max}$, μV) and sensitivity (1/K, log cd.s/m^2) was then quantified by fitting the P2 amplitude as a function of all stimulus intensities (i, log cd.s/m^2) using a saturating hyperbolic function (Equation 2), by minimizing the root-mean-square (RMS) error term.

$$P2(i) = P2_{max} \cdot \frac{i}{i+K} \qquad \text{(Equation 2)}$$

RGC scotopic threshold response

RGC responses are known to dominate the rodent ERG waveform at light levels near absolute threshold.[98,99] The amplitude of the positive lobe of the scotopic threshold response (pSTR) was taken at −5.30 and −5.0 log cd.s/m^2.

ERG group comparisons

ERG analysis was performed using Prism 9 software (GraphPad Software Inc., San Diego, CA, USA). Since each OHT eye is paired to its contralateral NT control eye, comparisons between groups were undertaken using repeated measures two-way ANOVA (groups x time) or paired t-tests (OHT vs. NT control eyes). Significance was determined as * = $p < 0.05$, ** = $p < 0.01$, ***$p < 0.001$, NS = non-significant ($p > 0.05$). For boxplots, the center hinge represents the median with upper and lower hinges representing the first and third quartiles; whiskers represent 1.5 times the interquartile range.

Mendelian randomization statistical analyses

The main MR analyses were performed using a multiplicative random-effects inverse-variance weighted (IVW) method.[100] This method provides precise and efficient estimates but is sensitive to invalid IVs and pleiotropy.[101] We therefore conducted sensitivity analyses using three alternative MR methods: weighted median,[102] MR-Egger,[103] and MR pleiotropy residual sum and outlier (MR-PRESSO).[104] Each method makes different assumptions about the nature of pleiotropy and consistent estimates across methods strengthens causal inferences.[105] The weighted median method gives consistent estimates if the majority of IVs are valid, while the MR-Egger and MR-PRESSO methods can test and correct for directional pleiotropy. We assessed for heterogeneity with the I^2 and Cochran's Q statistics in the IVW model and with Rucker's Q′ statistic in MR-Egger regression. The I2GX statistic is an indicator of expected relative bias (or dilution) of the MR-Egger causal estimate.[106] In MR-Egger regression, a significant difference of the intercept from zero is evidence for average directional horizontal pleiotropy.[103] The MR-PRESSO global test evaluates for horizontal pleiotropy, the outlier test detects specific SNP outliers, and the distortion test evaluates whether there is a significant difference in the causal estimate before and after adjusting for outliers.[104] Full results of these tests and statistics are available in Table S4. All analyses were performed in R version 4.1.1 (https://www.R-project.org) using the *MendelianRandomization*, *TwoSampleMR*, and *MRPRESSO* packages. Significance was determined as * = $p < 0.05$, NS = non-significant ($p > 0.05$).

Dietary B-vitamin intake and glaucoma-related parameters

Mean nutrient intake was standardized and categorized into quartiles. To assess the relationship between dietary B-vitamin intake and the glaucoma-related parameters, we performed multivariable linear (for mRNFL and GCIPL) and logistic (for glaucoma status) regression, with adjustment for all covariables described in the section 6.7.3. Trends across dietary quartiles were examined by testing the median value of each group. Statistical tests were performed using Stata/MP version 18.0 (StataCorp. 2023. Stata Statistical Software: Release 18. College Station, TX: StataCorp LLC). Significance was determined as * = $p < 0.05$, NS = non-significant ($p > 0.05$).

CellPress
OPEN ACCESS

Association of homocysteine to visual field progression

A secondary analysis of the United Kingdom Glaucoma Treatment Study (UKGTS) was performed. The current analysis focused on participants with available serum homocysteine measurements and $\geq$5 reliable visual fields (VFs), defining reliability based on false positive responses less than 15%. Of the total cohort, 147 patients (74 in the treatment arm and 73 in the placebo arm) met the criteria for this secondary analysis. We investigated the correlation between the rate of VF mean deviation (MD) progression in the worst eye (i.e., 1 eye per patient) and homocysteine levels using linear mixed models. These models, which accommodate repeated measures, included random slopes and intercepts to account for varying progression rates and intra-patient correlations. The models factored homocysteine levels and other covariates potentially associated with progression rates (i.e., baseline age, central corneal thickness, mean IOP), along with their interaction with follow-up time. Interactions between covariates and follow-up time modeled the variables' effect on the progression rate. We analyzed the entire cohort as well as the treatment and placebo groups separately, reporting regression estimates along with 95% confidence intervals (95% CIs) and *p*-values. Significance was determined as * = $p < 0.05$, NS = non-significant ($p > 0.05$).

ADDITIONAL RESOURCES

This manuscript performs a secondary analysis of the United Kingdom Glaucoma Treatment Study (UKGTS): ISRCTN96423140, https://doi.org/10.1186/ISRCTN96423140.

Cell Reports Medicine, Volume *6*

Supplemental information

Dysfunctional one-carbon metabolism identifies vitamins B_6, B_9, B_{12}, and choline as neuroprotective in glaucoma

James R. Tribble, Vickie H.Y. Wong, Kelsey V. Stuart, Glyn Chidlow, Alan Nicol, Anne Rombaut, Alessandro Rabiolo, Anh Hoang, Pei Ying Lee, Carola Rutigliani, Tim J. Enz, Alessio Canovai, Emma Lardner, Gustav Stålhammar, Christine T.O. Nguyen, David F. Garway-Heath, Robert J. Casson, Anthony P. Khawaja, Bang V. Bui, and Pete A. Williams

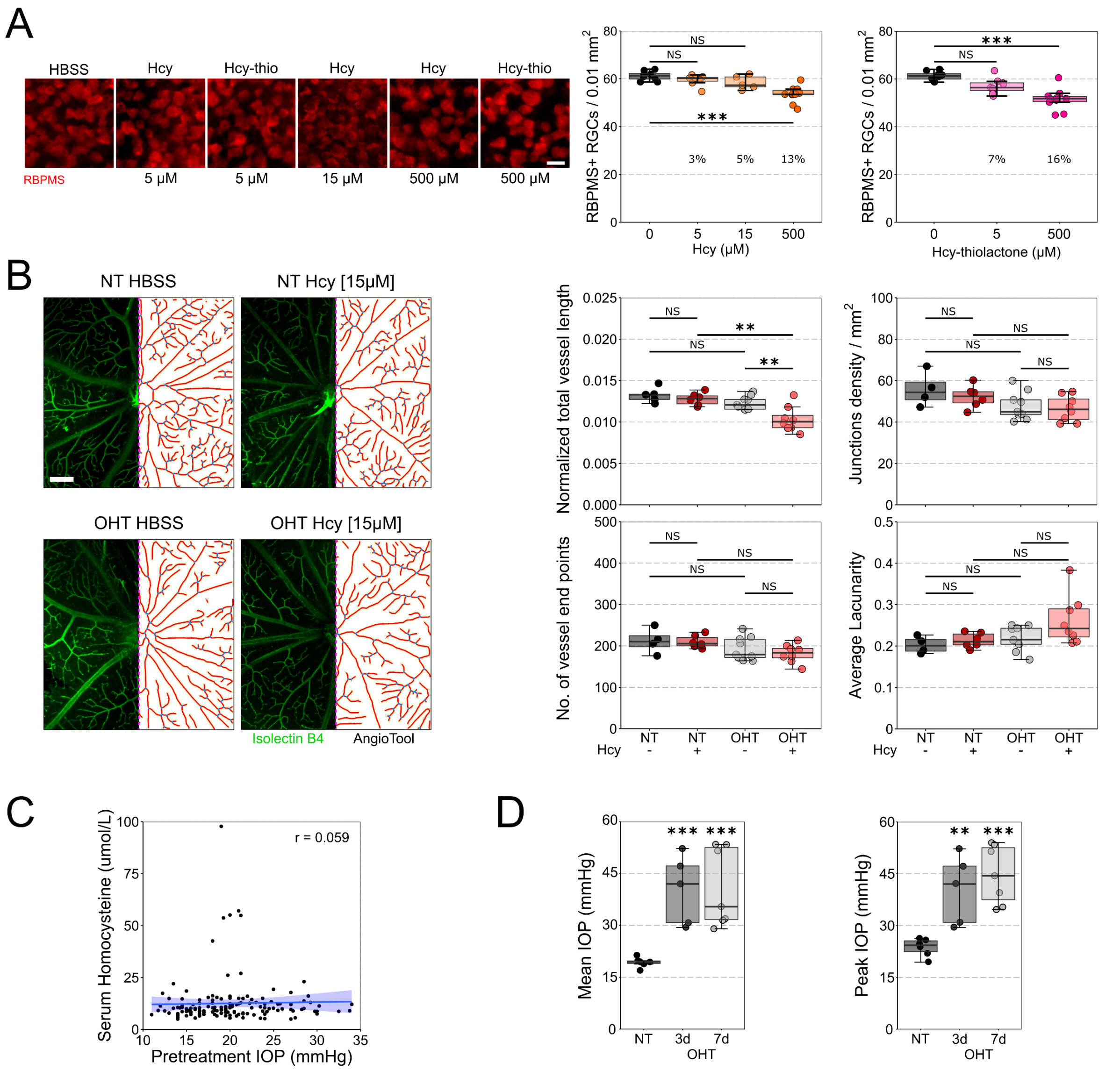

Figure S1: Additional results for the effects of homocysteine on retinal health, related to Figure 1 and 2. A) Mice received an intravitreal injection of homocysteine (Hcy) or homocysteine-thiolactone (Hcy-thiol) to yield a concentration in the vitreous of 5 µM, 15 µM or 500 µM. At 7 days post injection, RGC density was not significantly changed by Hcy at 5 µM or 15 µM, but was significantly reduced by Hcy at 500 µM compared to HBSS only (vehicle) controls. This was repeated for Hcy-thiol (HBSS, *n* = 8 retina; Hcy-5µM, *n* = 7 retina; Hcy-15µM, *n* = 5 retina; Hcy-500µM, *n* = 10 retina; Hcy-thiol-5µM, *n* = 9 retina; Hcy-thiol-500µM, *n* = 10 retina). **B**) Rats received an intravitreal injection of HBSS or homocysteine (Hcy; to a final concentration of 15 µM) 3 days prior to OHT induction. Blood vessel morphology (Isolectin B4+) in the central retina was reconstructed and analyzed (AngioTool). Normalized vessel length was significantly reduced in OHT-Hcy eyes relative to NT-HCY and OHT-HBSS eyes, while junction density (*blue spots*), the number of vessel end points, and lacunarity were not significantly changed. Together, this suggests a shrinkage of small capillaries without vessel drop-out (NT-HBSS, *n* = 4 retina; NT-Hcy, *n* = 6 retina; OHT-HBSS, *n* = 9 retina; OHT-Hcy, *n* = 8 retina). **C**) To determine whether serum homocysteine was associated with higher IOP we performed a linear regression of baseline IOP and baseline serum homocysteine for all patients in the UKGTS cohort and identified no significant association (r = 0.059, *P* = 0.753). Shaded area = confidence interval (*n* = 147 patients). Related to Figure 2, **D**) Rats were induced with OHT for 3 days (3d) or 7 days (7d) or remained NT. Mean and peak IOP were significantly greater in OHT eyes than in NT eyes, and there was no significant difference between 3 and 7 days (NT, *n* = 6 eyes; OHT-d3, *n* = 6 eyes; OHT-d7, *n* = 7 eyes).Eyes were paraffin embedded and sectioned for immunofluorescent labelling. Scale bar = 20

μm in A and 250 μm in B**.** * = $P < 0.05$, ** = $P < 0.01$, *** = $P < 0.001$, NS = $P > 0.05$. For box plots, the center hinge represents the median with upper and lower hinges representing the first and third quartiles; whiskers represent 1.5 times the interquartile range.

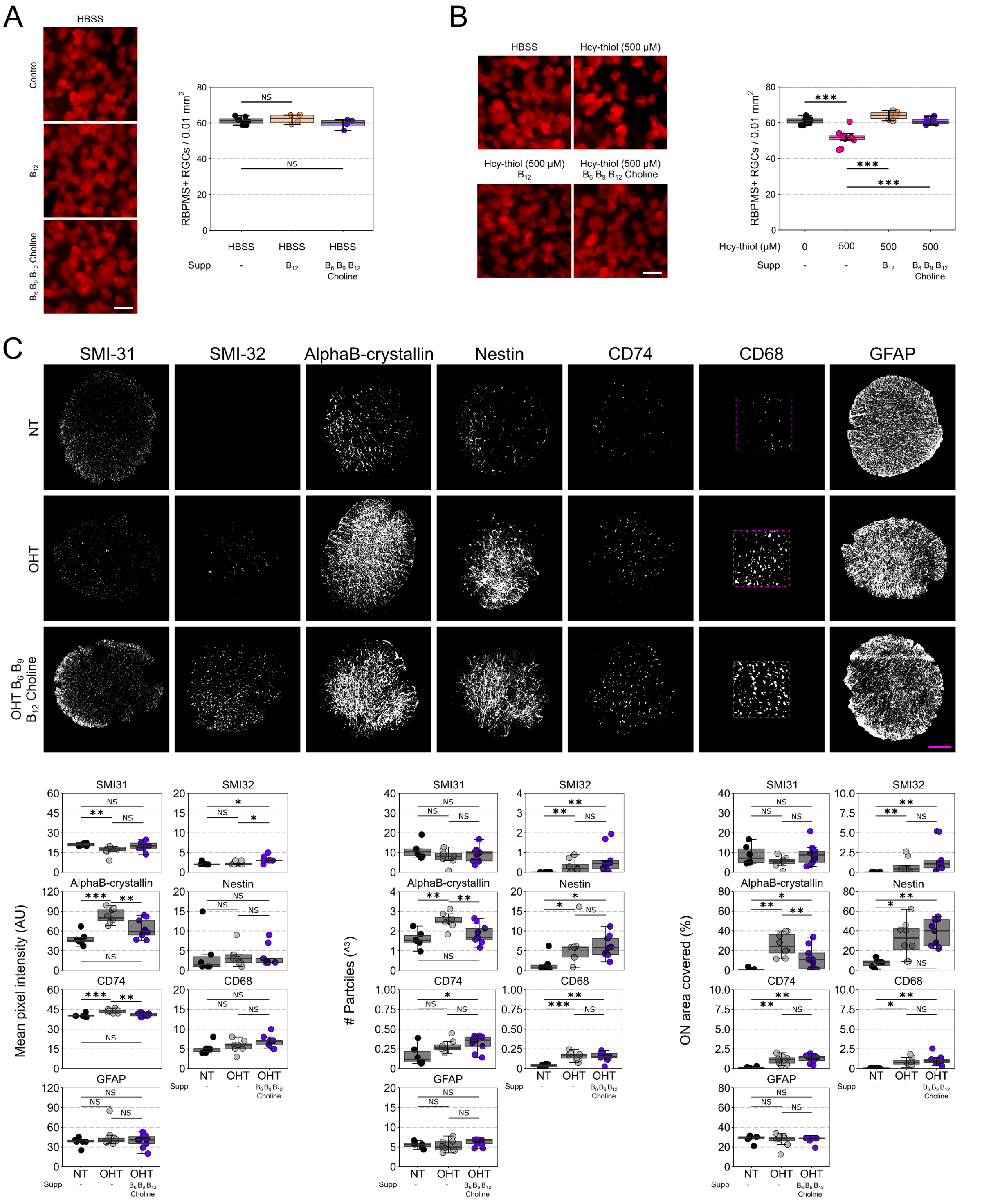

Figure S2: Additional results for the effects of B_6, B_9, B_{12}, and Choline on neurodegeneration in the mouse model of elevated ocular homocysteine and rat bead model, related to Figure 4. A-B) Mice received either 20 μg/kg/day vitamin B_{12} only or B_{12} with 4.5 mg/kg/day vitamin B_6, 1.5 mg/kg/day vitamin B_9, and 750 mg/kg/d Choline in their drinking water 7 days prior to intravitreal injection with either HBSS () or Homocysteine-thiolactone (Hcy-thiol; to a final concetration of 500 μM in the vitreous). **A**) Supplementation had no effect on RGC density after 14 days (HBSS-vehicle, *n* = 8 retina; HBSS-B_{12}, *n* = 4 retina; HBSS-B_6/B_9/B_{12}/Choline, *n* = 4 retina). **B**) Hcy-thio resulted in a significant decrease in RGC density 7 days after injection, which was completely prevented by vitamin B_{12} only or B_6, B_9, B_{12}, and Choline (7 days pre-treatment, continuing for the 7 days post injection; HBSS-vehicle, *n* = 8 retina; Hcy-thiol, *n* = 10 retina; Hcy-thiol-B_{12}, *n* = 6 retina; Hcy-thiol-B_6/B_9/B_{12}/Choline, *n* = 8 retina). **C**) Rats received the same doses 7 days prior to OHT induction and

optic nerves were collected following 14 days. IF/IHC labeling of SMI31 (healthy axons), SMI32 (degenerating axons), Alphaβ-crystallin and Hsp27 (stress), and Cd74, Cd68, and GFAP (inflammation) was performed and quantified by mean pixel intensity, the number of disconnected particles, and the percentage of the ON cross sectional area covered by positive labelling. We identified significant protection of SMI31 intensity by B_6, B_9, B_{12}, and Choline, but no prevention of SMI32 accumulation supporting a potential delay to axon degeneration. Alphaβ-crystallin was significantly reduced in B_6, B_9, B_{12}, and Choline relative to untreated OHT, supporting reduced stress. Markers of inflammation were generally not improved by B_6, B_9, B_{12}, and Choline relative to untreated OHT except for the intensity of Cd74 labelling, which was significantly reduced by B_6, B_9, B_{12}, and Choline (HT-vehicle n = 6 ONs; OHT-vehicle n = 8 ONs; OHT-B_6/B_9/B_{12}/choline n = 9 ONs) Scale bar = 20 µm in A and B, 100 µm in C**.** * = $P < 0.05$, ** = $P < 0.01$, *** = $P < 0.001$, NS = $P > 0.05$. For box plots, the center hinge represents the median with upper and lower hinges representing the first and third quartiles; whiskers represent 1.5 times the interquartile range.

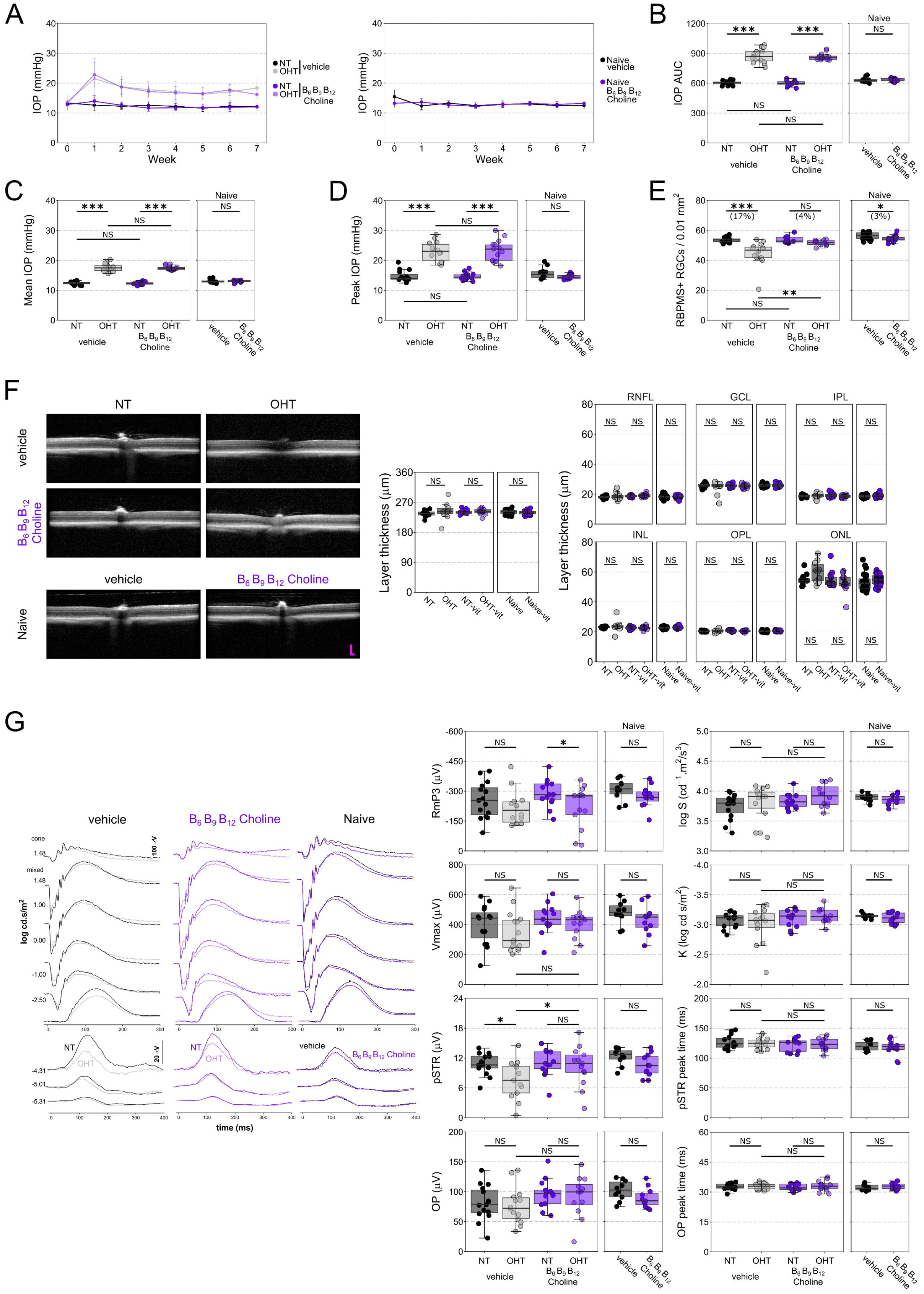

Figure S3: Additional results for the effects of B6, B9, B12, and Choline on neurodegeneration in the mouse circumlimbal suture model, related to Figure 4. A) Mice received 4.5 mg/kg/day vitamin B_6, 1.5 mg/kg/day vitamin B_9, 20 µg/kg/day vitamin B_{12}, and 750 mg/kg/d Choline (or vehicle only) in drinking water 1 week prior to OHT induction unilateral OHT (unilateral circumlimbal suture),

and continuing for the 7 week duration of OHT. Two naïve cohorts of mice were either vehicle treated or received B_6, B_9, B_{12}, and Choline. **A-D**) OHT was maintained for 7 weeks and the area under curve of the the IOP profile (**B**), the mean IOP (**C**), and the peak IOP (**D**) were not significantly altered by supplementation in OHT or NT eyes (*n* = 20 eyes from 10 mice, Naïve-vehicle; *n* = 22 eyes from 11 mice, Naïve-B_6/B_9/B_{12}/Choline; for *n* of other conditions see Figure 4). **E**) RGC density was significantly reduced in untreated OHT eyes, and this was completely protected against by B_6, B_9, B_{12}, and Choline. In naïve B_6, B_9, B_{12}, and Choline treated animals, RGC density was significantly reduced but the biological relevance of this is likely limited given the 3% difference to untreated naïve controls (*n* = 20 retina from 10 mice, Naïve-vehicle; *n* = 22 retina from 11 mice, Naïve-B_6/B_9/B_{12}/Choline; for *n* of other conditions see Figure 4). **F**) *In vivo* OCT imaging was performed to assess changes to retinal morphology. There were no significant changes in total retinal thickness or individual layer thicknesses across all conditions, consistent with mild OHT induced degeneration and lack of obvious toxic effects from supplementation (*n* = 24 eyes from 12 mice, Naïve-vehicle; *n* = 22 eyes from 11 mice, Naïve-B_6/B_9/B_{12}/Choline; *n* = 12 eyes NT-vehicle paired with *n* = 10 eyes OHT-vehicle from 12 mice ; *n* = 13 eyes NT-B_6/B_9/B_{12}/Choline paired with *n* = 11 eyes OHT-B_6/B_9/B_{12}/Choline from 13 mice). **G**) *In vivo* electrophysiological function was assessed by ERG. In naïve animals, B_6, B_9, B_{12}, and Choline had no effect on function. In untreated OHT eyes, pSTR amplitude was significantly decreased, and this was prevented in B_6, B_9, B_{12}, and Choline treated animals, supporting a protection to RGC specific visual function. Supplementation did cause a reduction in RmP3 amplitude, in treated eyes suggesting a decrease in photoreceptor scotopic responses (*n* = 10 eyes from 10 mice, Naïve-vehicle; *n* = 11 eyes from 11 mice, Naïve-B_6/B_9/B_{12}/Choline; for *n* of other conditions see Figure 4). Scale bar = 200 µM in F. * = $P < 0.05$, ** = $P < 0.01$, *** = $P < 0.001$, NS = $P > 0.05$. For box plots, the center hinge represents the median with upper and lower hinges representing the first and third quartiles; whiskers represent 1.5 times the interquartile range.

Trait	Source	Number of participants	Participant ethnicity	SNPs included in the instrumental variable
Exposure				
Homocysteine	van Meurs (2013) [S1]	44,147	European	14
Outcome				
Macular retinal nerve fiber layer thickness	Currant (2021) [S2]	31,434	European	-
Macular ganglion cell-inner plexiform layer thickness	Currant (2021) [S2]	31,434	European	-
Intraocular pressure	Khawaja (2018) [S3]	139,555	European	-
Vertical cup-disc ratio (adjusted for vertical disc diameter)	Han (2021) [S4]	111,724	European	-
Primary open-angle glaucoma	Gharahkhani (2021) [S5]	216,257	European	-

Table S1: Details of summary-level data used for Mendelian randomization analyses, related to Figure 1. SNP, single nucleotide polymorphism.

**MR method**	**mRNFL (μm)**		**mGCIPL (μm)**		**IOP (mmHg)**		**vCDR**		**POAG**	
	Beta (95% CI)	*P*	Beta (95% CI)	*P*	Beta (95% CI)	*P*	Beta (95% CI)	*P*	Odds ratio (95% CI)	*P*
**Homocysteine**										
IVW	-0.21 (-0.49, 0.08)	0.16	-0.30 (-0.81, 0.21)	0.25	0.03 (-0.16, 0.22)	0.77	0.00 (-0.01, 0.00)	0.39	1.12 (0.93, 1.27)	0.06
Weighted median	-0.18 (-0.57, 0.21)	0.37	-0.46 (-0.98, 0.07)	0.09	-0.06 (-0.21, 0.08)	0.40	0.00 (-0.01, 0.01)	0.71	1.13 (0.98, 1.30)	0.11
MR-Egger	-0.15 (-0.81, 0.51)	0.65	-0.55 (-1.73, 0.63)	0.36	0.09 (-0.35, 0.53)	0.70	0.00 (-0.02, 0.01)	0.66	1.02 (0.78, 1.35)	0.86
MR-PRESSO	-	-	-	-	-0.09 (-0.18, 0.00)	0.07	0.00 (-0.01, 0.00)	0.16	-	-

Table S2: Results of Mendelian randomization analyses, related to Figure 1. MR-PRESSO produces estimates after removal of significant outlying variants; if there are no significant outliers, the estimate is the same as that from the IVW method. IOP, intraocular pressure; IVW, inverse-variance weighted; MR, mendelian randomization; mRNFL, macular retinal nerve fiber layer; MR-PRESSO, Mendelian Randomization Pleiotropy RESidual Sum and Outlier; mGCIPL, macular ganglion cell-inner plexiform layer; POAG, primary open-angle glaucoma; vCDR, vertical cup-disc ratio.

SNP	Chr	BP	RA	EA	EAF	Nearest gene	Homocysteine		mRNFL		mGCIPL		IOP		vCDR		POAG	
							Beta	SE	Beta	SE	Beta	SE	Beta	SE	Beta	SE	Beta	SE
rs1801133	1	11856378	G	A	0.34	*MTHFR*	0.1583	0.0070	-0.0161	0.0382	-0.0771	0.0502	-0.0100	0.0136	-0.0003	0.0014	0.0190	0.0145
rs4660306	1	45978675	C	T	0.33	*MMACHC*	0.0435	0.0070	-0.0169	0.0381	-0.0545	0.0501	-0.0006	0.0135	0.0005	0.0014	0.0347	0.0135
rs2275565	1	237048676	T	G	0.79	MTR	0.0542	0.0090	0.0131	0.0441	-0.0084	0.0579	0.0158	0.0159	-0.0006	0.0016	-0.0049	0.0155
rs1047891	2	211540507	C	A	0.33	*CPS1*	0.0860	0.0080	0.0397	0.0388	0.1024	0.0510	-0.0255	0.0141	-0.0019	0.0015	0.0310	0.0139
rs548987	6	25869371	G	C	0.13	*SLC17A3*	0.0597	0.0100	0.0003	0.0517	0.1015	0.0679	-	-	-	-	-	-
rs9369898	6	49382193	G	A	0.62	*MUT*	0.0449	0.0070	-0.0188	0.0376	-0.0424	0.0494	-0.0050	0.0133	-0.0015	0.0014	0.0318	0.0133
rs42648	7	89977760	A	G	0.60	*GTPB10*	0.0395	0.0070	-0.0565	0.0371	-0.0434	0.0488	0.0042	0.0132	0.0008	0.0014	-0.0021	0.0131
rs1801222	10	17156151	G	A	0.34	*CUBN*	0.0453	0.0070	-0.0654	0.0374	-0.0324	0.0492	0.0150	0.0133	0.0028	0.0014	-0.0019	0.0135
rs12780845	10	17223244	G	A	0.65	*CUBN*	0.0529	0.0090	-0.0017	0.0392	-0.0406	0.0515	-0.0185	0.0138	-0.0012	0.0014	0.0063	0.0139
rs7130284	11	89148372	T	C	0.93	*NOX4*	0.1242	0.0130	0.1057	0.0689	0.1190	0.0905	-0.0091	0.0242	0.0008	0.0023	-0.0060	0.0235
rs2251468	12	121405126	A	C	0.35	*HNF1A*	0.0510	0.0070	0.0689	0.0383	0.0172	0.0503	0.0025	0.0136	0.0002	0.0014	0.0049	0.0134
rs154657	16	89708096	G	A	0.47	*DPEP1*	0.0963	0.0070	-0.0466	0.0367	-0.1124	0.0482	0.0709	0.0133	-0.0014	0.0014	0.0016	0.0133
rs838133	19	49259529	G	A	0.45	*FUT2*	0.0422	0.0070	0.0270	0.0375	0.1379	0.0493	-	-	0.0004	0.0017	0.0061	0.0137
rs234709	21	44486964	T	C	0.55	*CBS*	0.0718	0.0070	0.0259	0.0365	0.0044	0.0479	-0.0101	0.0130	-0.0021	0.0012	-0.0158	0.0142

Table S3: Details of SNPs included in the instrumental variables and their associations with glaucoma and related traits, related to Figure 1. rs1801133 (highlighted in green) is located in the methylenetetrahydrofolate reductase (MTHFR) gene region, a key enzyme in homocysteine metabolism. This SNP has the single strongest association with plasma homocysteine levels and is often used as an instrumental variable in MR analyses. BP, base position (build 37); Chr, chromosome; EA, effect allele; EAF, effect allele frequency; IOP, intraocular pressure; mGCIPL, macular ganglion cell-inner plexiform layer; mRNFL, macular retinal nerve fiber layer; POAG, primary open-angle glaucoma; RA, reference allele; SE, standard error; SNP, single nucleotide polymorphism; vCDR, vertical cup-disc ratio.

MR method	mRNFL		mGCIPL		IOP		vCDR		POAG	
	Statistic	*P*	Statistic	*P*	Statistic	*P*	Statistic	*P*	Statistic	*P*
**Homocysteine**										
**IVW**										
Cochran's *Q* statistic	13.3 (13)	0.43	25.0 (13)	0.02	37.0 (11)	<0.001	23.3 (12)	0.026	16.3 (12)	0.18
I^2 statistic	2.2%	-	48.0%	-	70.3%	-	48.4%	-	26.6%	-
**MR-Egger**										
Rucker's *Q'* statistic	13.3 (12)	0.35	24.5 (12)	0.02	36.7 (10)	<0.001	23.2 (11)	0.016	15.6 (11)	0.16
I^2_{GX} statistic	95.3%	-	95.3%	-	95.6%	-	93.2%	-	95.1%	-
Intercept	0.00	0.86	0.02	0.64	-0.01	0.77	0.00	0.92	0.01	0.47
**MR-PRESSO**										
Global test	-	0.48	-	0.03	-	<0.001	-	0.038	-	0.23
Number of outliers	0	-	0	-	1	-	1	-	0	-
Distortion test	-	-	-	-	131.1%	0.27	32.8%	0.70	-	-

Table S4: Tests of heterogeneity, directional pleiotropy and regression dilution statistics, related to Figure 1. IOP, intraocular pressure; IVW, inverse-variance weighted; MR, mendelian randomization; mRNFL, macular retinal nerve fiber layer; MR-PRESSO, Mendelian Randomization Pleiotropy RESidual Sum and Outlier; mGCIPL, macular ganglion cell-inner plexiform layer; POAG, primary open-angle glaucoma; vCDR, vertical cup-disc ratio.

Intake	mRNFL (μm)			mGCIPL (μm)			Glaucoma		
	Beta	(95% CI)	*P*	Beta	(95% CI)	*P*	OR	(95% CI)	*P*
**Vitamin B6 (mg)**		(*n* = 11,771)			(*n* = 11,751)			(*n* = 27,191)	
Per SD increase	-0.05	(-0.15, 0.06)	0.39	-0.10	(-0.21, 0.02)	0.10	1.01	(0.89, 1.15)	0.86
Q1 (<1.64)		Reference			Reference			Reference	
Q2 (1.64–1.99)	**0.20**	**(0.01, 0.40)**	**0.042**	0.14	(-0.12, 0.39)	0.30	0.93	(0.70, 1.24)	0.61
Q3 (1.99–2.40)	0.11	(-0.10, 0.31)	0.32	0.05	(-0.22, 0.32)	0.73	1.12	(0.83, 1.49)	0.46
Q4 (>2.40)	-0.04	(-0.27, 0.20)	0.76	-0.12	(-0.43, 0.18)	0.43	1.00	(0.72, 1.39)	0.99
P (trend)			0.63			0.38			0.71
**Folate (μg)**		(*n* = 11,771)			(*n* = 11,751)			(*n* = 27,191)	
Per SD increase	-0.07	(-0.15, 0.01)	0.10	-0.09	(-0.20, 0.02)	0.10	1.04	(0.93, 1.17)	0.49
Q1 (<247.2)		Reference			Reference			Reference	
Q2 (247.2–302.5)	**0.21**	**(0.01, 0.40)**	**0.035**	0.05	(-0.20, 0.31)	0.68	0.91	(0.68, 1.21)	0.51
Q3 (302.5–366.3)	**0.22**	**(0.02, 0.42)**	**0.034**	0.19	(-0.08, 0.45)	0.16	0.92	(0.68, 1.23)	0.57
Q4 (>366.3)	-0.07	(-0.29, 0.16)	0.56	-0.13	(-0.43, 0.16)	0.37	1.06	(0.77, 1.45)	0.72
P (trend)			0.66			0.63			0.68
**Vitamin B12 (μg)**		(*n* = 11,771)			(*n* = 11,751)			(*n* = 27,191)	
Per SD increase	-0.02	(-0.09, 0.06)	0.63	-0.01	(-0.11, 0.08)	0.78	0.91	(0.81, 1.01)	0.08
Q1 (<4.35)		Reference			Reference			Reference	
Q2 (4.35–5.74)	-0.07	(-0.27, 0.12)	0.46	0.00	(-0.25, 0.26)	0.98	0.85	(0.64, 1.12)	0.24
Q3 (5.74–7.46)	-0.02	(-0.22, 0.18)	0.86	0.20	(-0.06, 0.46)	0.13	0.83	(0.62, 1.09)	0.18
Q4 (>7.46)	-0.07	(-0.28, 0.13)	0.49	0.07	(-0.20, 0.33)	0.63	0.81	(0.61, 1.08)	0.16
P (trend)			0.64			0.37			0.18

Table S6: Dietary intake of B_6, B_9, B_{12} with glaucoma related-traits, related to Figure 4. Dietary intake of B_6, folate (B_9) and B_{12} multivariable linear (for mRNFL and GCIPL thickness) and logistic (for glaucoma status) regression analyses. CI, confidence interval; mGCIPL, macular ganglion cell-inner plexiform layer; mRNFL, macular retinal nerve fiber layer; OR, odds ratio; Q, quartile; SD, standard deviation.

REAGENT	SOURCE	IDENTIFIER
Ahcy PrimePCR SYBR Green Assay	Bio-rad	10025636
Ahcyl1 PrimePCR SYBR Green Assay	Bio-rad	10025636
Dnmt1 PrimePCR SYBR Green Assay	Bio-rad	10025636
Dnmt3a PrimePCR SYBR Green Assay	Bio-rad	10025636
Dnmt3b PrimePCR SYBR Green Assay	Bio-rad	10025636
Mtr PrimePCR SYBR Green Assay	Bio-rad	10025636
Mtrr PrimePCR SYBR Green Assay	Bio-rad	10025636
Mat1a PrimePCR SYBR Green Assay	Bio-rad	10025636
Mat2b PrimePCR SYBR Green Assay	Bio-rad	10025636
Cbs PrimePCR SYBR Green Assay	Bio-rad	10025636
Cth PrimePCR SYBR Green Assay	Bio-rad	10025636
Dhfr PrimePCR SYBR Green Assay	Bio-rad	10025636
Shmt1 PrimePCR SYBR Green Assay	Bio-rad	10025636
Shmt2 PrimePCR SYBR Green Assay	Bio-rad	10025636
Mthfr PrimePCR SYBR Green Assay	Bio-rad	10025636
Gapdh PrimePCR SYBR Green Assay	Bio-rad	10025636

Table S7: Continuation of Key resources table – Oligonucleotides, related to Key resources table. qPCR primer details.

Supplemental reference list

S1. van Meurs JB, Pare G, Schwartz SM, Hazra A, Tanaka T, Vermeulen SH, et al. Common genetic loci influencing plasma homocysteine concentrations and their effect on risk of coronary artery disease. Am J Clin Nutr. 2013;98(3):668-76.

S2. Currant H, Hysi P, Fitzgerald TW, Gharahkhani P, Bonnemaijer PWM, Senabouth A, et al. Genetic variation affects morphological retinal phenotypes extracted from UK Biobank optical coherence tomography images. PLoS Genet. 2021;17(5):e1009497.

S3. Khawaja AP, Cooke Bailey JN, Wareham NJ, Scott RA, Simcoe M, Igo RP, et al. Genome-wide analyses identify 68 new loci associated with intraocular pressure and improve risk prediction for primary open-angle glaucoma. Nat Genet. 2018;50(6):778-82.

S4. Han X, Steven K, Qassim A, Marshall HN, Bean C, Tremeer M, et al. Automated AI labeling of optic nerve head enables insights into cross-ancestry glaucoma risk and genetic discovery in >280,000 images from UKB and CLSA. Am J Hum Genet. 2021;108(7):1204-16.

S5. Gharahkhani P, Jorgenson E, Hysi P, Khawaja AP, Pendergrass S, Han X, et al. Genome-wide meta-analysis identifies 127 open-angle glaucoma loci with consistent effect across ancestries. Nat Commun. 2021;12(1):1258.
